# Single‐cell transcriptomics reveals a senescence‐associated IL‐6/CCR6 axis driving radiodermatitis

**DOI:** 10.15252/emmm.202115653

**Published:** 2022-07-04

**Authors:** Mor Paldor, Orr Levkovitch‐Siany, Dana Eidelshtein, Revital Adar, Claes D Enk, Yitzhak Marmary, Sharona Elgavish, Yuval Nevo, Hadar Benyamini, Inbar Plaschkes, Shiri Klein, Alex Mali, Stefan Rose‐John, Amnon Peled, Eithan Galun, Jonathan H Axelrod

**Affiliations:** ^1^ The Goldyne‐Savad Institute of Gene Therapy Hadassah Hebrew University Hospital Jerusalem Israel; ^2^ Department of Dermatology Hadassah Hebrew University Hospital Jerusalem Israel; ^3^ Info‐CORE, Bioinformatics Unit of the I‐CORE Hebrew University of Jerusalem Jerusalem Israel; ^4^ Department of Pathology Hadassah Hebrew University Hospital Jerusalem Israel; ^5^ Institut für Biochemie Christian‐Albrechts‐Universität zu Kiel Kiel Germany

**Keywords:** radiodermatitis, alopecia, senescence, IL‐6, CCR6, Chromatin, Transcription & Genomics, Immunology, Skin

## Abstract

Irradiation‐induced alopecia and dermatitis (IRIAD) are two of the most visually recognized complications of radiotherapy, of which the molecular and cellular basis remains largely unclear. By combining scRNA‐seq analysis of whole skin‐derived irradiated cells with genetic ablation and molecular inhibition studies, we show that senescence‐associated IL‐6 and IL‐1 signaling, together with IL‐17 upregulation and CCR6^+^‐mediated immune cell migration, are crucial drivers of IRIAD. Bioinformatics analysis colocalized irradiation‐induced IL‐6 signaling with senescence pathway upregulation largely within epidermal hair follicles, basal keratinocytes, and dermal fibroblasts. Loss of cytokine signaling by genetic ablation in IL‐6^−/−^ or IL‐1R^−/−^ mice, or by molecular blockade, strongly ameliorated IRIAD, as did deficiency of CCL20/CCR6‐mediated immune cell migration in CCR6^−/−^ mice. Moreover, IL‐6 deficiency strongly reduced IL‐17, IL‐22, CCL20, and CCR6 upregulation, whereas CCR6 deficiency reciprocally diminished IL‐6, IL‐17, CCL3, and MHC upregulation, suggesting that proximity‐dependent cellular cross talk promotes IRIAD. Therapeutically, topical application of Janus kinase blockers or inhibition of T‐cell activation by cyclosporine effectively reduced IRIAD, suggesting the potential of targeted approaches for the treatment of dermal side effects in radiotherapy patients.

## Graphical abstract

Irradiation‐induced alopecia and dermatitis (IRIAD) represent two of the most prominent side effects of radiotherapy in the skin. In this study, Paldor *et al* applied scRNA‐seq analysis to identify driving factors of IRIAD in order to facilitate the development of targeted therapeutic approaches for its prevention.

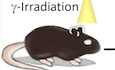

**The paper explained**

**Problem**

Radiotherapy‐treated patients with cancer frequently suffer from side effects including irradiation‐induced alopecia and dermatitis (IRIAD). In addition to psychological stress, these side effects can also generate severe chronic radiation injury, including the delayed formation of ulcers, fibrosis, telangiectasia, and opiate‐resistant chronic pain. Moreover, the serious complications in wound healing of the skin following radiotherapy constitute a major impediment to reconstructive surgery. The mechanism(s) underlying IRIAD remain poorly understood, thus limiting the development of effective rationally targeted therapies.

**Results**

scRNA‐seq analysis of an IRIAD model in mice reveals pivotal cellular and molecular cross‐interactions involving inflammatory and senescence‐associated cytokine signaling pathways, including IL‐6, IL‐1, and type 17‐mediated cytokines (IL‐17, IL‐22). Genetic or pharmacological blockade of these pathways strongly reduced major effects of irradiation, including acanthosis, alopecia, and senescence. Moreover, IL‐6 signaling blockade prevented IL‐17 IL‐22, CCL20, and CCR6 upregulation, and the infiltration of CD3^+^ T cells to degenerating hair follicles, and, vis‐a‐versa; IR‐induced IL‐6 upregulation was strongly dependent upon IL‐17 upregulation, CCR6‐dependent immune cell infiltration upon loss of HF immune privilege. Moreover, inhibition of T‐cell activation by treatment with cyclosporine A, or inhibition of STAT3‐mediated signaling by topical application of pharmacological inhibitors of Janus kinases also effectively prevented IRIAD.

**Impact**

The findings of this study point to the clinical potential of cytokine, chemokine, and T cell‐targeted therapeutics for the prevention of IRIAD in radiotherapy patients, in particular via topical application of low‐molecular‐weight pharmaceuticals.

## Introduction

Irradiation‐induced alopecia (hair loss) and radiodermatitis (IRIAD) are two of the most common and psychologically stressful side effects in radiotherapy patients (Lawenda *et al*, 2004; Hymes *et al*, 2006; Ryan, 2012). Radiodermatitis occurs in about 95% of radiotherapy patients and ranges in severity from mild erythema to moist desquamation and ulceration (Hymes *et al*, 2006; Ryan, 2012). Late or chronic irradiation (IR)‐induced injury, including the delayed formation of ulcers, fibrosis, telangiectasias, and opiate‐resistant chronic pain, can also spontaneously appear months and even years after IR exposure (Hymes *et al*, 2006; Brown & Rzucidlo, 2011; Ryan, 2012). Moreover, problematic wound healing in irradiated skin constitutes a major surgical impediment and remains a substantial therapeutic challenge (Gieringer *et al*, 2011; Haubner *et al*, 2012). Despite recent improvements in skin‐sparing radiation technology, such as intensity‐modulated radiation therapy, alopecia, and injury to the skin remain markedly problematic (Salvo *et al*, 2010; McQuestion, 2011). Surprisingly, although the effects of irradiation on the skin and hair follicles have been known for more than a century, their underlying molecular and cellular mechanism(s) remain poorly understood, thus limiting the development of effective, rationally targeted therapies (Malkinson & Keane, 1981; Hymes *et al*, 2006; Ryan, 2012).

The cellular events encompassing IRIAD have been described in considerable depth (Ryan, 2012). Immediate damage to hair follicle stem cells and basal keratinocytes that follows the burst of IR‐released free radicals leading to persistent double‐stranded DNA breaks is largely thought to initiate the inflammation‐mediated process of IR‐induced skin injury (Ryan, 2012). Subsequent infiltration of immune cells, including neutrophils, macrophages, and T cells to irradiated skin, and hyperkeratosis constitute commonly recognized hallmarks of acute IR‐induced skin injury (Muller & Meineke, 2007; Holler *et al*, 2009; Ryan, 2012). Nevertheless, much of the cellular and molecular interactions leading to IRIAD remain unclear. In consideration of the given complexity of the skin, involving numerous cell types, we have applied quantitative single‐cell RNA sequence (scRNA‐seq) analysis to an established IRIAD model in mice (Vegesna *et al*, 1988; Burdelya *et al*, 2012; Marmary *et al*, 2016) in order to elucidate molecular and cellular events that drive IRIAD.

## Results

### A single‐cell transcriptome atlas of irradiated skin

**Figure 1:**
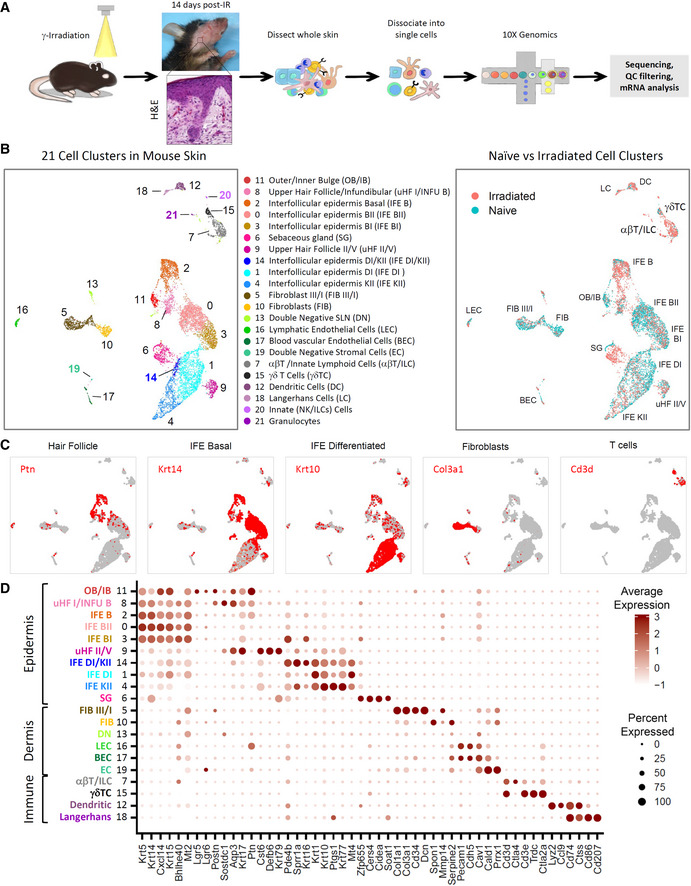
scRNA‐seq analysis reveals heterogeneity in radiodermatitis ASchematic of scRNA‐seq experimental workflow.BUMAP projection of 8,805 cells from irradiated and naïve mouse skin integrated into 21 clusters. Cells are colored by the assigned cluster (middle). (Right) UMAP projection showing cluster composition according to cell origin in naïve or irradiated skin.CNaïve expression of the hair follicle (*Ptn*), IFE Basal (*Krt14*), IFE Differentiated (*Krt10*), fibroblast (*Col3a1*), and immune T cell (*Cd3d*) transcripts visualized by UMAP across scRNA‐seq datasets from integrated naïve and irradiated skin.DDot plot depicting integrated naïve‐irradiated clusters according to skin cell‐type‐specific marker expression in the naïve cells.Data information: See also Fig EV1 and Appendix Figs S1–S6.

Irradiation (15 Gy) of mice (C57BL/6) to the head and neck generated initial external signs of skin injury with the appearance of serous exudates on the chin and throat beginning at about 8 days post‐IR that progressed in severity with time and spread upward to the cheeks and downward to include the chest. Radiodermatitis reached maximal levels about 2 weeks post‐IR and subsided thereafter (Fig 1A). The appearance of alopecia followed that of radiodermatitis, reaching maximal levels about 3–4 weeks post‐IR, and hair growth returned to roughly normal levels by weeks 7–8, but with striking loss of hair color (depigmentation), as reported previously (Inomata *et al*, 2009; Fig 1A and Appendix Fig S1A). A similar outcome resulted following exposure of mice to 30 Gy irradiation administered in fractionated doses over 5 consecutive days (5 × 6 Gy; Appendix Fig S1B). Importantly, different areas of skin displayed varying degrees of irradiation sensitivity, with the ventral neck, the chest, and the muzzle exhibiting the highest sensitivity levels, thus appearing to closely recapitulate the presentation in human radiotherapy patients (Lawenda *et al*, 2004; Hymes *et al*, 2006; Brown & Rzucidlo, 2011). Histological analysis of H&E stained skin thin sections from mice 2–3 weeks post‐IR revealed striking morphological changes in both the epidermal and dermal layers, which included keratinocyte hyperplasia, and acanthosis involving a fourfold thickening of the epidermis, capillary stasis, edema, and infiltration of immune cells (Fig 1A and Appendix Fig S1C).

To dissect the cellular and molecular events in IRIAD, we performed scRNA‐seq analysis on unsorted living cells isolated separately from the whole skin of either naïve or irradiated (15 Gy) mice taken 14 days post‐IR (Fig 1A). Dissected skin samples were separated into epidermal and dermal layers prior to enzymatic dissociation and treated with a cocktail of protease inhibitors upon termination to enhance cell viability. Viable dissociated cell populations were then pooled and characterized using the 10× Genomics^®^ platform for random capture and 3′ sequencing analysis of cDNAs. In total, 8,805 sequenced skin cells (*n* = 4 biological replicates per experimental group) met our quality control and inclusion criterion. Using the 10× Genomics platform, we obtained about 5,135 and 3,670 transcriptomes for naïve and irradiated skin cells, respectively, totaling about 25,000 detected genes. Cells with less than 200 expressed genes or more than 5% mitochondrial gene expression were excluded. Unsupervised cluster analysis using the Seurat software package (Stuart *et al*, 2019) segregated the naïve and irradiated skin cells into 21 distinct clusters which were overlaid and visualized in two‐dimensional space by Uniform Manifold Approximation and Projection (UMAP; Becht *et al*, 2018; Fig 1B and C).

Cell clusters were classified according to cell type and lineage within naïve cell populations by comparison with previously reported and validated scRNA‐seq datasets for epidermal (Joost *et al*, 2016) and dermal (Guerrero‐Juarez *et al*, 2019) cell populations using GSEA analysis. Immune and stromal cell populations were further classified by comparison with community‐based immune‐related gene sets of the Immunological Genome Project (Heng *et al*, 2008). In this way, we assigned clusters to epidermal or dermal/stromal layers and identified specific cell types, including immune cells.

Epidermal‐derived clusters, identified by gene expression patterns and GSEA, included hair follicle (HF) inner bulge cells clustered together with outer bulge (OB/IB, Cl‐11) cells expressing *Lgr5/Lgr6/Postn/Ptn*; upper hair follicle (uHF) I/III and infundibulum B (INFU B) cells showing a likeness to OB cells (uHF I/INFU, Cl‐8) and expressing *Postn/Sostdc1/Aqp3*; uHF II with uHF V (uHF II/V; Cl‐9) cells expressing *Krt79*/*Defb6/Krt17*/*Cst6*; and sebaceous gland (SG, Cl‐6) cells expressing marker genes *Cers4*/*Cidea*/*Soat1*/Zfp655 (Joost *et al*, 2016; Fig 1C and D, and Appendix Fig S2). Cluster analysis by GSEA also identified epidermal cells divided into three distinct interfollicular epidermis basal (IFE‐B) cell clusters (IFE‐B, Cl‐2; IFE‐BII, Cl‐0; and IFE‐BI, Cl‐3) characterized by *Krt5/Krt14/Krt15/Cxcl14/Mt2/Bhlhe40* and distinguished between themselves by *Ptn/Pde4b/Krt16* expression (Joost *et al*, 2016); and three distinct clusters of differentiated or keratinized IFE cells (IFE‐DI, Cl‐1; IFE‐DI/KII, Cl‐14; and IFE‐KII, Cl‐4) expressing *Krt1/krt10/Krt77/Ptsg1/Mt4*. Two distinct clusters representing stromal fibroblast (FIB) populations were also identified: FIB‐III/I (Cl‐5) expressing *Col1a1/Col3a1/CD34/Dcn*, with strong similarities to FIB types 1, 2, and 3 as defined previously (Guerrero‐Juarez *et al*, 2019); and FIB (Cl‐10) expressing *Spon1/Serpine2*, with remote resemblances to FIB types 1, 2, and 3 and also sharing similarities with skin lymph node reticular fibroblasts (Fig 1D, and Appendix Figs S2 and S3). GSEA and ImmGen database comparisons identified four cutaneous stromal endothelial clusters expressing *Pecam1/Cdh5/Cav1/Cald1* that included blood endothelial cells (BEC, Cl‐17), lymphatic endothelial cells (LEC, Cl‐16), and two small, endothelial‐related clusters with similarities to stromal double‐negative cells (DN, Cl‐13, and EC, Cl‐19; Link *et al*, 2007; Fig 1C and D, and Appendix Fig S3).

Four distinct immune cell populations, including dendritic cells (Cl‐12; *Lyz2/Ccl9/Ctss/Cd74*), Langerhans cells (Cl‐18; *Cd74*, *Ctss*, *Cd86*, *Cd207*), resident αβT cells that appeared to cluster together with innate lymphoid cells (αβT/ILC, Cl‐7; *Cd3d*, *Ctla4*), and γδT cells (Cl‐15; *Cd3d*, *Cd3e*, *Trdc*, *Ctla2a*) were identified by comparisons using GSEA or ImmGen databases (Fig 1C and D, and Appendix Fig S3). Further analysis of naïve αβT/ILC (CL‐7) cells for individual marker genes indicated that most cells in the cluster expressed at least one *Cd3* allele (*d*,*e*,*g*), *Cd28*, and/or *Trac*, suggesting an adaptive T‐cell lineage. However, about 10% (5/46) of the cells in the cluster expressed ILC‐associated genes (*Klrk1*, *Runx*, or *Fuca1*; Koues *et al*, 2016) and were negative for T‐cell markers, thus suggesting that these cells may represent ILCs (ILC1, ILC3, or NK cells; Appendix Fig S4). Additionally, 10% (4/46) of naive αβT/ILC cells were identified as *Foxp3* ^+^ Tregs (Appendix Fig S4). Dendritic cells of Cl‐12 appeared largely of cDC2 (*Itgam*, *Sirpa*) lineage that cluster together with monocyte‐derived macrophages (*Adgre1*, *C1qc*, *Mafb*; Guilliams *et al*, 2014; Saba *et al*, 2022; Appendix Fig S4).

A comparison of the effect of irradiation on cluster populations revealed substantial IR‐induced changes in cell numbers within specific clusters. These included striking relative increases (> 4‐fold) in populations of IFE‐B and SG cells, and in two immune clusters, αβT/ILC cells and dendritic cells (Appendix Table S1). Relative increased population sizes in the epidermal clusters aligned with the upregulation of cell cycle markers, Ki67 (*Mki67*), and geminin (*Gmnn*), indicating a strong mitotic response within these HF clusters drives the keratinocyte hyperplasia observed in H&E thin sections of irradiated skin (Appendix Fig S5). In contrast, relatively few αβT/ILC cells showed upregulation of cell cycle markers, suggesting that infiltration may largely contribute to their increased number. Additionally, following irradiation almost all αβT/ILC cells express one or more of the T‐cell marker genes, substantially outnumbering the ILC population (Appendix Fig S4). FoxP3^+^ Treg cell numbers remained relatively stable but were reduced to about 1% (5/365) of the cluster against the accumulating T‐helper population (Appendix Fig S4). The composition of cluster‐12 (dendritic cells) also appeared to shift following irradiation showing higher numbers of *Adgre1*, *C1qc*, *Mafb*, and *Ly6c2* expressing cells that overlap with higher numbers of cells expressing *Itgam* and *Sirpa*, perhaps suggesting an influx of monocyte‐derived macrophages and dendritic cells in addition to resident cDC2 cells (Appendix Fig S4). In addition, two novel minor cell clusters representing innate NK/ILCs immune cells (Cl‐20) and granulocytes (Cl‐21) appeared exclusively following irradiation (Appendix Fig S3). At the same time, notable reductions (> 2‐fold) were evident in epidermal OB/IB, IFE‐BII, and IFE‐DI cells population sizes, as well as in both stromal fibroblast clusters, whereas other cluster populations remained proportionally unchanged.

Bioinformatics comparison of irradiated versus naïve cell clusters using QIAGEN Ingenuity Pathway Analysis (IPA) revealed multiple significant physiological and molecular pathways that characterize the cellular responses to IR. Among the most prominently ranked pathways (i.e. pathways with the highest significance: −log(B‐H *P*‐value) > 1.3, and *z*‐scores > 2 or < −2) appearing across both epidermal and dermal cell clusters were pathways associated with aging‐related functions, including *Mitochondrial Dysfunction*, *Senescence Pathways* upregulation, and senescence‐related *Sirtuin Signaling* downregulation (Lee *et al*, 2019; Table EV1). Together with the senescence‐associated pathways, IPA also revealed strong upregulation of multiple cytokine signaling pathways, including both IL‐6 and IL‐1 signaling, as reflected in their positive IPA z‐score values. Notably, upregulation of IL‐6 and IL‐1 signaling pathways, which are essential cytokines contributing to the senescence‐associated secretory phenotype (SASP; He & Sharpless, 2017), conspicuously overlapped within irradiated epidermal and dermal clusters that also displayed highly significant upregulation in *Senescence Pathways* (Table EV1). Curiously, we also noted that, although not conventionally thought to be associated with senescence, IPA reported significant upregulation of *IL‐17 Signaling*, reminiscent of *IL‐17A Signaling in Airway Cells*, *Th17 Activation*, and *the Role of IL‐17A in Psoriasis* (Table EV1) that appeared to parallel those of IL‐6 signaling and senescence pathways in many clusters. Interrogation of the scRNA‐seq datasets by CellPhoneDB analysis (Efremova *et al*, 2020) concurred with the IPA findings showing significantly increased potential ligand–receptor interactions in irradiated skin (Appendix Fig S6). Thus, irradiation increased the levels of multiple ligand–receptor interactions that spanned across multiple cell clusters, particularly within FIB‐III/I, BEC, IFE‐B, and uHF‐I/INFU‐B cells, and, as described below in detail, included those involving IL‐6, IL‐1, IL‐17, and their respective cognate receptors (Appendix Fig S6).

**Figure EV1:**
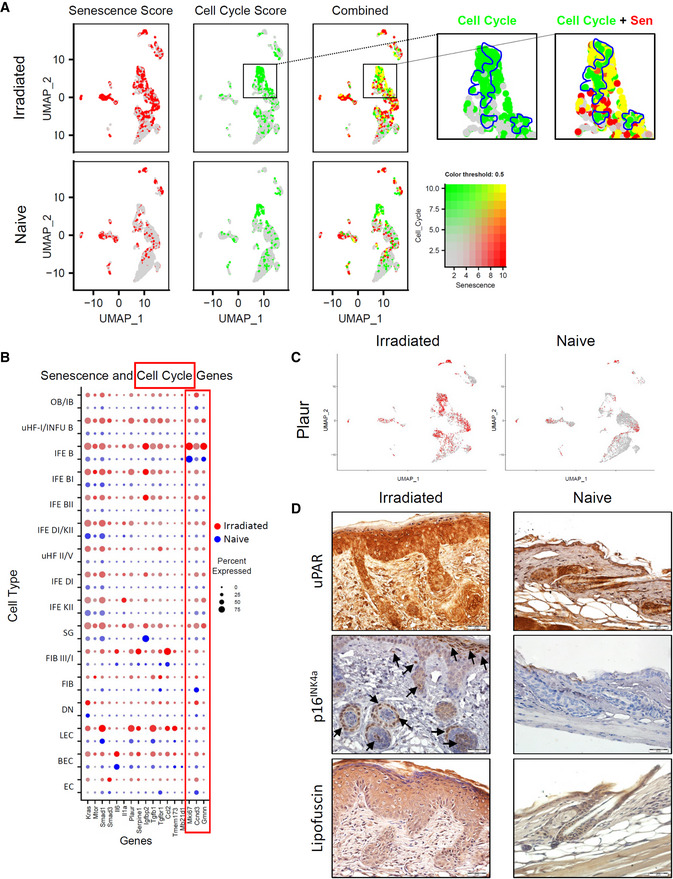
(Related to Fig 1). Relative mutually exclusive senescence and cell cycle scoring in naïve and irradiated epidermal cells ARelative expression and co‐localization of Senescence Score (red) and Cell Cycle Score (*Mki67*, *Gmnn*, *Ccna1*; green) in integrated skin cells by blended UMAPs. Blowup: Regions of IEF‐2 and uHF‐I/INFU‐B showing exclusive mitotic activity (outlined in blue).BDot plot depicting the relative expression of selected IPA‐defined senescence‐related markers and cell cycle markers (framed in red) (*x*‐axis) in naïve (blue dots) and irradiated (red dots) scRNA‐seq skin cell clusters.CUMAP plots depicting relative expression and localization of urokinase‐type plasminogen activator receptor (*Plaur*) mRNA in irradiated and naïve skin‐derived clusters.DImmunostaining and histochemical staining (brown) of urokinase‐type plasminogen activator receptor (uPAR), p16^INK4a^, and lipofuscin (SenTraGor®) in paraffin‐embedded skin thin sections from ventral neck and upper chest region of naïve and irradiated (15 Gy) wild type mice taken at 21 days post‐IR. p16^INK4a^ immunostaining appears as nuclear and/or cytoplasmic staining (Arrows) in IFE keratinocytes, in cells comprising discrete hair follicle layers, and in distinct cells in the dermis. Scale bars, 20 μm.

To localize senescence pathway upregulation within the skin scRNA‐seq cell clusters, we employed an arbitrary “*Senescence Score*” based on the combined relative expression of select IPA designated senescence‐related genes (*Kras*, *Mtor*, *Smad1*, *Smad3*, *Il6*, *Il1a*, *Plaur*, *Serpine1*, *Igfbp2*, *Tgfb1*, *Tgfbr1*, *Ccl2*, *Tmem173*, *Mb21d1*) to stain the clusters (Fig EV1A and B). This depiction suggested that cellular senescence, which in naive skin is associated with roughly half of the FIB‐III/I and FIB dermal fibroblasts, expands following irradiation to include nearly all FIB‐III/I fibroblasts, and many HF cells, including some OB/IB, uHF/INFU‐B, and SG cells and most basal keratinocytes (IFE‐B, ‐BI, ‐BII), largely in agreement with the IPA analysis (Table EV1).

Expression of *Plaur* mRNA, which encodes the urokinase‐type plasminogen activator receptor (uPAR), a protein that is broadly induced on the surface of senescent cells (Amor *et al*, 2020), mirrored the increased senescence score, particularly within irradiated HF and basal keratinocytes (uHF/INFU‐B, IFE‐B) and SG‐derived cells (Fig EV1C). Anti‐uPAR immunostaining confirmed the substantially increased IR‐induced uPAR protein expression in keratinocytes particularly within basal keratinocytes and degenerating hair follicles, but notably less so in highly differentiated keratinocytes of the stratum granulosum, and above (Fig EV1D). Similarly, strong p16^INK4a^ immunostaining was evident within multiple epidermal layers of the irradiated skin, particularly within differentiated IFE keratinocytes, and with lesser intensity in basal keratinocytes and distinct cell layers of degenerating hair follicles, but was substantially weaker within the most highly differentiated keratinocytes of the stratum granulosum, stratum lucidum, and stratum corneum (Fig EV1D). Distinct p16^INK4a^ immunostaining was also evident in individual cells within irradiated dermis but was absent overall within naïve control skin (Fig EV1D). Histochemical immunostaining with SenTraGor^®^ for lipofuscin, which accumulates in senescent cells as a by‐product of the aging process (Georgakopoulou *et al*, 2013), confirmed the presence of cellular senescence in irradiated skin keratinocytes and fibroblasts, with notably less staining in the stratum lucidum and stratum corneum (Fig EV1D), concordant with the uPAR and p16^INK4a^ immunostaining and with the IPA analysis.

Importantly, cells scoring positive for senescence pathways appeared to be largely distinct from those with a positive “*Cell Cycle Score*” (*Mki67*, *Gmnn*, *Ccnd3*), with the exception of select irradiated IFE‐B cells that appeared to be positive for both (Fig EV1A and B), perhaps suggesting an intermediate or reversible state of senescence in these cells. Thus, simultaneously, both senescence‐associated factors and keratinocyte hyperplasia appeared to be inherent to the pathological landscape of radiodermatitis.

### IL‐6/Stat3 signaling is crucial for IRIAD

**Figure 2:**
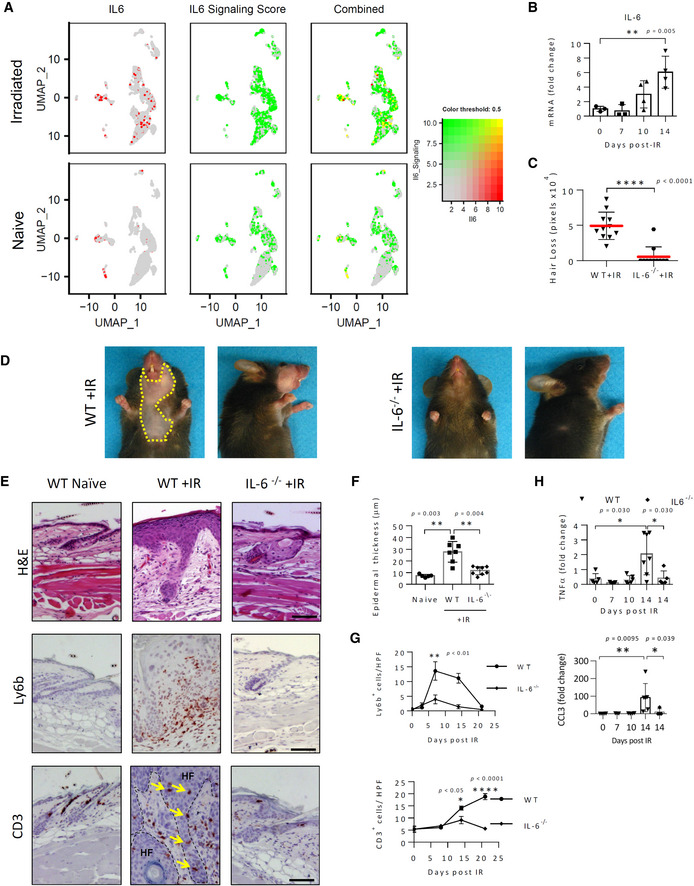
IL‐6 signaling deficiency reduces alopecia and radiodermatitis in irradiated mice ARelative expression and co‐localization of *IL6* mRNA and IL‐6 Signaling Scoring in integrated skin cells by blended UMAPs.BQuantification by real‐time qPCR analysis of *Il6* mRNA in the skin of naïve and irradiated (15 Gy) WT mice at indicated times, (*n* = 3–6).CQuantification of ventral hair loss area 21 days post‐IR (15 Gy) in WT and IL‐6^−/−^ mice, (*n* = 11).DPhotographic images showing hair loss (area demarked by yellow dashed line) 21 days post‐IR (15 Gy) in wild type (WT) and IL‐6^−/−^ mice.EHistochemical (H&E) and immunostaining (red) showing skin morphology, and T‐cell (CD3^+^) and neutrophil (Ly6b^+^) infiltration in WT or IL‐6^−/−^ mice before (naïve), and 14 days (H&E, Ly6b^+^), or 21 days (CD3^+^) post‐IR. Infiltrating CD3^+^ T cells (yellow arrows) within degenerating hair follicles (HF; dashed lines) are indicated. Scale bars, 100 μm.FQuantification of epidermal thickness at 14 days post‐IR (*n* = 5–8).GKinetics of neutrophil (Ly6b^+^; *n* = 3–9) and T‐cell (CD3^+^; *n* = 3) infiltration.HQuantification by real‐time qPCR analysis of *Tnfa* and *Ccl3* mRNAs in whole skin of WT and IL‐6^−/−^ mice at indicated times post‐IR (15 Gy), (*n* = 5–7). Data information: Data represent mean ± SD (B, C, F, H) or mean ± SEM (G). *P < 0.05, **P < 0.01, **P < 0.01, ****P < 0.001 by one‐way ANOVA with Dunnett's multiple comparison test (B), two‐tailed Mann–Whitney test (C, F, H), or two‐way ANOVA with Bonferroni's post hoc multiple comparisons test (G); five independent experiments (See also Fig EV3 and Appendix Figs S7 and S9). Source data are available online for this figure.

Since IL‐6 upregulation is reportedly one of the early events in the molecular response to irradiation (Tartakovsky *et al*, 1993; Fedorocko *et al*, 2002; Marmary *et al*, 2016) and is considered a predictor of IR‐associated complications in radiotherapy patients (Chen *et al*, 2001; Meirovitz *et al*, 2010), we next examined the role of IL‐6 in IRIAD in greater depth. ScRNA‐seq analysis of cell clusters from untreated skin showed relatively high constitutive IL‐6 mRNA expression in naive dermal BEC and FIB‐III/I fibroblasts that increased substantially 14 days post‐IR within FIB‐III/I cells to include almost all cells within the cluster (Fig 2A and Appendix Fig S7A). At this time, IL‐6 expression also appeared to be upregulated within numerous cells of multiple epidermal clusters, including within IFE‐B, ‐BI, ‐BII, and IFE‐DI cells. Quantitative real‐time PCR analysis confirmed the overall upregulation in IL‐6 mRNA levels in irradiated skin showing initial signs of elevation at about 10 days post‐IR and increasing by day 14 to about sixfold above baseline levels (Fig 2B).

To depict the upregulation of IL‐6 signaling pathways within the skin cell clusters, we utilized an arbitrary “*IL‐6 Signaling Score*” based on the combined relative differential expression of select IPA designated genes (*Akt1*, *Il6*, *Il18*, *Il33*, *Map2k1*, *Rala*, *Rela*, *Socs1*, *Socs3*, *Vegfa*). Appendix Fig S7B shows expression levels of the individual genes composing the IL‐6 signaling score according to cell type. Interestingly, as depicted in the cluster map, although IR‐induced IL‐6 mRNA upregulation localized to relatively few cells within the keratinocyte clusters, “*IL‐6 Signaling*” appeared to be widespread (Fig 2A), suggesting that a paracrine mechanism underlies the spread of IL‐6 signaling within these clusters. The IL‐6 signaling score also largely aligned with IPA analysis according to their significance (−log(B‐H *P*‐val)) and/or *z*‐scores (Table EV1), with the exceptions of IFE‐KII keratinocytes, dendritic cells, and Langerhans cells, which also appeared to be positive in naïve mice and perhaps did not increase substantially following irradiation. CellPhoneDB analysis similarly indicated that IL‐6 expression in naïve skin was primarily limited to BEC and FIB‐III/I cells but spread following irradiation to include IFE‐B, IFE‐BI, and IFE‐DI cells (Appendix Fig S7C). Potential significant interactions with IL‐6 receptor (IL‐6R), resulting mainly from IL‐6 produced from these cells, also appeared to spread following irradiation to include significant interactions within BEC, dendritic, OB/IB, SG, and the majority of IFE‐B cell clusters.

**Figure EV2:**
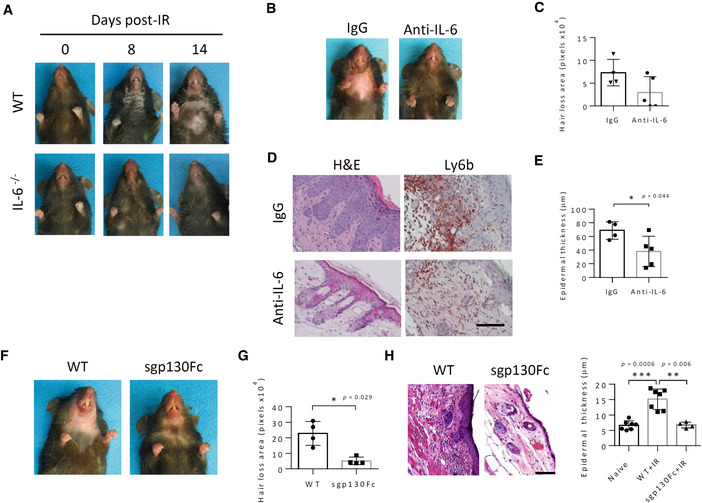
(Related to Fig 2). IL‐6 signaling blockade reduces hair loss and dermatitis in irradiated mice APhotographic images depicting the kinetics of radiodermatitis and hair loss in ventral views of representative wild‐type mice irradiated (15 Gy) to the head and neck. Times (in days) post‐IR are indicated. Serous exudates appear under the chin at 8 days post‐IR with desquamation and alopecia beginning at about 14 days post‐IR.BPhotographic images showing ventral alopecia in wild‐type mice treated with neutralizing IL‐6 mAb (Anti‐IL‐6) or control mAb (IgG; 200 μg, i.p) prior to and 8 days following irradiation (14 Gy) to the head and neck. Two independent experiments.CQuantification of the area of hair loss of mice in (B) at 14 days post‐IR, (*n* = 4–5). Data are individual mice. Two independent experiments.DHistochemical (H&E) and immunostaining of skin thin sections from mice in (B) showing morphology and neutrophil (Ly6b) infiltration (red staining). Scale bar, 50 μm.EQuantification of epidermal thickness in specimens from (D), (*n* = 4–5).FPhotographic images showing ventral alopecia in wild‐type mice (WT) and sgp130Fc transgenic mice irradiated (14 Gy) to the head and neck 21 days post‐IR. Five independent experiments.GQuantification of the area of hair loss of mice in (F), (*n* = 4).HHistochemical (H&E) staining of skin thin sections from mice in (F) and quantification of the epidermal thickness (right), (*n* = 4–7). Scale bar, 100 μm. Data information: Data are mean ± SD. *P < 0.05, **P < 0.01, ***P < 0.001 by two‐tailed Student's *t*‐test (E), or Mann–Whitney test (G, H). Source data are available online for this figure.

To elucidate the role of IL‐6 in IRIAD, we next determined the effect of irradiation on the skin of IL‐6 *knockout* (IL‐6^−/−^) mice (Kopf *et al*, 1994). The physiological function of IL‐6 in dermal skin cell populations is unclear since naïve IL‐6^−/−^ mice are phenotypically indistinguishable from WT controls. However, following irradiation, IL‐6 deficiency substantially reduced early manifestations of inflammation, including the appearance of serous exudations in WT mice 7–10 days post irradiation (Fig EV2A). By day 21 post‐IR, IL‐6^−/−^ mice outwardly displayed about a 10‐fold reduction in alopecia with strongly diminished histologically evident hair follicle degeneration and acanthosis in comparison to irradiated WT controls (Fig 2C–F). IL‐6 deficiency strikingly reduced CD3^+^ T‐cell infiltration into both the dermis and, notably, within degenerating hair follicles, and reduced the neutrophil infiltration, commensurate with reductions in CCL3 and TNFα mRNA upregulation (Fig 2E–H).

Treatment of irradiated (14 Gy) WT mice with a neutralizing IL‐6 monoclonal antibody (mAb) largely recapitulated the effect of *IL6* gene ablation (Fig EV2B–E). Thus, anti‐IL‐6 mAb treatment produced substantial reductions in IR‐induced hair loss, acanthosis, and neutrophil infiltration, thus ruling out developmental aberrations as underlying factors in the phenotype of IL‐6^−/−^ mice. Similarly, specific blockade of IL‐6 signaling mediated via its soluble IL‐6R receptor (also called IL‐6 *trans*‐signaling) by ectopic expression of a recombinant sgp130 protein, sgp130Fc, in transgenic sgp130Tg mice (Rose‐John *et al*, 2006; Rabe *et al*, 2008; Tenhumberg *et al*, 2008) also substantially ameliorated IRIAD (Fig EV2F–H). Since sgp130 reportedly poorly blocks both autocrine, *classical* IL‐6 signaling (Jostock *et al*, 2001) and IL‐6 *trans*‐presentation (Heink *et al*, 2017), respectively, these findings substantiate the role of paracrine IL‐6 *trans*‐signaling in IRIAD. Interestingly, in this respect, in mice irradiated with 15 Gy, both neutralizing IL‐6 mAb and sgp130‐mediated *trans*‐signaling blockade were substantially less effective than total *IL6* ablation in ameliorating IRIAD.

**Figure 3:**
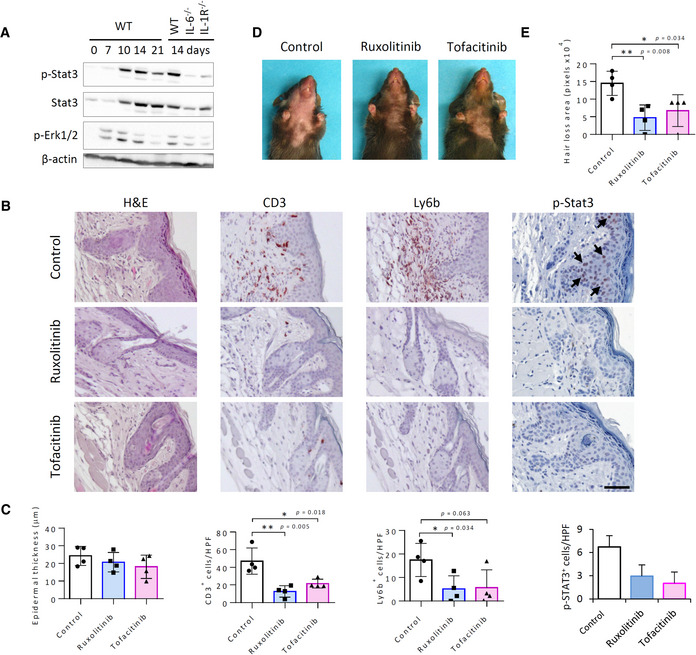
Blockade of STAT3 activation reduces IRIAD AWestern blot analysis shows phosphorylated STAT3 (p‐Stat3), Erk1 and Erk2 (p‐Erk1/2), and β‐Actin in the skin of WT, IL‐6^−/−^, and IL‐1R^−/−^ mice at indicated times (days) post‐IR (15 Gy).BHistochemical (H&E) and immunostaining (red) staining of T cells (CD3^+^), neutrophils (Ly6b^+^), and phosphorylated Stat3 (p‐Stat3; red nuclear staining, black arrows) in skin thin sections prepared at 14 days post‐IR from mice treated by daily topical application of aquosum (control), ruxolitinib, or tofacitinib following irradiation (15 Gy). Scale bar, 100 μm. (CD3 and Ly6b immunostaining images from Tofacitinib‐treated mice represent separate analyses performed on serial thin sections.)CQuantification of epidermal thickness, T cells (CD3^+^) and neutrophils (Ly6b^+^), and p‐STAT3^+^ cells in (B), (*n* = 4).DPhotographic images showing hair loss in mice at 14 days post‐IR (15 Gy) following treatment by daily topical application of aquosum (carrier control) or JAK inhibitors, ruxolitinib, or tofacitinib. Chest and neck hairs of mice in all groups were clipped prior to irradiation to enhance drug absorption.EQuantification of the area of ventral hair loss (in pixels) in mice in (D), (*n* = 4). Data information: Data represent mean ± SD. *P < 0.05, **P < 0.01, by two‐tailed Student's *t*‐test; two independent experiments (See also Appendix Fig S8). Source data are available online for this figure.

Commensurate with IL‐6 mRNA upregulation, western blot analysis indicated that levels of phosphorylated signal transducer and activator of transcription 3 (STAT3), a key mediator of IL‐6 signaling (Heinrich *et al*, 2003), were prominently elevated beginning at about 10 days post‐IR, and were strongly diminished in irradiated IL6^−/−^ mice (Fig 3A). A similar increase in ERK1/2 phosphorylation appeared by day 7. Immunostaining analysis in irradiated WT mouse skin also showed that IR‐induced STAT3 phosphorylation localized primarily to keratinocytes within degenerating HF and IFE cells in regions of acanthosis and was substantially blocked by topical application of small‐molecule JAK inhibitors, ruxolitinib, and tofacitinib (Fig 3B and C), which have been reported to help prevent hair loss in IFN‐γ‐mediated alopecia areata in humans (Xing *et al*, 2014). Treatment by the JAK inhibitors also significantly diminished both neutrophil and CD3^+^ T cell infiltration to the skin and substantially ameliorated hair loss, although a corresponding effect on acanthosis appeared marginal (Fig 3C–E). Increased STAT3 phosphorylation also correlated in part with the IR‐induced interferon‐γ (IFN‐γ) mRNA upregulation (Appendix Fig S8). However, on day 14 post‐IR, when IFN‐γ mRNA appeared to subside to baseline levels, phosphorylated STAT3 levels remained elevated and IL‐6 deficiency, which strongly reduced STAT3 phosphorylation, did not affect IFN‐γ mRNA upregulation. Together, these findings demonstrate that IL‐6/STAT3 signaling is an inherent mediator of IRIAD.

IPA‐defined IL‐6 signaling also appeared to overlap in part with the cell cycle response score (*Mki67*, *Gmnn*, and *Ccnd3*) that, in naïve skin clusters, localized primarily to basal keratinocytes (IFE‐B) cells and, following irradiation, spread to HF, basal cells (IFE‐B, IFE‐BI), and SG cells (Appendix Fig S9A). Importantly, loss or blockade of IL‐6 signaling, whether in IL‐6^−/−^, IL‐6 mAb treated, or sgp130Tg mice, also strongly reduced IR‐induced keratinocyte hyperplasia and acanthosis visible in H&E stained thin sections (Figs 2F, and EV2E and H). Immunofluorescence staining also showed substantially increased cell division (Ki67^+^) in HF and IFE keratinocytes of irradiated WT mice and was strongly reduced by the sgp130‐mediated trans‐signaling blockade, thus confirming that the keratinocyte hyperplasia is in part IL‐6 dependent (Appendix Fig S9B).

**Figure EV3:**
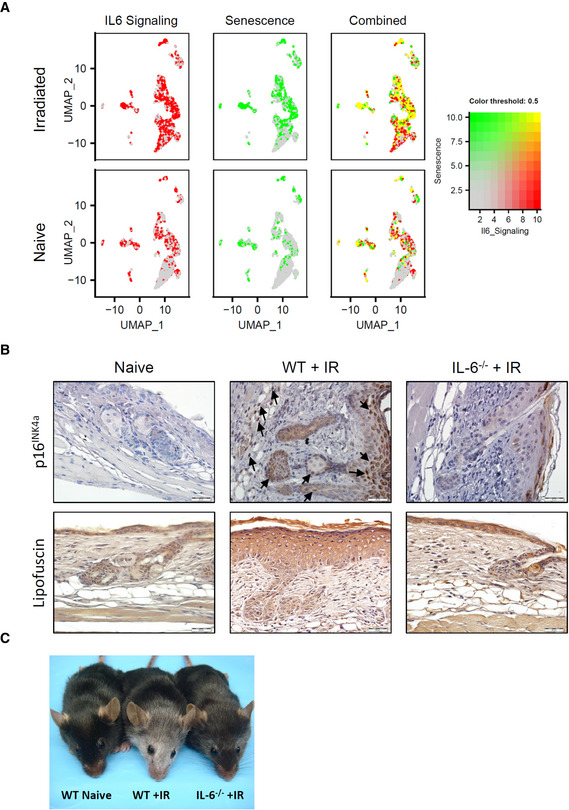
(Related to Fig 2). Substantial overlap of IL‐6 signaling and cellular senescence in irradiated mouse skin ARelative expression and co‐localization of IL‐6 Signaling (Red; see also Appendix Fig S7B) and Senescence Pathway (Green; see also Fig EV1B) scoring in integrated skin cells by blended UMAPs.BPhotographic images showing immunostaining of p16^INK4a^ (arrows) and lipofuscin (brown stain) in irradiated (15 Gy) IL‐6^−/−^ mice skin thin sections compared to irradiated wild type (WT) mice 21 days post‐IR WT. Scale bar, 20 μm.CPhotographic images showing hair depigmentation in representative naïve WT and irradiated (15 Gy) WT and IL‐6^−/−^ mice at 8 weeks post‐IR. (Four independent experiments.)

At the same time, scRNA‐seq and IPA analyses revealed a substantial overlap of IL‐6 signaling with senescence scoring present in irradiated skin cells, particularly within fibroblast (FIB‐III/I) and keratinocytes (uHF/INFU B, IFE‐B) populations (Table EV1 and Fig EV3A). Importantly, in agreement with its reported role in the maintenance of cellular senescence (Kuilman *et al*, 2008; Marmary *et al*, 2016), levels of senescence in both the epidermal and dermal layers were strikingly diminished in the skin of irradiated IL‐6^−/−^ mice, as indicated by p16^INK4a^ immunostaining and by immune‐histochemical staining for lipofuscin (Fig EV3B). Interestingly, we also observed that prominent loss of hair color (depigmentation) appearing about 2 months post‐IR in WT mice during subsequent cycles of hair follicle regrowth (Inomata *et al*, 2009) was also strikingly reduced in IL‐6^−/−^ mice (Fig EV3C). Since hair graying by ionizing irradiation is thought to result from irreversible DNA damage‐induced abrogation of melanocyte stem cell renewal, rather than by senescence or apoptosis (Inomata *et al*, 2009), we infer from these observations that IL‐6 signaling may underlie this process as well. From these findings, we conclude that IL‐6/STAT3 signaling is a crucial mediator of cellular and phenotypic changes in IRIAD.

### IL‐1 signaling is crucial for IRIAD

**Figure 4:**
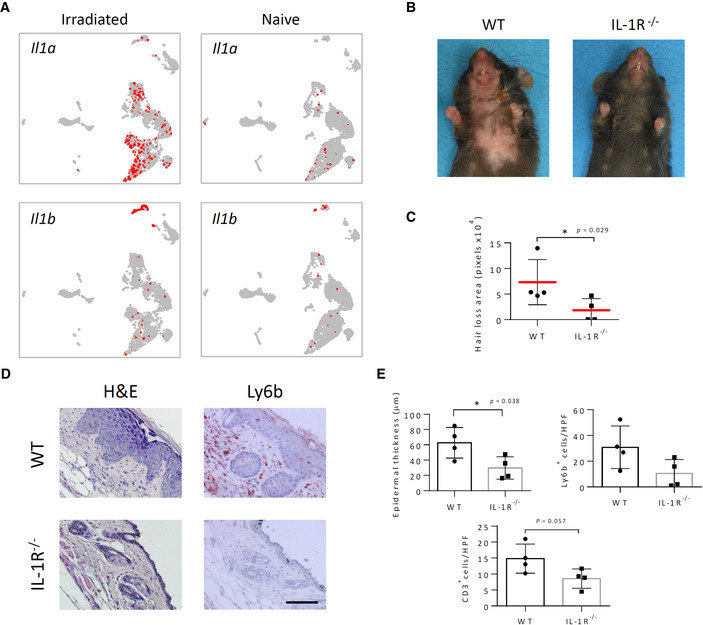
IL‐1R deficiency reduces irradiation‐induced alopecia and radiodermatitis in mice AUMAP plots depicting relative expression and localization of *Il1a* and *Il1b* mRNAs in irradiated and naïve skin‐derived cell clusters.BPhotographic images showing hair loss 14 days post‐IR (15 Gy) in WT and IL‐1R^−/−^ mice.CQuantification of ventral hair loss area 14 days post‐IR (15 Gy) in WT and IL‐1R^−/−^ mice, (*n* = 4).DHistochemical (H&E) and immunostaining (red) showing morphology and neutrophil (Ly6b) infiltration in the skin of mice in (B). Scale bar, 50 μm.EQuantification of epidermal thickness, neutrophils (Ly6b), and T cells (CD3^+^) in specimens in (D), (*n* = 4). Data information: Data represent mean ± SD. *P < 0.05, by two‐tailed Mann–Whitney test (C) or Student's *t*‐test (E); five independent experiments (See also Appendix Figs S10 and S11). Source data are available online for this figure.

ScRNA‐seq analysis also revealed strong IL‐1α mRNA upregulation following irradiation, appearing most notably in both basal and differentiated keratinocytes (IFE‐B, IFE‐DI/KII, IFE‐KII) and SG cells (Fig 4A), in agreement with previous observations (Luger *et al*, 1981). In contrast, IL‐1β mRNA, which in naïve skin is constitutively expressed in innate dendritic and Langerhans cells, was strongly upregulated following irradiation within Langerhans cells and infiltrating granulocytes (Fig 4A), as previously reported (Takashima & Bergstresser, 1996; Ryan, 2012). Total IPA‐defined IL‐1 signaling correspondingly appeared prominently upregulated (*z*‐score ≥2) within SG cells, in correlation with IL‐1α upregulation, but also within dermal FIB‐III/I cells (Table EV1), in which both IL‐1α and IL‐1β expression appear largely absent (Fig 4A). CellPhoneDB analysis concurred with this assessment, indicating that potential IL‐1α/IL‐1β and IL‐1 receptor (IL‐1R) interactions spread following IR to include most HF clusters and dermal fibroblasts (Appendix Fig S10). Importantly, complete disruption of both IL‐1α and IL‐1β signaling in IL‐1R *knockout* (IL‐1R^−/−^) mice strikingly reduced IR‐induced acanthosis, neutrophil infiltration, and hair loss (Fig 4B–E), thus demonstrating a critical role of IL‐1 signaling in IRIAD. Moreover, western blot analysis showed that disruption of IL‐1 signaling in IL‐1R^−/−^ mice also strongly diminished IR‐induced STAT3 phosphorylation (Fig 3A), suggesting a close upstream relationship between IL‐1 signaling and STAT3‐mediated signaling in IRIAD. In line with its role as a SASP factor, loss of IL‐1 signaling in irradiated IL‐1R^−/−^ mice also strongly reduced p16^INK4a^ upregulation in IFE keratinocytes, hair follicles, and dermal cells compared to irradiated WT controls (Appendix Fig S11A), and, similar to IL‐6 deficiency, strikingly reduced hair depigmentation that appears following hair follicle regrowth in irradiated WT mice (Appendix Fig S11B). The latter observation suggests that IL‐1 and IL‐6 signaling may collaborate to enforce DNA damage‐induced abrogation of melanocyte stem cell renewal (Inomata *et al*, 2009).

### Immune‐mediated IL‐17 response and IRIAD

**Figure EV4:**
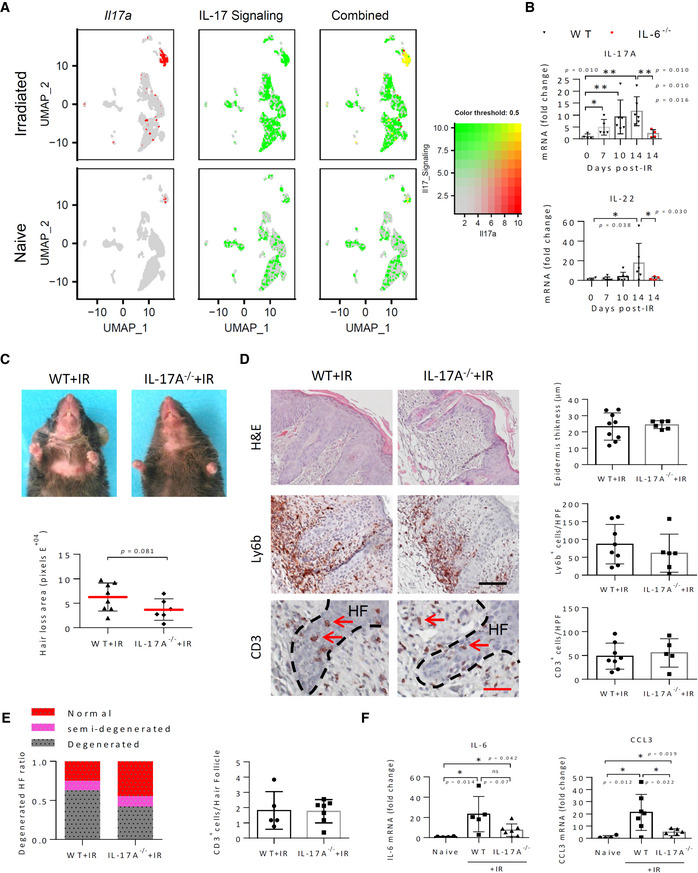
IL‐17A deficiency moderately alleviates IRIAD in mice ARelative expression and co‐localization of *Il17a* mRNA and IL‐17 Signaling in integrated skin cells by blended UMAPs.BQuantification by real‐time qPCR analysis of *Il17a* and *Il22* mRNAs in whole skin of naïve and irradiated (15 Gy) WT and IL‐6^−/−^ mice at indicated times post‐IR, (*n* = 4–7).CPhotographic images showing hair loss and radiodermatitis at 14 days post‐IR (15 Gy) in representative WT and IL‐17^−/−^ mice and quantification (below) of the area of ventral hair loss, (*n* = 6–8).DHistochemical staining (H&E), immunostaining (red), and quantification (right) of acanthosis and infiltration of neutrophils (Ly6b^+^) and T cell (CD3^+^; red arrows) to the skin and to the hair follicle (HF; dashed lines) in irradiated WT and mice in (C), (*n* = 6–7). Scale bars, 50 μm (black) and 25 μm (red).EQuantification of normal and degenerate hair follicles shown in H&E sections (left) and of T cell (CD3) infiltration into the hair follicle (HF; right) shown in (D), (*n* = 6–7).FQuantification by real‐time qPCR analysis of *Il6* and *Ccl3* mRNAs in the skin of WT and IL‐6^−/−^ mice before (naïve) and 14 days post‐IR (15 Gy, *n* = 4–7). Data information: Data represent mean ± SD. *P < 0.05, **P < 0.01 by two‐tailed Mann–Whitney test; two independent experiments (See also Appendix Fig S12).

The apparent overlap between IL‐6 signaling and the upregulation in IL‐17 signaling in many irradiated cell clusters as indicated by IPA (Table EV1), together with the prominent IL‐6‐dependent CD3^+^ T‐cell infiltration into degenerating hair follicles in irradiated skin prompted us to next explore the role of IL‐17 upregulation in IRIAD. ScRNA‐seq analysis showed that IL‐17A mRNA expression was strongly upregulated in almost all αβT/ILC cells and γδT cells found in irradiated skin (Fig EV4A). These IL‐17 expressing cells in the αβT/ILC cluster likely represent Th17 cells, since, as noted above, following irradiation nearly all of the cells in this cluster also appear to express one or more of the T‐cell marker genes, *Cd3*, *Cd28*, or *Trac* (Appendix Fig S4). Real‐time qPCR analysis of whole skin RNA confirmed the elevation in IL‐17A mRNA levels that increased six‐ to eightfold in the skin by day 14 post‐IR (Fig EV4B).

To visually map IL‐17 signaling within the skin cell clusters, we utilized an arbitrary “*IL‐17 Signaling Score*” reflecting the relative expression of a select gene set derived from the IPA‐designated “*IL‐17A Signaling in Airway Cells*” pathway (*Akt1*, *Akt2*, *Ccl11*, *Chuk*, *Ccl20*, *Cxcl3*, *Il17a*, *IL17f*, *Il17rc*, *Il19*, *Jak2*, *Map2k1*, *Map2k2*, *Map2k4*, *Mapk13*, *Mapk14*, *Nfkbia*, *Pik3r2*, *Pik3ca*, *Pik3r1*, *Pten*, *Rela*). Appendix Fig S12A shows expression levels of the individual IL‐17 signaling score component genes according to cell type. As expected, increased IPA‐defined IL‐17 signaling was evident in αβT/ILC cells and in some γδT cells, where it overlapped with IL‐17A mRNA upregulation, but also spread throughout epidermal keratinocytes and dermal FIB‐III/I fibroblasts (Fig EV4A). CellPhoneDB analysis similarly reported potential IL‐17A/IL‐17RAC ligand–receptor interactions of low intensity (≤ 0.37) within naïve cell clusters that increased substantially (≤ 1.8) following irradiation. These post‐IR interactions, which now involved IL‐17A expression from both αβT/ILC and γδT cells, spread to encompass most epidermal HF cells and keratinocytes (OB/IB, INFU B, uHF I/II/V, IFE BI/ BII, SG, IFE KII/DI), and many dermal stromal populations (FIB‐III/I, BEC, LEC) as well (Appendix Fig S12B). IL‐17A upregulation was accompanied by a roughly 30‐fold increase in IL‐22 mRNA expression (Fig EV4B) that co‐localized with IL‐17A mRNA mainly in αβT/ILC cells (Appendix Fig S12C), identifying some cells within this cluster as pathogenic Th17 T cells, although the presence of other related cell types, including non‐pathogenic Th17 cells and innate ILC3 cells, cannot be excluded (Yamazaki *et al*, 2008; Klose & Artis, 2016; Wu *et al*, 2018). Importantly, in irradiated IL‐6^−/−^ mice, expression of both *Il17a* and *Il22* mRNAs remained close to baseline levels (Fig EV4B), suggesting a critical IL‐6‐dependent role of IL‐17A and IL‐22 producing T/ILC cells in IRIAD.

To determine the contribution of IL‐17A to IRIAD, we assessed the effect of irradiation on IL‐17A *knockout* (IL‐17A^−/−^) mice. In comparison to WT controls, irradiated IL‐17A^−/−^ mice displayed only a moderate relief in hair follicle degeneration and hair loss of about 40% (*P* = 0.08), without apparent reductions in acanthosis or either neutrophil or CD3^+^ T cells infiltration (Fig EV4C–E). Curiously, although the levels of IR‐induced IL‐6 and CCL3 mRNAs appeared to be substantially reduced in IL‐17A^−/−^ mice, they apparently remained sufficiently elevated to support IRIAD (Fig EV4F). This was perhaps due to compensation by IL‐17F upregulation, which displayed expression and potential ligand–receptor interaction patterns similar to those of IL‐17A (Appendix Fig S12D and E).

### CCR6/CCL20‐mediated cell migration drives IRIAD in mice

**Figure 5:**
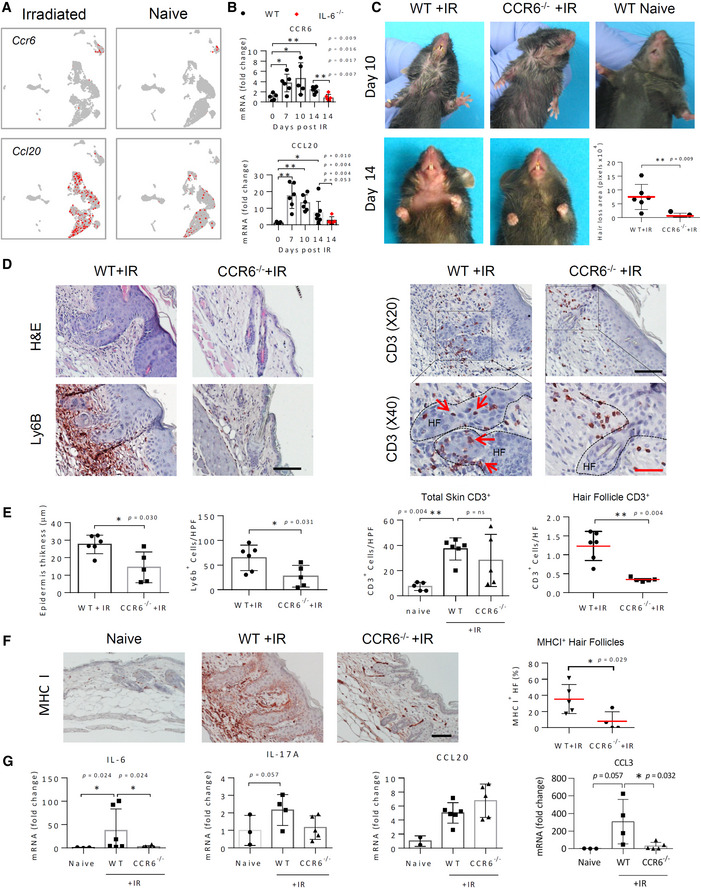
CCR6 deficiency strongly reduces IRIAD in mice AUMAP plots depicting relative expression and localization of *Ccr6* and *Ccl20* mRNAs in irradiated and naïve skin‐derived cell clusters.BQuantification by real‐time qPCR analysis of *Ccr6* and *Ccl20* mRNAs in whole skin of naïve and irradiated (15 Gy) WT and IL‐6^−/−^ mice at indicated times post‐IR, (*n* = 5–7).CPhotographic images showing the development of radiodermatitis and alopecia in irradiated (15 Gy) WT and CCR6^−/−^ mice at indicated times post‐IR. Escalating radiodermatitis, which manifests on day 7–10 as serous exudates on the chin and neck, is visible in both strains and progresses in WT mice to alopecia by day 14 post‐IR but resolves spontaneously in the CCR6^−/−^ mice. Quantification (lower right) of the area of hair loss at 14 days post‐IR, (*n* = 6).DHistochemical staining (H&E) and immunostaining (red) show morphology and infiltration of neutrophils (Ly6b^+^) and T cells (CD3^+^; red arrows) to the skin and to the hair follicle (HF; dashed lines) in WT or CCR6^−/−^ mice 14 days post‐IR. Scale bars, 50 μm (black) and 25 μm (red).EQuantification of epidermal thickness, neutrophils (Ly6b^+^), total T‐cell (CD3^+^) infiltration to the skin, and (F) CD3^+^ cells infiltration to the hair follicle in (D), (*n* = 5–6).FMHC class I immunostaining (red) of skin thin sections prepared at 14 days post‐IR from mice in (C), and quantification (right) of MHC I‐positive hair follicles, (*n* = 4–5). Scale bar, 50 μm.GQuantification by real‐time qPCR analysis of *Il6*, *Il17a*, *Ccl20*, and *Ccl3* mRNAs in the skin of naïve WT and irradiated (15 Gy) WT and CCR6^−/−^ mice at 14 days post‐IR, (*n* = 3–5). Data information: Data represent mean ± SD. *P < 0.05, **P < 0.01 by two‐tailed Student's *t*‐test (E‐Ly6b, H), or by Mann–Whitney test (all other panels). Two independent experiments (See also Appendix Figs S13–S15). Source data are available online for this figure.

Because in the experiment described above, IL17A‐deficient CD3^+^ T cells nevertheless retained their ability to infiltrate into degenerating HFs, thus maintaining a strong correlation with radiodermatitis and alopecia, we instead sought to determine the effect of disrupting the CCL20/CCR6 chemokine signaling that mediates migration of IL‐17‐producing cells (Yamazaki *et al*, 2008) on IRIAD. scRNA‐seq analysis indicated that IR‐induced *Ccr6* mRNA was primarily expressed by cells within the αβT/ILC cluster and that irradiation strikingly upregulated *Ccl20* mRNA, as well as IL‐17‐induced psoriasis‐related chemokines and antimicrobial peptides, including, S100A8, and S100A9 (Furue *et al*, 2020) that localized predominantly within epidermal keratinocyte clusters (Fig 5A and Appendix Fig S13A). CellPhoneDB analysis similarly supported this finding, indicating that potential significant CCL20/CCR6‐mediated cell interactions following irradiation spread throughout epidermal keratinocyte clusters to include IFE‐B, uHF, uHF I/INFU‐B, SG, and IFE‐KII clusters, but not within either dermal fibroblasts or endothelial populations (Appendix Fig S13B). From these observations, we infer that, principally, the epidermal keratinocyte and hair follicle cells direct the irradiation‐induced CCL20‐mediated immune cell infiltration to the skin. Real‐time qPCR analysis confirmed the upregulation of both *Ccl20* and *Ccr6* mRNAs, which reached maximal levels by day 7 post‐IR, prior to maximal IL‐6 upregulation, albeit within the presence of notably relatively high basal IL‐6 levels expressed by BEC and FIB‐III/I cells (Fig 5B and Appendix Fig S7A). Moreover, IL‐6 deficiency in irradiated mice led to significant reductions in both *Ccl20* and *Ccr6* mRNAs levels, thus indicating a strong correlation between IL‐6 and CCL20/CCR6‐mediated αβT/ILC recruitment in IRIAD (Fig 5B).

To determine the role of CCR6/CCL20‐mediated cell recruitment in IRIAD, we subjected CCR6 *knockout* (CCR6^−/−^) mice to irradiation (15G) and followed the development of IRIAD for a period of 2 weeks. To our surprise, at about 7–10 days post‐IR, irradiated CCR6^−/−^ mice presented with a notable inflammatory response, manifest by conspicuous erythema and serous exudations, indistinguishable from that of irradiated WT controls (Fig 5C). However, shortly thereafter, this response spontaneously and abruptly resolved, such that by day 14 post‐IR, the irradiated CCR6^−/−^ mice displayed little hair loss (Fig 5C). Irradiated CCR6^−/−^ mice also displayed dramatically reduced radiodermatitis, with significant reductions in keratinocyte hyperplasia, acanthosis, and neutrophil (Ly6b^+^) infiltration in comparison to irradiated WT controls (Fig 5D and E). Importantly, although overall IR‐induced CD3^+^ T‐cell infiltration into the skin appeared to be maintained in the CCR6^−/−^ mice, close examination revealed that while CCR6 deficiency permitted CD3^+^ T‐cell infiltration to dermal layers, it overwhelmingly excluded infiltration from hair follicles (Fig 5D and E). In contrast, CCR6 deficiency appeared to reduce overall CD4^+^ T‐cell accumulation, including in the dermal layer and hair follicles (Appendix Fig S14). This suggests that CCR6 is important for the overall accumulation of CD4^+^ cells, whereas for the hair follicle, CCR6 is important for the accumulation of CD4^+^ and CD4^−^ T cells.

The apparent exclusion of CD3^+^ T‐cell infiltration from hair follicles of irradiated CCR6^−/−^ mice also suggested that the hair follicles in these mice may retain their immune‐privileged status that is lost in WT mice following irradiation (Paus *et al*, 2005; Ito & Tokura, 2014). Indeed, MHC I immunostaining demonstrated a striking upregulation in the dermis and hair follicles of irradiated WT mice, consistent with loss of immune privilege status (Paus *et al*, 2005), that was significantly attenuated in CCR6^−/−^ mice, being restricted mainly to areas outside of the hair follicles (Fig 5F). Irradiated CCR6^−/−^ mice also displayed significant reductions in expression of IL‐6, IL‐17, and CCL3 mRNAs, suggesting a critical cross talk between infiltration and activation of IL‐17‐producing CCR6^+^ T/ILC cells, with both IL‐6 upregulation and neutrophil infiltration in IRIAD (Fig 5G). Nevertheless, despite reductions in cytokine signaling and radiodermatitis in CCR6^−/−^ mice, *Ccl20* mRNA upregulation remained elevated in comparison to naive WT controls, indicating the persistence of pro‐inflammatory signals in these mice (Fig 5G). Notably, CCR6 deficiency also substantially diminished the IR‐induced p16^INK4a^ upregulation and the accumulation of senescence‐associated lipofuscin (Appendix Fig S15), suggesting a supporting, proximity‐dependent relationship between infiltrating IL‐17‐expressing CCR6^+^ T cells and senescent epidermal and dermal skin cells. These findings demonstrate that CCR6/CCL20‐mediated immune cell migration promotes IRIAD together with multiple IL‐6‐ and IL‐1R‐mediated cellular responses, including keratinocyte hyperplasia, acanthosis, and senescence.

### Cyclosporine A suppresses IRIAD in mice

**Figure 6:**
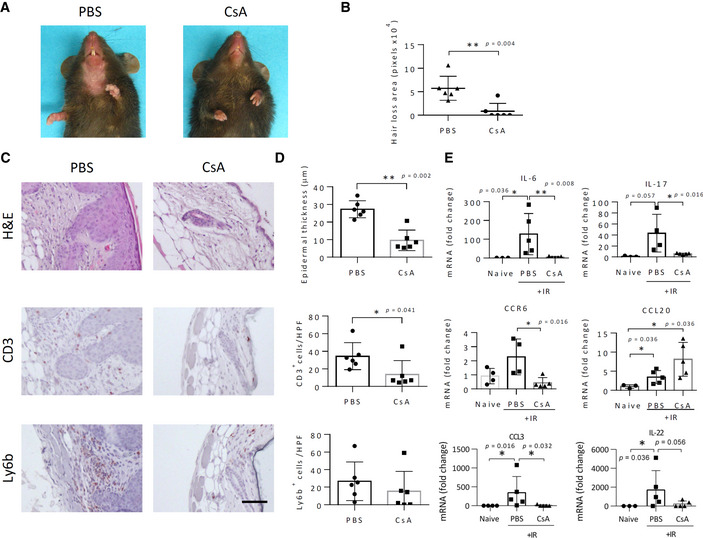
Cyclosporine A treatment prevents IR‐induced alopecia and radiodermatitis APhotographic images showing hair loss in control (PBS) and cyclosporine (CsA) treated WT mice at 14 days post‐IR (15 Gy).BQuantification of the area of ventral hair loss in mice in (A), (*n* = 6).CHistochemical (H&E) and immunostaining (red) show T cell (CD3^+^) and neutrophil (Ly6b^+^) infiltration in the skin of mice in (A). Scale bar, 100 μm. (Images of CD3 and Ly6b immunostaining from CsA‐treated mice represent separate analyses performed on serial thin sections.)DQuantification of epidermal thickness, and T‐cell (CD3^+^) and neutrophil (Ly6b^+^) infiltration (red staining) in skin thin sections from (C), (*n* = 6).EQuantification by real‐time qPCR analysis of *Il6*, *Il17a*, *II22*, *Ccl3*, *Ccr6*, and *Ccl20* mRNAs in whole skin of naïve and irradiated (15 Gy) control (PBS) and CSA‐treated WT mice at 14 days post‐IR, (*n* = 5). Data information: Data represent mean ± SD. *P < 0.05, **P < 0.01 by two‐tailed Mann–Whitney test; two independent experiments. Source data are available online for this figure.

To determine whether suppression of T‐cell migration and activation can serve as a practical therapeutic tool for preventing IRIAD, we administered the T‐cell suppressant, cyclosporine A (CSA; Flanagan *et al*, 1991; Wang *et al*, 2015) to irradiated WT mice and followed IRIAD development as before. As shown in Fig 6A–D, CSA administration to the irradiated mice strongly reduced alopecia and most histological parameters of IRIAD, including hair follicle degeneration, keratinocyte hyperplasia, and acanthosis. CSA treatment also reduced CD3^+^ T‐cell infiltration by about 2.5‐fold, thus confirming its effect as a T‐cell suppressant, and marginally reduced neutrophil infiltration but without statistical significance (Fig 6D). In line with its effect as an immune suppressant, CSA treatment also strongly reduced IR‐induced upregulation of IL‐6, IL‐17, IL‐22, CCL3, and CCR6, but not that of CCL20 (Fig 6E), thus largely recapitulating the effect of CCR6 ablation.

Taken together, these findings demonstrate a crucial cross talk between senescence‐associated IL‐6 and IL‐1 upregulation and CCL20/CCR6‐mediated recruitment of IL‐17 producing αβT/ILC cells that drive IRIAD.

## Discussion

While the sensitivity of skin and hair follicles to injury in human radiotherapy patients has long been recognized, the driving mechanisms of IRIAD remain unclear, thus leaving a largely unanswered therapeutic need (Chase, 1954; Malkinson & Keane, 1981; Hymes *et al*, 2006). In the present study, we have combined scRNA‐seq analysis to an irradiated murine model together with genetically deficient mice in order to elucidate molecular and cellular mechanisms underlying IRIAD. Cellular senescence and upregulation of the SASP, including IL‐6 and IL‐1, are expected outcomes of ionizing radiation‐induced DNA damage, as others and we have previously reported (Marmary *et al*, 2016; He & Sharpless, 2017), and, as observed here, are inherently associated with IRIAD. Moreover, our findings reveal a pivotal, senescence‐associated cross talk between IR‐induced cytokines/chemokines, IL‐6, IL‐1, and CCL20, produced by keratinocytes and fibroblasts, and chemokine‐mediated infiltration of IL‐17‐producing Ccr6^+^ T cells that, in an apparent feedback loop, drive the hallmark features of radiodermatitis and transient hair loss (Appendix Fig S16).

scRNA‐seq and bioinformatics analyses have provided an opportunity to understand in depth the complexity of the cellular and molecular drivers of IRIAD. Thus, dermal fibroblasts (FIB‐III/I) and epidermal keratinocytes (IFE‐B) are identified as the primary sources of IR‐induced IL‐6. In contrast, while upregulation of IL‐1α was largely found within clusters of differentiated keratinocytes (IFE‐KII), sebaceous gland cells, and IFE‐B cells, upregulated IL‐1β expression was observed exclusively within innate dendritic cells, Langerhans cells, and granulocytes. Moreover, despite the relatively small numbers of IL‐6‐ and IL‐1‐producing cells, bioinformatics analysis and molecular blockade experiments indicate that cytokine signaling spreads throughout irradiated dermal and epidermal layers, apparently via paracrine mechanisms, which in the case of IL‐1β signaling is likely assisted by migration of innate immune cells.

Our findings also provide strong evidence that IL‐6 and IL‐1 signaling critically underlie both the frank IR‐induced inflammatory response and keratinocyte hyperplasia, since the deficiency or blockade of signaling by either cytokine strongly diminished both features. Accordingly, populations of dividing epithelial cells in irradiated skin concentrate primarily within the basal keratinocyte cluster, IFE‐B, converging with IPA‐defined IL‐6 signaling. This is in agreement with previous studies showing that IL‐6 *per se* promotes human keratinocyte proliferation *in vitro* and that ectopic IL‐6 expression in rat skin via naked DNA transfection induces keratinocyte proliferation, as well as lymphocytic infiltration and erythema (Grossman *et al*, 1989; Yoshizaki *et al*, 1990; Sawamura *et al*, 1998). At the same time, in many irradiated skin cell clusters, including IFE‐B cells, IL‐6 signaling also overlaps with increased senescence pathways. Although counterintuitive, the notion that senescence may be a key driver of keratinocyte hyperplasia in irradiated skin aligns well with observations showing that senescent cells and the SASP, including IL‐6, play essential roles in optimizing skin wound healing (Gallucci *et al*, 2000; Demaria *et al*, 2014; Ritschka *et al*, 2017). Moreover, oncogene‐induced cellular senescence by conditional ectopic H‐Ras^G12V^ transgene expression in epidermal keratinocytes, involving SASP upregulation, including IL‐6 and IL‐1β, also reportedly produces marked keratinocyte hyperplasia and acanthosis, together with age‐related inflammation (Golomb *et al*, 2015). However, our findings also indicate that IL‐6 signaling appears to be absent in about half of the cycling, non‐senescent IFE‐B cells, thus suggesting that factors in irradiated skin other than IL‐6 may also promote basal keratinocyte hyperplasia. Th17‐expressed IL‐22, which mediates IL‐23‐induced acanthosis and dermal inflammation in psoriasis (Zheng *et al*, 2007; Nograles *et al*, 2008), is also expressed by cells within the αβT/ILC cluster and may also contribute to IR‐induced keratinocyte hyperplasia. In this and other respects, IRIAD shows a high degree of similarity with psoriasis, including the Th17 cytokine‐related inflammatory response, as well as the simultaneous presence of keratinocyte hyperplasia together with the presence of cellular senescence in keratinocytes associated with high IGFBP2 expression and p16INK4a upregulation (Mercurio *et al*, 2020).

According to the present study's findings, IL‐6 signaling is crucial for IL‐17 upregulation and for CD3^+^ T‐cell infiltration to the HF, and, vis‐a‐versa; IR‐induced IL‐6 upregulation is strongly dependent upon CCR6^+^ cell infiltration and IL‐17 upregulation. This strongly suggests that a progressive feedback loop involving an IR‐induced IL‐6/IL‐1/CCR6‐CCL20/IL‐17 cytokine and chemokine cascade mediates the collapse of the hair follicle immune privilege status and T/ILC cell infiltration, leading to hair follicle degeneration (Appendix Fig S16). This conclusion is in agreement with previous observations showing that IL‐6 *trans*‐signaling and TGF‐β are critical for the development and activation of Th17 cells (Dominitzki *et al*, 2007; Korn *et al*, 2009; Jones *et al*, 2010), and that neutralization of IL‐6 signaling functioning downstream of IL‐17A efficiently reduces the psoriasis‐like pathogenesis, including infiltration of effector T cells, formation of neutrophil microabscesses, and acanthosis in the skin of IL‐17A‐overexpressing mice (Croxford *et al*, 2014). Similarly, IL‐1β in combination with IL‐23 can induce IL‐17 and IL‐22 expression by γδT cells (Sutton *et al*, 2009). Conversely, induction of IL‐1 and IL‐6 in various cell types and upregulation of ICAM‐1 and HLA‐DR expression in keratinocytes are among some of the known pleiotropic activities of IL‐17 (Fossiez *et al*, 1998; Jovanovic *et al*, 1998; Albanesi *et al*, 1999). Thus, although the hair follicle epithelium displays features of relative immune privilege, characterized by very low expression of MHC Ia and suppressed MHC II‐dependent antigen presentation (Paus *et al*, 2005), it is plausible that IL‐17 expression, perhaps from infiltrating CD3^+^ T cells, may underlie the MHC I upregulation and the apparent collapse of immune privilege in the hair follicle that accompany IR‐induced hair loss. The close association of IL‐6‐dependent neutrophil infiltration with IRIAD, which is mediated in part by CCL3, suggests that neutrophils may also contribute to IRIAD, perhaps by the generation of local oxidative stress, and also by acting as a source of sIL‐6R for IL‐6 *trans*‐signaling (Chalaris *et al*, 2007).

Although IL‐17, IL‐22, and CCR6 upregulation are commonly considered as signature markers of Th17‐mediated skin pathologies (Di Cesare *et al*, 2009), these markers are also expressed by γδT cells, which normally reside in the skin and other mucosal tissues and act as immune sentinel cells (Mabuchi *et al*, 2011; Nielsen *et al*, 2017), as well as in subsets of CCR6^+^ type 3 innate lymphoid (ILC3) cells (Dumoutier *et al*, 2011; Spits *et al*, 2013; Salimi & Ogg, 2014). In the present study, we find that while IL‐17 was upregulated in nearly all cells of the irradiated αβT/ILC cluster and in a majority of γδT cells, scRNA‐seq analysis detected CCR6 expression exclusively within a subset of the αβT/ILC cell population. However, as previously reported, γδT cells, which constitutively express CCR6 that is necessary for their recruitment to the inflamed skin (Campbell *et al*, 2017; McKenzie *et al*, 2017), are also reportedly important effectors of radiodermatitis in mice (Liao *et al*, 2017). This suggests that our findings may represent an underestimation of CCR6^+^ cell populations, perhaps due to limitations of the scRNA‐seq technology employed here. Thus, while the present findings clearly establish roles of IL‐17 and CCR6, and strongly point to an effector role of Th17 cells in IRIAD, they cannot be considered to unequivocally rule out contributions by γδT cells and ILCs.

As radiotherapy remains a principle means for the treatment of a wide range of cancers, including breast cancer, head and neck cancers, and cancers of the brain, we sought to elucidate the driving mechanism(s) of IRIAD in order to identify therapeutic targets of the acute and chronic side effects associated with irradiation injury to the skin. Although these invaliding complications have long been recognized in patients, surprisingly, little is known about their underlying mechanisms. This is due in part to the complexity of the interaction between the many cell types, and also due to structural changes and the extreme sensitivity of the inflamed skin following radiotherapy, which greatly complicates surgical procedures and wound healing (Gieringer *et al*, 2011; Haubner *et al*, 2012). Hence, for ethical considerations, skin biopsies from irradiated areas in patients are difficult to justify and thus have not been made available in order to validate the observations of this animal model. This also reflects the paucity of therapeutic prophylaxis for IRIAD. Indeed, IRIAD remains a substantial unmet clinical challenge despite the various tested approaches for its prevention or treatment, which include palliative washing with mild soaps, the topical application of radioemulsions or corticosteroids, the administration of Toll‐like receptor 5 agonists, as well as treatments for the prevention of alopecia by scalp cooling, or the topical application of the nitroxide radioprotector, Tempol (Shah *et al*, 2000; Metz *et al*, 2004; Hymes *et al*, 2006; Burdelya *et al*, 2012).

While there are considerable differences between the skin and hair of mice and humans, including structural, cellular, and immune components (Randall *et al*, 2003; Pasparakis *et al*, 2014), the definitive roles of cytokine and chemokine signaling by IL‐6, IL‐1, IL‐17, and CCR6‐CCL20 in IRIAD demonstrated here present multiple opportunities for the development of targeted inhibitors to ameliorate IRIAD in the clinic (Xing *et al*, 2014). Several inhibitors of IL‐6 and IL‐1 signaling are currently approved or in development for clinical use (Schett *et al*, 2016; Garbers *et al*, 2018), as are potential pharmacological agents that target the CCR6‐CCL20 axis (Ranasinghe & Eri, 2018). Importantly, the findings of this study suggest that targeting small molecular weight inhibitors to epidermal keratinocyte layers could have profound therapeutic effects on the development of radiodermatitis. Indeed, the striking response in irradiated mice to topical application of the JAK kinase inhibitors, ruxolitinib and tofacitinib, and cyclosporine suggests that these agents should be of immediate consideration for clinical evaluation in patients with radiodermatitis. Moreover, the apparent mechanistic similarities of radiodermatitis and psoriasis found here in comparison to other studies suggest that treatments found effective for one condition may well be effective for the other as well. This will naturally also depend on safety and efficacy considerations, with particular reference to potential interference with radiotherapy and chemotherapy protocols aimed at treating the patient's cancer.

## Materials and Methods

### Study approval

The Joint Ethics Committee (IACUC) of the Hebrew University and Hadassah Medical Center approved the study protocol for animal welfare. The Hebrew University is an accredited institute of the American Association for the Accreditation of Laboratory Animal Care (AAALAC) International.

### Mice

Mice (C57BL/6; Harlan Laboratories Ltd., Jerusalem), IL‐6^−/−^ (B6;129S2‐Il6^*tm1Kopf*^/J, C57BL/6; The Jackson Laboratory; Kopf *et al*, 1994), sgp130Fc (C57BL/6) mice (Rabe *et al*, 2008), IL‐1R^−/−^ (C57BL/6) mice (Glaccum *et al*, 1997), CCR6^−/−^ (C57BL/6) mice (Hedrick *et al*, 2009), and IL‐17^−/−^ mice (Nakae *et al*, 2002) were maintained in an animal facility under SPF conditions, at a temperature of ~23°C in a 12‐h light–dark cycle. Mice received sterile commercial rodent chow and water *ad libitum*. Procedures and maintenance were performed in accordance with the Institutional Animal Care and Use Committee‐approved animal treatment protocols (license number OPRR‐A01‐5011).

Female mice, 6‐ to 7‐week old, were irradiated to the head and neck while immobilized in Perspex tubular jigs without anesthesia. The 6MV energy X‐Ray radiation was delivered by a Varian linear accelerator, Clinac 2100EX model (FSD = 100 cm, field size = 7 × 40 cm), in a single fraction dose of 15 Gy, unless indicated otherwise (Marmary *et al*, 2016). Antibody‐mediated IL‐6 inhibition experiments were performed using neutralizing monoclonal anti‐mouse IL‐6 antibody (MAB406; R&D Systems Inc.; 200 mg, i.p.), or a monoclonal IgG1 isotype control antibody (MAB005; R&D Systems Inc.; 200 mg, i.p.) administered 2 h prior to irradiation and on day 7 post‐IR. Cyclosporine (Sandimmune; NOVARTIS; 22 mg/kg, i.p.) was administered daily for 14 days beginning immediately following irradiation.

For topical treatment studies, mice were anesthetized (ketamine/xylazine) 4–6 days prior to irradiation, and chest and neck fur was sheared. Mice were treated immediately following irradiation and then once daily for 14 days with vehicle (10% DMSO in Aquaphor) or vehicle containing JAK inhibitors, ruxolitinib (INCB18424), and tofacitinib (CP‐690550; AbMole BioScience), initially dissolved in DMSO and then mixed 1:10 in Aquasum (Aquaphor), to achieve 0.5% JAK inhibitor ointment.

Hair loss, which typically began approximately 2 weeks post‐IR and reached maximal levels by about 3 weeks post‐IR, was recorded by digital photography at indicated times post‐IR on anesthetized (ketamine/xylazine) mice using a standardized camera placement and magnification. In order to reduce the number of experimentally treated animals exposed to irradiation, hair loss and inflammatory markers were quantified either at 21 days post‐IR or, alternatively, at 14 days post‐IR prior to taking skin samples for molecular and histochemical analyses. The area of hair loss was quantified in square pixels (pixels) using ImageJ Imaging software (NIH).

For the collection of tissue specimens, full‐thickness skin biopsies were excised at indicated time points prior to sacrifice from the anterior surface (neck and upper chest) of anesthetized (ketamine/xylazine) mice and were either snap frozen in liquid nitrogen for RNA extraction, snap frozen in OCT for immunostaining, or fixed in 4% buffered formaldehyde. Data evaluation from animal experiments was performed by the researchers in non‐blinded conditions.

### 3′ single‐cell RNA sequencing

Cells for scRNA‐seq analysis were prepared from whole skin samples removed from shaved naive or irradiated mice essentially as described (Guerrero‐Juarez *et al*, 2019). Briefly, skin samples were treated by Dispase 2 (Sigma‐Aldrich) for 60 min at 37°C and separated into dermal and epidermal layers. The separated skin layers were minced into small pieces, pooled, and treated by shaking at 37°C for 100 min in a solution of Liberase™ Research Grade (Roche)/DNase (Roche) in PBS containing 2% FCS (WS) to disperse the cells. EDTA was added to 7 mM during the final 10 min incubation. Post incubation, single‐cell suspensions were diluted with an equal volume of WS containing 2× cOmplete™ Protease Inhibitor Cocktail (Sigma‐Aldrich) and strained through a 70‐μM filter to remove large tissue debris. Cell suspensions derived from skin samples of four mice per treatment group were pooled and dead cells were removed using the Dead Cell Removal Kit (MACS) as per the manufacturer's directions and repeated one time to achieve a suspension of ~90% viable cells. Live cells were suspended in PBS containing 10% FCS and used for cDNA library construction using Chromium Single Cell 3′ GEM, Library & Gel Bead Kit v3 (10× Genomics, 6230 Stoneridge Mall Road Pleasanton, CA 94588 USA) according to the manufacturer's instructions (https://www.10xgenomics.com/support/single‐cell‐gene‐expression/documentation/steps/library‐prep). Sequencing was performed using the Illumina Nextseq500 platforms with the following sequencing conditions: 26 bp (Read1) and 58 bp (Read2). Library preparation, quality control, and sequencing were performed at the Core Research Facility, The Faculty of Medicine – Ein Kerem, The Hebrew University of Jerusalem, Israel.

### Single‐cell RNA‐Seq data processing and integration

Raw reads of each sample were processed using the “count” command of the Cell Ranger software, v2.0.2, aligning the reads to the mouse mm10 (GRCm38) genome version. The generated report was used for assessing the quality of the samples in terms of cell numbers (5,450, 4,177), the average reads per cell (32,761, 105,484), the fraction of reads in cells (95.8, 96.6%), and alignment rate and saturation (61.7, 65.4%) for naive and irradiated samples, respectively. The datasets were filtered further to retain only high‐quality cells with more than 200 expressed genes and less than 5% of mitochondrial RNA transcripts. Genes expressed in less than three cells were removed. Finally, 5,135 naive and 3,670 irradiated cells were retained for analysis using the package Seurat 3.0.2 and Seurat 4.0.4 (Satija *et al*, 2015; Butler *et al*, 2018; Stuart *et al*, 2019) on R3.6.3 and R4.0, respectively. Datasets were normalized using “LogNormalize”, a global‐scaling normalization method. From each sample, 2,000 highly variable genes were selected for the downstream analysis. Datasets were then integrated using the Seurat FindIntegrationAnchors function, followed by the function IntegrateData with 1 to 40 dimensions. The integrated data were scaled, centered, subjected to dimension reduction using PCA1 to PCA40 and clustered at 0.6 resolution using PCA1 to PCA40, and visualized by Uniform Manifold Approximation and Projection (UMAP).

Seurat FindMarkers function using the non‐parametric Wilcoxon rank‐sum test was used for the identification of differentially expressed genes between clusters, both within and between the experimental groups. The log2 fold change (FC) threshold and the minimum fraction of gene detection in either of the two groups were set to zero in order to enable the ranking of genes required for the Gene Set Enrichment Analysis GSEA. Figures to visualize the clusters, as well as the marker expression in the low‐dimensional space (UMAP), were generated by Seurat visualization functions. For simultaneous visualization of co‐expressed markers, the blend argument was applied in the FeaturePlot Seurat function (Hao *et al*, 2021). Dot plots were generated using Seurat DotPlot function. Customization of Seurat's function was performed using ggplot2's themes (Wickham, 2016).

### Cell type identification and functional enrichment analysis

Cell type identities of clusters (0, 1, 2, 3, 4, 5, 8, 9, 10, 11, 14) were determined according to gene expression data of naïve cells using the non‐cutoff‐dependent Gene Set Enrichment Analysis (GSEA) tool (Subramanian *et al*, 2005) utilizing previously published gene set signatures markers for dermal (Guerrero‐Juarez *et al*, 2019) and epidermal skin cell types (Joost *et al*, 2016). For each cluster, the expressed genes in naïve cells were ranked by their expression log2FC versus the general expression of all other cells. Immune clusters and some endothelial cell populations clusters (7, 10, 12, 13, 15, 16, 17, 18, 19, 20, 21) were identified by comparisons within the Immunological Genome Project (ImmGen; https://www.immgen.org/; Heng *et al*, 2008).

### Ingenuity pathway analysis

Pathway enrichment analysis of the significantly differentially expressed genes between naïve and irradiated cells in each cluster was carried out using the Ingenuity Pathway Analysis (IPA^®^; QIAGEN Inc., https://digitalinsights.qiagen.com/products‐overview/discovery‐insights‐portfolio/content‐exploration‐and‐databases/qiagen‐ipa/). Genes with adjusted *P*‐value < 0.05 and expressed in ≥ 25% of cells in at least one of the compared groups were considered as significantly differentially expressed. IPA *z*‐scores > 2 or IPA *z*‐score < −2 are confidently predicted to be up/down‐regulated.

### CellPhoneDB ligand–receptor interaction analysis

ScRNAseq expression matrix gene IDs were converted from mouse to human using the OMA ontology browser (https://omabrowser.org/oma/home/) data. Genes were used for further analysis only if their mouse to human gene pairing was a 1:1 match. Converted expression matrix per sample together with cluster metadata as generated in the scRNA‐seq analysis was subjected to CellPhoneDB analysis using default parameters. For selected ligand–receptor interactions, CellPhoneDB analysis outputs of *P*‐values and means of the average expression level of interacting molecule 1 in cluster 1 (*Y*‐axis) and interacting molecule 2 in cluster 2 (*X*‐axis) were used to generate a separate cluster interactions heat map. Cluster interaction heat maps were generated using the R package corrplot (https://cran.r‐project.org/web/packages/corrplot/vignettes/corrplot‐intro.html). Means of the average expression level of interacting molecule 1 in cluster 1 (*Y*‐axis) and interacting molecule 2 in cluster 2 (*X*‐axis) with *P* < 0.05 were indicated by the color and size of each data point.

### Antibodies

Antibodies used for immunostaining (IHC) and western blot (WB) analyses were as follows: anti‐CD3 (MCA1477, 1:300; Bio‐Rad), anti‐Ly6B (MCA771G, 1:3,000; Bio‐Rad), anti‐CD4 (ab183685, 1:1,000; Abcam), anti‐MHC class I (ab15681, 1:100; Abcam), anti‐Ki67 (#9106, 1:100; NeoMarker SP6), anti‐phospho‐STAT3 (pTyr‐705) for IHC (#9145, 1:200; Cell Signaling Technology), anti‐phospho‐STAT3 (pTyr‐705) for WB (sc‐8,059, 1:200; Santa Cruz Biotechnology), anti‐STAT3 (sc‐8019, 1:200; Santa Cruz Biotechnology), anti‐phospho‐ERK1/2 (M8159, 1:4,000; Sigma), PLAUR/uPAR antibody (orb13650, 1:300; Biorbyt Ltd.), anti‐p16^INK4A^ (ab54210, 1:100; Abcam), and β‐actin antibody (A5316, 1:10,000; Sigma). Immunostainings and western blots were developed using anti‐mouse HRP polymer or anti‐Rabbit HRP polymer (Dako, Denmark). Immunohistochemical staining for lipofuscin in senescent cells was performed using SenTraGor™ (Arriani Pharmaceuticals, Athens, Greece) according to the manufacturer's instructions and developed with an anti‐biotin antibody (ab234284, 0.5 μg/ml; Abcam).

### Immunohistochemistry

Thin sections (5 μm thick) of skin biopsies fixed in 4% buffered formaldehyde, followed by 80% ethanol and embedded in paraffin blocks were stained with H&E by standard procedures for histological analyses or stained overnight with anti‐mouse antibodies at 4°C in a humidified chamber. Epidermal thickening was measured on photomicrographs of H&E stained thin sections using CellSens Entry, Olympus software.

### Western blot analysis

Protein extracts (50 μg) prepared from flash‐frozen tissue samples by homogenization were separated by polyacrylamide gel electrophoresis and subjected to western blot analysis as described previously (Marmary *et al*, 2016) and probed overnight with anti‐mouse antibodies.

### RNA

RNA for real‐time qPCR analysis was extracted from snap‐frozen tissue specimens using Rneasy Fibrous Tissue Mini Kit (QIAGEN). Reverse transcription of total RNA was performed using the qScriptTM cDNA Synthesis Kit (#95047) and Quantitative PCR (qPCR) of mRNA was performed using PerfeCTa SYBR Green FastMix ROX (#95073; Quanta Biosciences Inc., Gaithersburg, MD, USA). Target mRNAs were normalized to *Ndufb9*. qPCR assays were performed in triplicate using an AB 7900 HT fast Real‐Time PCR system (Applied Biosystems, Foster City, CA, USA) or CFX384 TM Real‐Time System with C1000 Touch Thermal Cycle (Bio‐Rad, Hercules, CA, USA).

Primers used for qPCR:

**Table:** 

Gene	Forward 5′ → 3′	Reverse 5′ → 3′
IL‐6	ATACCACTCCCAACAGACCTGTCT	CAGAATTGCCATTGCACAACTC
IL‐17	GGAAAGCTGGACCACCACA	CACACCCACCAGCATCTTCTC
CCL20	TTGCTTTGGCATGGGTACTG	TCGGCCATCTGTCTTGTGAA
CCL3	TTCTCTGTACCATGACACTCTGC	CGTGGAATCTTCCGGCTGTAG
CCR6	ATGCGGTCAACTTTAACTGTGG	CCCGGAAAGATTTGGTTGCCT
TNF‐α	CTGTAGCCCACGTCGTAGCAA	CTGGCACCACTAGTTGGTTGTCT
IL‐22	TCCGAGGAGTCAGTGCTAAA	AGAACGTCTTCCAGGGTGAA
IL‐23	AATAATGTGCCCCGTATCCAGT	GCTCCCCTTTGAAGATGTCAG
CCR1	CAGAGCCCTCCTACAACACAG	CCAGGGAGTACAGGGGAGT
CCR3	TCAACTTGGCAATTTCTGACCT	CAGCATGGACGATAGCCAGG
CCL2	CTTCTGGGCCTGCTGTTCA	CCAGCCTACTCATTGGGATCA
CCR5	TTTTCAAGGGTCAGTTCCGAC	GGAAGACCATCATGTTACCCAC
IL‐2	CCCAAGCAGGCCACAGAATTGAAA	AGTCAAATCCAGAACATGCCGCAG
TGF‐β	TGACGTCACTGGAGTTGTACGG	GGTTCATGTCATGGATGGTGC
IL‐4	AGATGGATGTGCCAAACGTCCTCA	AATATGCGAAGCACCTTGGAAGCC
CCR2	ATCCACGGCATACTATCAACATC	CAAGGCTCACCATCATCGTAG
CCL5	GTGCCCACGTCAAGGAGTAT	CTCTGGGTTGGCACACACTT
IFN‐γ	AGAGGATGGTTTGCATCTGGGTCA	ACAACGCTATGCAGCTTGTTCGTG
Ndufb9	CAGCCGTATATCTTCCCAGACT	CTCAGAGGGATGCCAGTAATCTA

### Statistics

Data were evaluated for significance by two‐tailed Student's *t*‐test, Mann–Whitney test, or two‐way ANOVA, as indicated in the figure legends, with *P* ≤ 0.05 considered significant for all analyses. Calculations were performed using GraphPad Prism 6.02 software (GraphPad Software, Inc., San Diego, CA).

## Author contributions

**Mor Paldor:** Conceptualization; investigation; writing – original draft. **Orr Levkovitch‐Siany:** Investigation; methodology. **Dana Eidelshtein:** Investigation. **Claes D Enk:** Conceptualization; writing – review and editing. **Yitzhak Marmary:** Conceptualization; writing – review and editing. **Revital Adar:** Investigation. **Sharona Elgavish:** Formal analysis; writing – review and editing. **Yuval Nevo:** Data curation; formal analysis. **Hadar Benyamini:** Formal analysis. **Inbar Plaschkes:** Formal analysis. **Shiri Klein:** Conceptualization. **Alex Mali:** Formal analysis. **Stefan Rose‐John:** Conceptualization; resources; writing – review and editing. **Amnon Peled:** Conceptualization; resources; writing – review and editing. **Eithan Galun:** Conceptualization; supervision; funding acquisition; writing – review and editing. **Jonathan H Axelrod:** Conceptualization; formal analysis; supervision; funding acquisition; investigation; writing – original draft; project administration; writing – review and editing.

In addition to the CRediT author contributions listed above, the contributions in detail are:

JHA and EG conceived and supervised the study. JHA, MP, EG, AP, CDE, and YM designed the experiments. MP and OL‐S conducted the experiments. MP, OL‐S, RA, SK, and DE acquired data. MP, JHA, EG, SR‐J, AP, DE, SE, YN, HB, IP, SK, and AM analyzed data. AP and SR‐J provided reagents. MP and JHA assembled the data and wrote the manuscript. All authors discussed the results and edited the manuscript.

## Disclosure and competing interests statement

SR‐J has acted as a consultant and speaker for AbbVie, Chugai, Genentech Roche, Pfizer, and Sanofi. He also declares that he is an inventor on patents owned by CONARIS Research Institute, which develops the sgp130Fc protein Olamkicept together with the companies Ferring and I‐Mab. SR‐J has stock ownership in CONARIS. JHA, EG, SR‐J, and YM are listed inventors of pending intellectual property based on the findings of this study.

## Supporting information



Appendix

Expanded View Figures PDF

Table EV1

Source Data for Expanded View

Source Data for Figure 2

Source Data for Figure 3

Source Data for Figure 4

Source Data for Figure 5

Source Data for Figure 6

## Data Availability

The datasets and computer code produced in this study are available in the following databases: scRNA‐Seq: GEO GSE201447 (https://www.ncbi.nlm.nih.gov/geo/query/acc.cgi?acc=GSE201447).
