# Supplementary material for: Single‐cell transcriptomics reveals a senescence‐associated IL‐6/CCR6 axis driving radiodermatitis
Source: EMBO Mol Med. 2022 Jul 4;14(8):e15653. doi: 10.15252/emmm.202115653 (PMC9358397; doi:10.15252/emmm.202115653)
Supplement: Supplementary file 1 — Appendix [file EMMM-14-e15653-s005.pdf]

Appendix Table of Contents

Appendix Table S1: Cell Number per Cluster.

Appendix Figure S1: Characterization of IRIAD in a mouse model.

Appendix Figure S2: GSEA Identification of major naïve integrated cell clusters.

Appendix Figure S3: Cell cluster identity determined through ImmGen analysis.

Appendix Figure S4: Marker genes expressed in $\alpha\beta$T/ILC Cluster 7.

Appendix Figure S5: scRNA-seq identifies cycling cells in mouse skin.

Appendix Figure S6: CellPhoneDB analysis shows the effect of irradiation on numbers of potential ligand-receptor interactions in mouse skin derived cell clusters.

Appendix Figure S7: Irradiation increases potential IL-6 mediated ligand-receptor interactions between skin cell clusters.

Appendix Figure S8: IR-induced IFN-γ mRNA upregulation in the skin is not affected by IL-6 deficiency.

Appendix Figure S9: Partial overlap of IL-6 signaling with cell cycle scoring in irradiated epidermal cells.

Appendix Figure S10: Irradiation increases IL-1 mediated ligand-receptor interaction potential between skin cell clusters.

Appendix Figure S11: IL-1R deficiency reduces irradiation-induced p16INK4a upregulation and hair depigmentation in mice.

Appendix Figure S12: Irradiation increases IL-22 (Il22) mRNA expression and IL-17 signaling potential between skin cell clusters.

Appendix Figure S13: Irradiation-induced alarmin expression and CCL20-CCR6 interaction potential concentrate within epidermal skin cell clusters.

Appendix Figure S14: Ccr6 deficiency reduces irradiation-induced $CD4^+$ T cell infiltration to the dermis and hair follicles in mice.

Appendix Figure S15: Ccr6 deficiency reduces irradiated-induced cellular senescence-associated markers in mouse skin.

Appendix Figure S16: A schematic model of senescence-associated IL-6/IL-1/CCR6 axis in irradiation-induced dermatitis and hair loss.

Appendix Table S1: Cell Number per Cluster

		Naïve		Irradiated	
Cluster No.	Cell Type	Cell #	%	Cell #	%
0	IFE BII	1154	22%	323	9%
1	IFE DI	1017	20%	371	10%
2	IFE B	224	4%	660	18%
3	IFE BI	512	10%	235	6%
4	IFE KII	369	7%	275	8%
5	FIB III/I	460	9%	140	4%
6	SG	67	1%	346	9%
7	αβTC/ILC	46	1%	365	10%
8	uHF I/INFU B	164	3%	238	7%
9	uHF II/V	164	3%	137	4%
10	FIB	258	5%	41	1%
11	OB/IB	178	3%	39	1%
12	Dendritic	55	1%	136	4%
13	DN	82	2%	85	2%
14	IFE DI/KII	110	2%	30	1%
15	γδTC	70	1%	55	2%
16	LEC	86	2%	34	1%
17	BEC	73	1%	39	1%
18	Langerhans	30	1%	55	2%
19	EC	16	0.3%	12	0.3%
20	NK/ILCs	0	0.0%	27	1%
Total		5135	100%	3643	100%

Appendix Figure S1

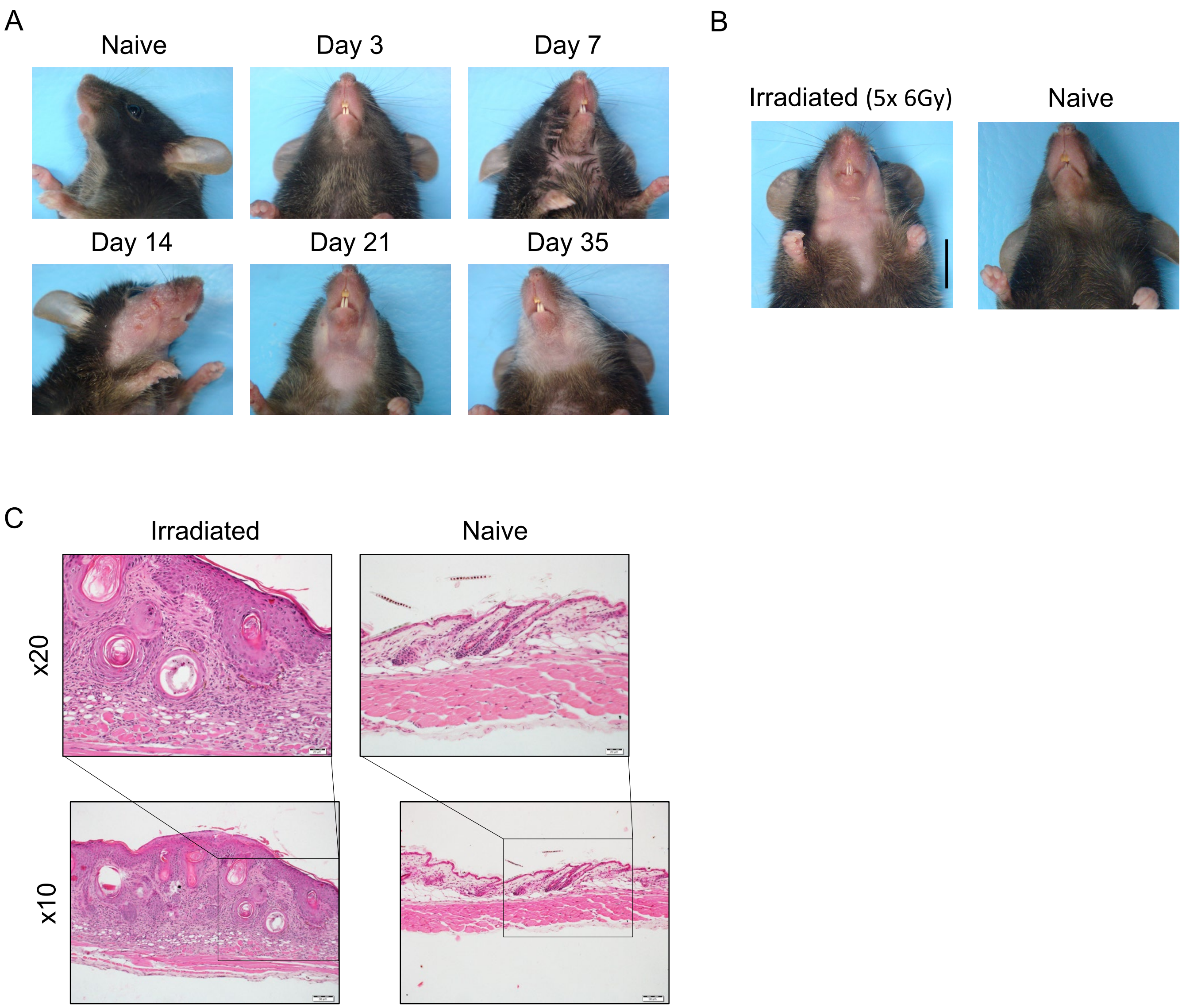

Appendix Figure S1 (Related to Figure 1). Characterization of IRIAD in a mouse model.

A. Photographic images showing kinetics of radiodermatitis, alopecia, and hair depigmentation in side and ventral views of wild type mice irradiated (15 Gy) to the head and neck. Times (in days) post-IR are indicated. Serous exudates appear under the chin at 8 days post-IR with desquamation beginning at about 14 days post-IR. Hair growth reappears between 3-8 weeks post-IR showing extensive hair color loss.

B. Photographic images showing ventral alopecia at day 21 post-IR in wild type mice irradiated (30Gy) to the head and neck in fractionated doses (5x 6Gy). Scale bar, 1 cm.

C. Histochemical (H&E) staining of thin sections showing skin morphology in naïve mice and irradiated (15Gy) mice at 14 days post-IR. Histological hall marks of radiodermatitis, including inflammation, acanthosis, and hair follicle degeneration are evident in irradiated skin specimens. Scale bars, 20 µm (x20) and 50 µm (x10) .

Appendix Figure S2

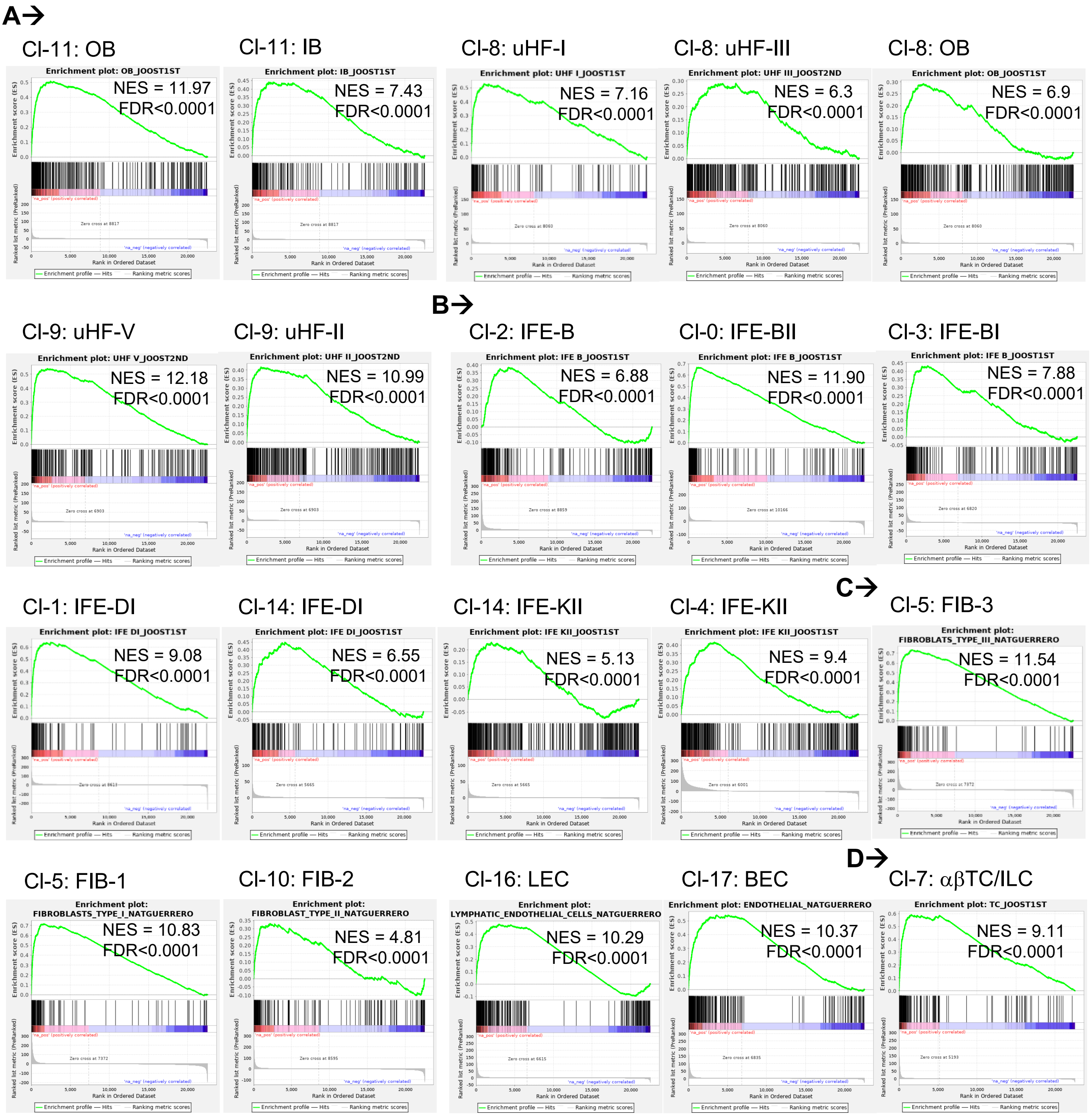

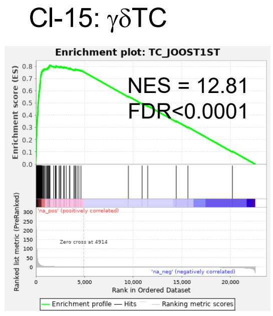

Appendix Figure S2 (Related to Figure 1). GSEA Identification of major naïve integrated cell clusters.

GSEA plots showing cluster cell type identification according to the ranked log2FC expression of all genes.

A. Hair follicle cells: outer/inner bulge (OB/IB), upper hair follicle (UHF) infundibular (INFU) cells.
B. Interfollicular Epidermis basal and differentiated cells (IFE-B, IFE-D, IFE-K).
C. Dermal stromal fibroblasts (FIB) and endothelial (LEC, BEC) cells.
D. Immune αβT and γδT cells.

Appendix Figure S3

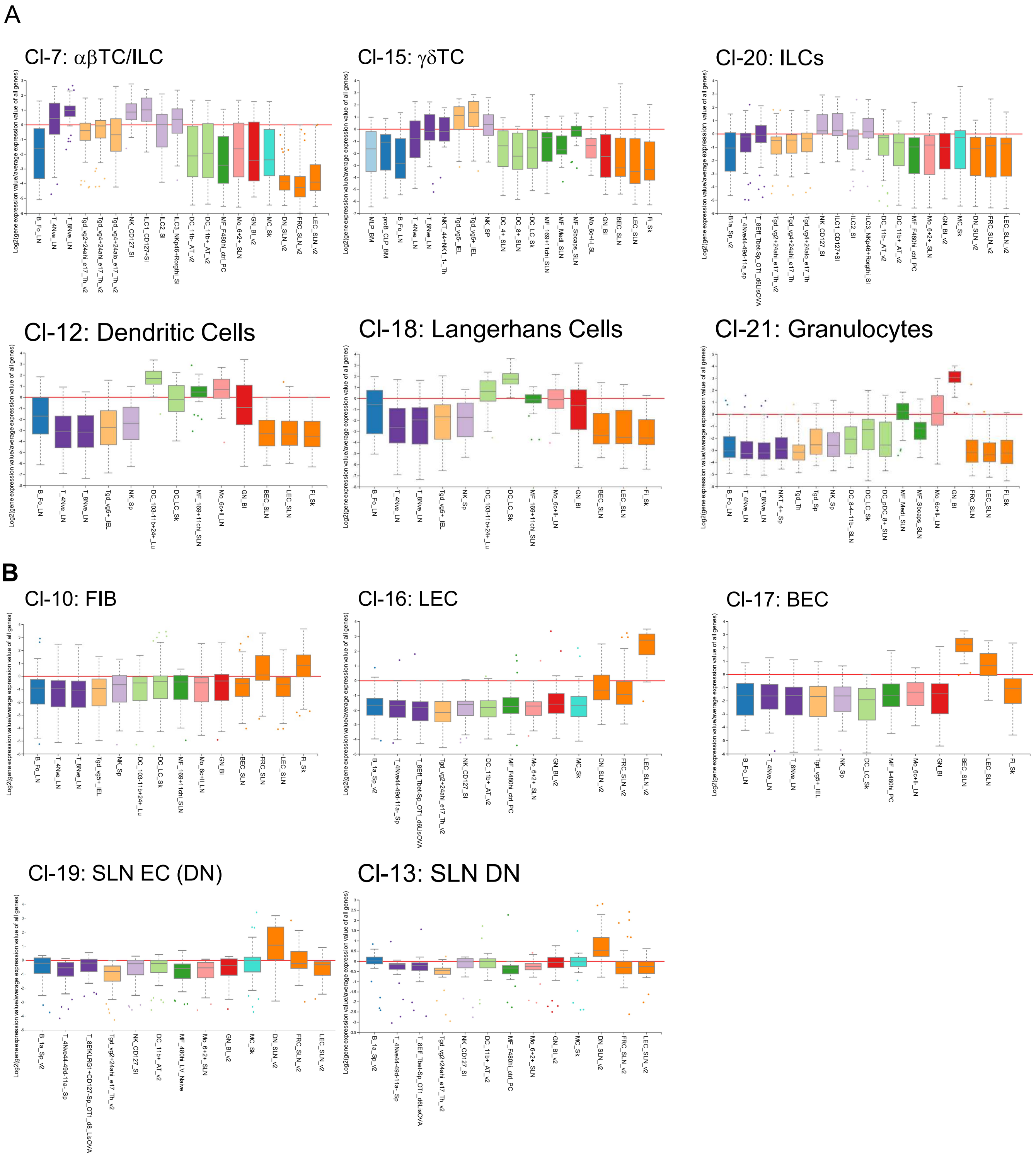

Appendix Figure S3 (Related to Figure 1). Cell cluster identity determined through ImmGen analysis.
W-plots generated by ImmGen analysis showing cluster identity for (A) Immune derived cell clusters and (B) stromal endothelial cell clusters. Analyses were performed by comparison of the top 50 most significant DE genes versus the ImmGen Microarray V1 dataset (Clusters 1,10,12,15,18) or the Microarray V2 dataset (clusters 7,13,16,17,19,20).

Appendix Figure S4

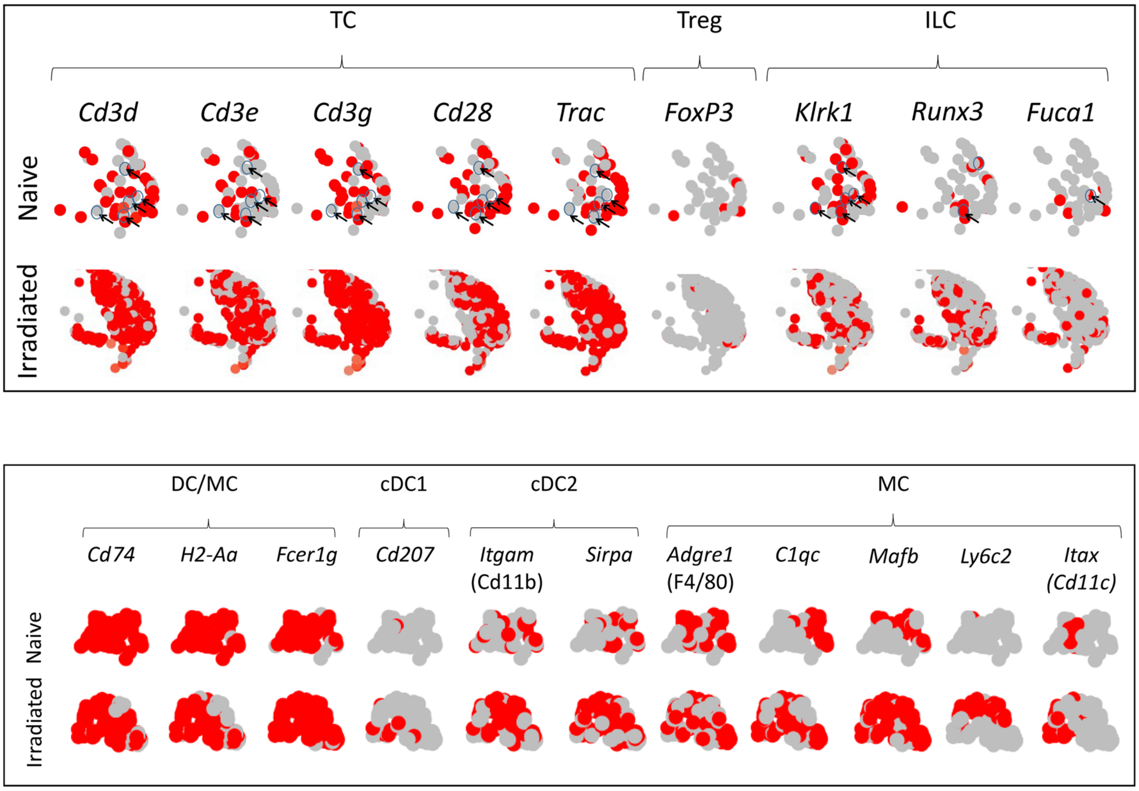

Appendix Figure S4 (Related to Figure 1). Marker genes expressed in αβT/ILC Cluster 7.
Top: UMAPs depicting relative expression (Red) of T cell-related and ILC-related markers genes (*Cd3, Cd28, Trac, Klrk1, Runx3, Fuca1, and FoxP3*) in Cluster 7 from naïve (top row) and irradiated (bottom row) mouse skin. Arrows with circles depict naive Klrk1^{+} Cd3^{-} cells.
Bottom: UMAPs depicting relative expression of markers related to dendritic cells (DC), conventional DC (cDC1 and cDC2), and monocyte-derived macrophages and dendritic cells (MC) *(Cd74, H2-Aa, Fcer1g, Cd207, Itgam, Sirpa, Adgre1, C1qc, Mafb, Ly6c2, and Itax*) in Cluster 12. Cells from naïve (top row) and irradiated (bottom row) mouse skin are shown independently.

Appendix Figure S5

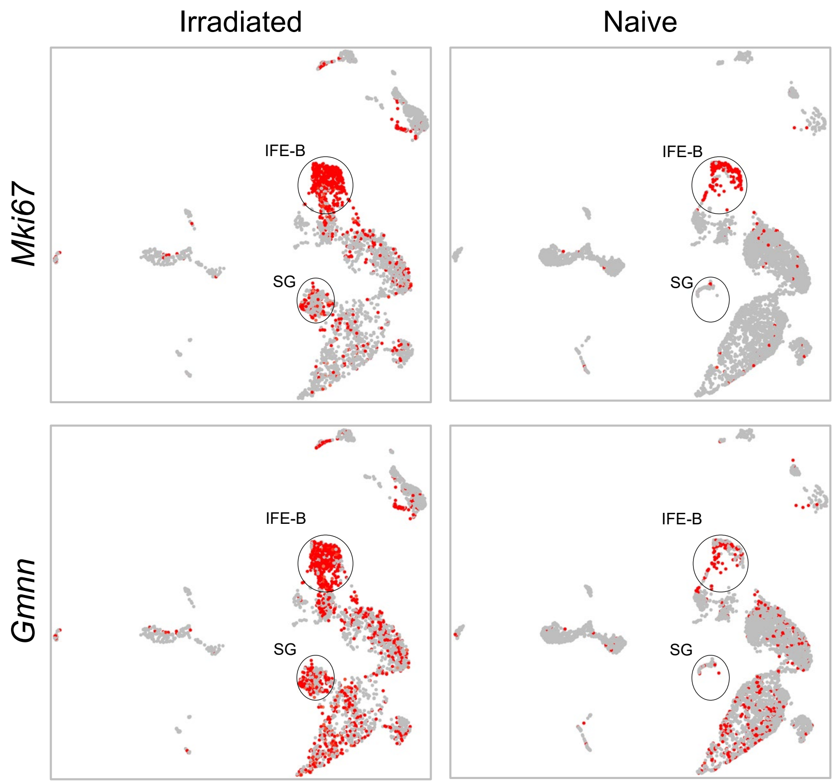

Appendix Figure S5 (Related to Figure 1). scRNA-seq identifies cycling cells in mouse skin. UMAPs depicting relative expression of cell cycle markers Ki-67 (*Mki67)* and Geminin (*Gmnn*) mRNAs in integrated skin clusters from naïve and irradiated mouse skin.

Appendix Figure S6

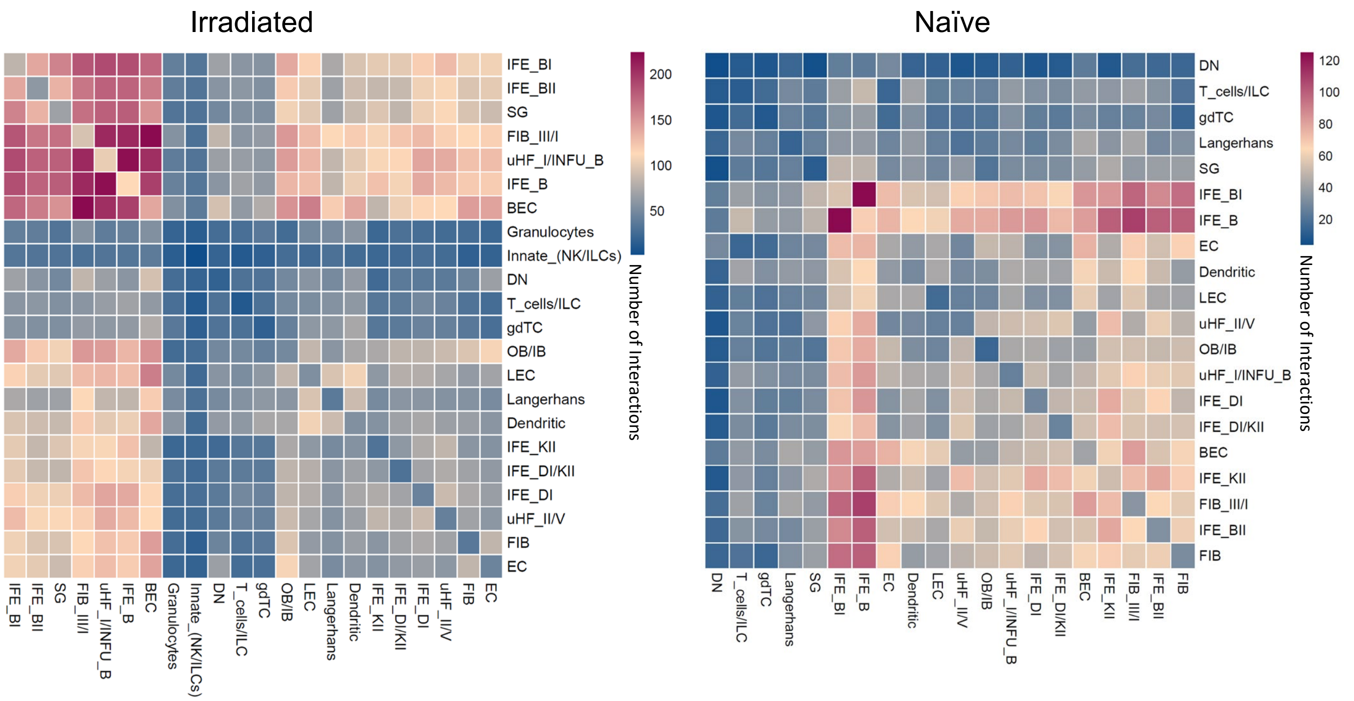

Appendix Figure S6 (Related to Figure 1). CellPhoneDB analysis shows the effect of irradiation on numbers of potential ligand-receptor interactions in mouse skin derived cell clusters.

Heatmaps generated by CellPhoneDB analysis depicting the total potential interaction number between scRNA-seq identified cell cluster types in irradiated (left) and naïve (right) skin datasets. The maps quantifies potential interactions based on gene expression levels but does not account for either boundaries of the cell types, the anatomical locations, or interactions involving agonist soluble ligand receptors.

Appendix Figure S7

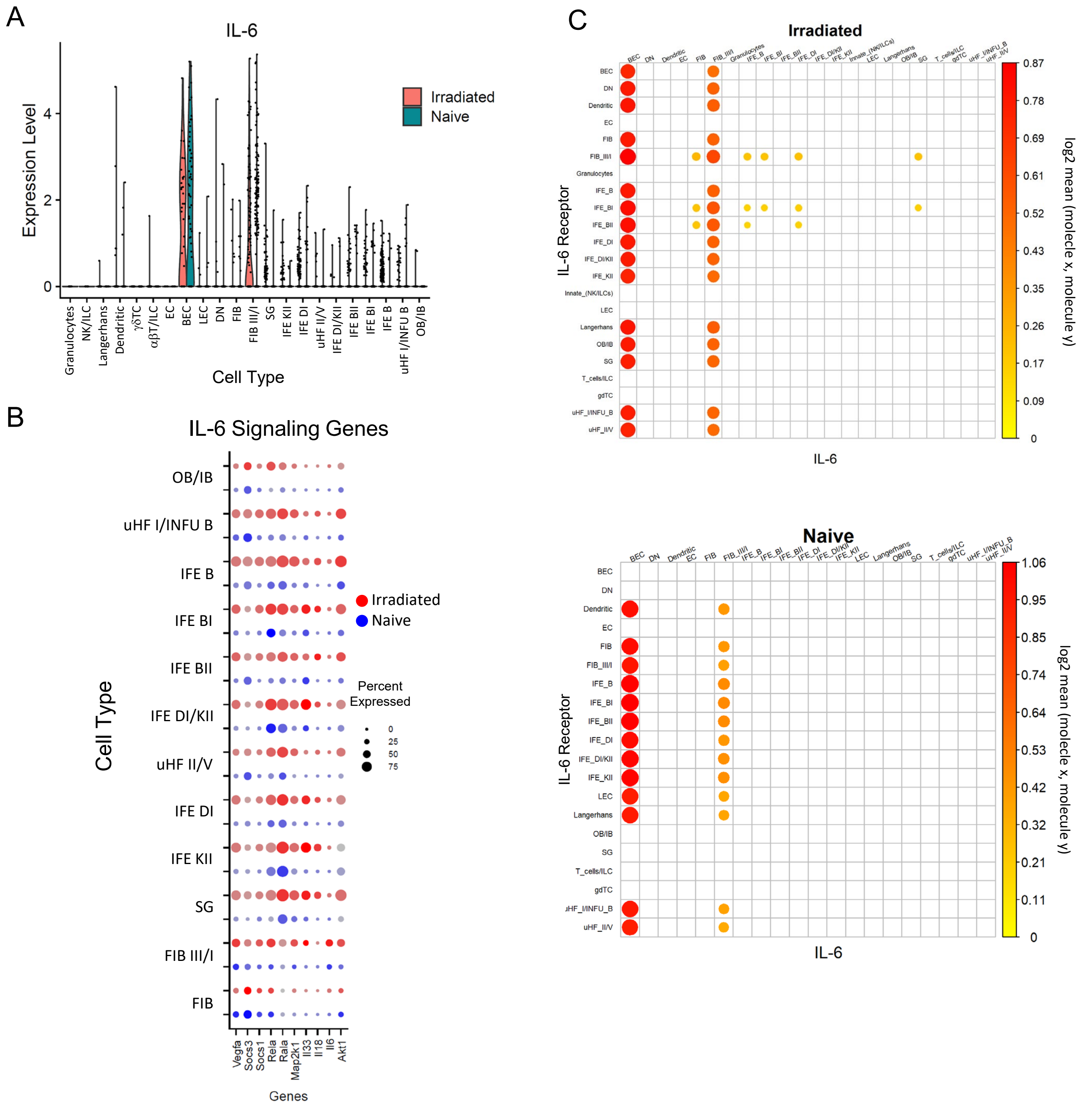

Appendix Figure S7 (Related to Figure 2). Irradiation increases potential IL-6 mediated ligand-receptor interactions between skin cell clusters.

A. Violin plot depicting IL-6 *(Il6)* mRNA expression in naïve and irradiated skin clusters.
B. Dot plot depicting the relative expression of selected IPA-defined downstream genes associated with IL-6 signaling in naïve (blue) and irradiated (red) scRNA-seq clusters.
C. Heatmaps depicting CellPhoneDB generated relative potential of IL-6 ligand-receptor interactions in naïve (bottom) and irradiated (top) skin cells according to cluster type. Color scale represents mean expression level of ligand and receptor (Mol1/2): log2 mean (molecule y, molecule x). $P < 0.05$ for all interactions shown.

Appendix Figure S8

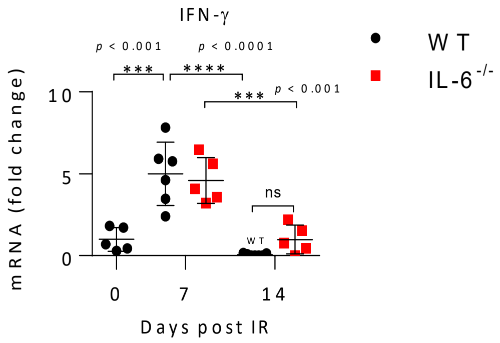

Appendix Figure S8 (Related to Figure 3). IR-induced IFN-γ mRNA upregulation in the skin is not affected by IL-6 deficiency.

Quantitative by real time qPCR analysis of IFN-γ mRNA in skin biopsies of naïve WT and irradiated (15 Gy) WT and IL-$6^{-/-}$ mice at indicated times post-IR, (n=5-7). Data are mean ± SD. ****P*<0.001, *****P*<0.0001 by one-way ANOVA with Tukey's multiple comparison test.

Appendix Figure S9

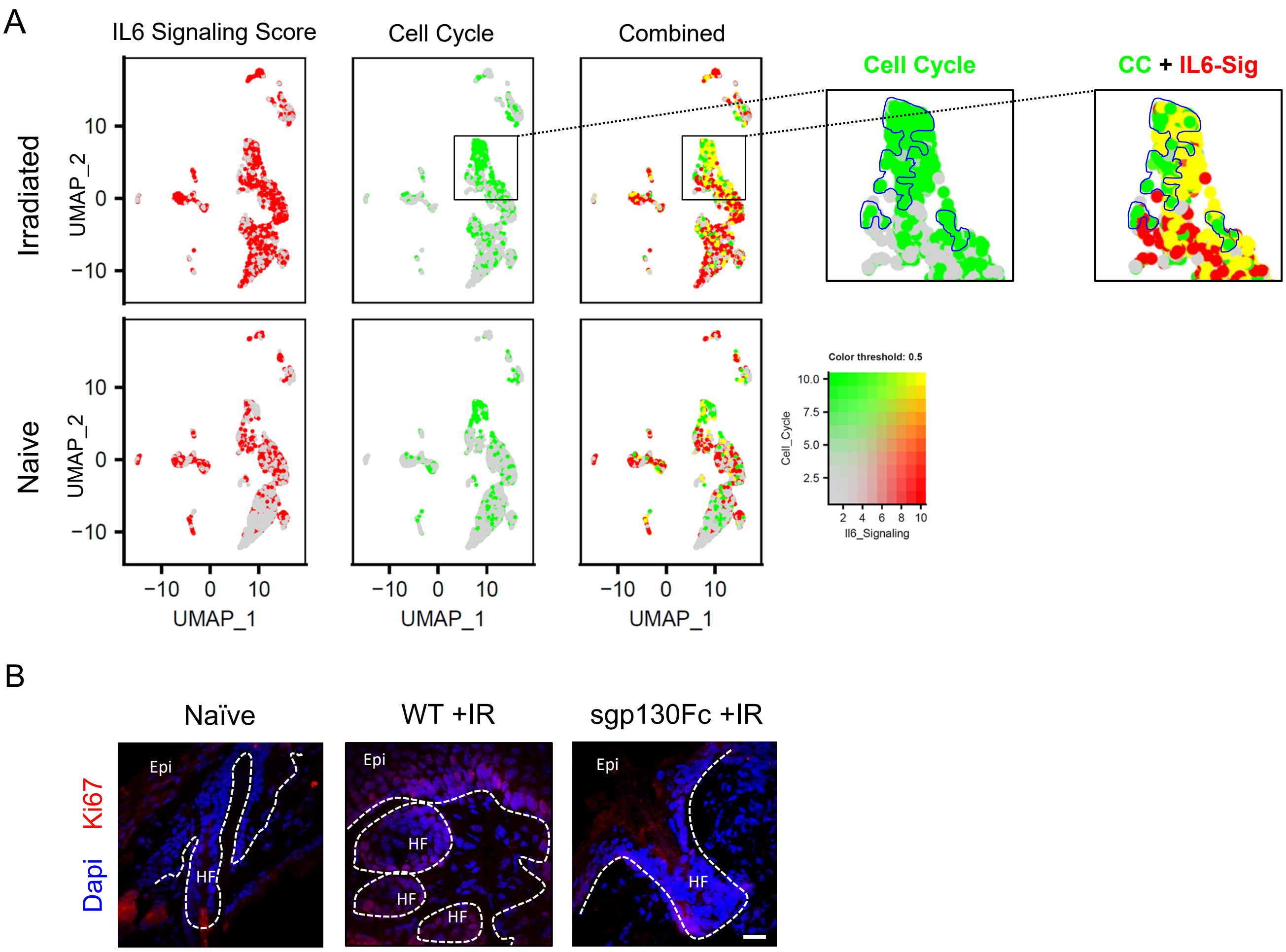

Appendix Figure S9 (Related to Figure 2). Partial overlap of IL-6 signaling with cell cycle scoring in irradiated epidermal cells.

A. Relative expression and co-localization of IL-6 Signaling (see Fig EV5) and cell cycle Scoring (*Mki67, Gmnn, Ccna1*) in integrated skin cells by blended UMAPs. Blowup: Regions of IEF-2 and uHF-I/INFU-B showing mitotic activity exclusive of IL-6 signaling (blue outline).

B. Photographic images showing nuclear IF staining for Ki67 (red) with Dapi (blue) counterstaining in skin biopsy thin sections from naïve and irradiated (14 Gy) wild type (WT) and sgp130Fc mice. White dashed lines demark epidermal (Epi) and hair follicle (HF) layers from dermal layers. Cells positively stained for Ki67 mainly appear in irradiated WT mice and largely within basal keratinocytes and hair follicles cells. Scale bar, 20µm.

Appendix Figure S10

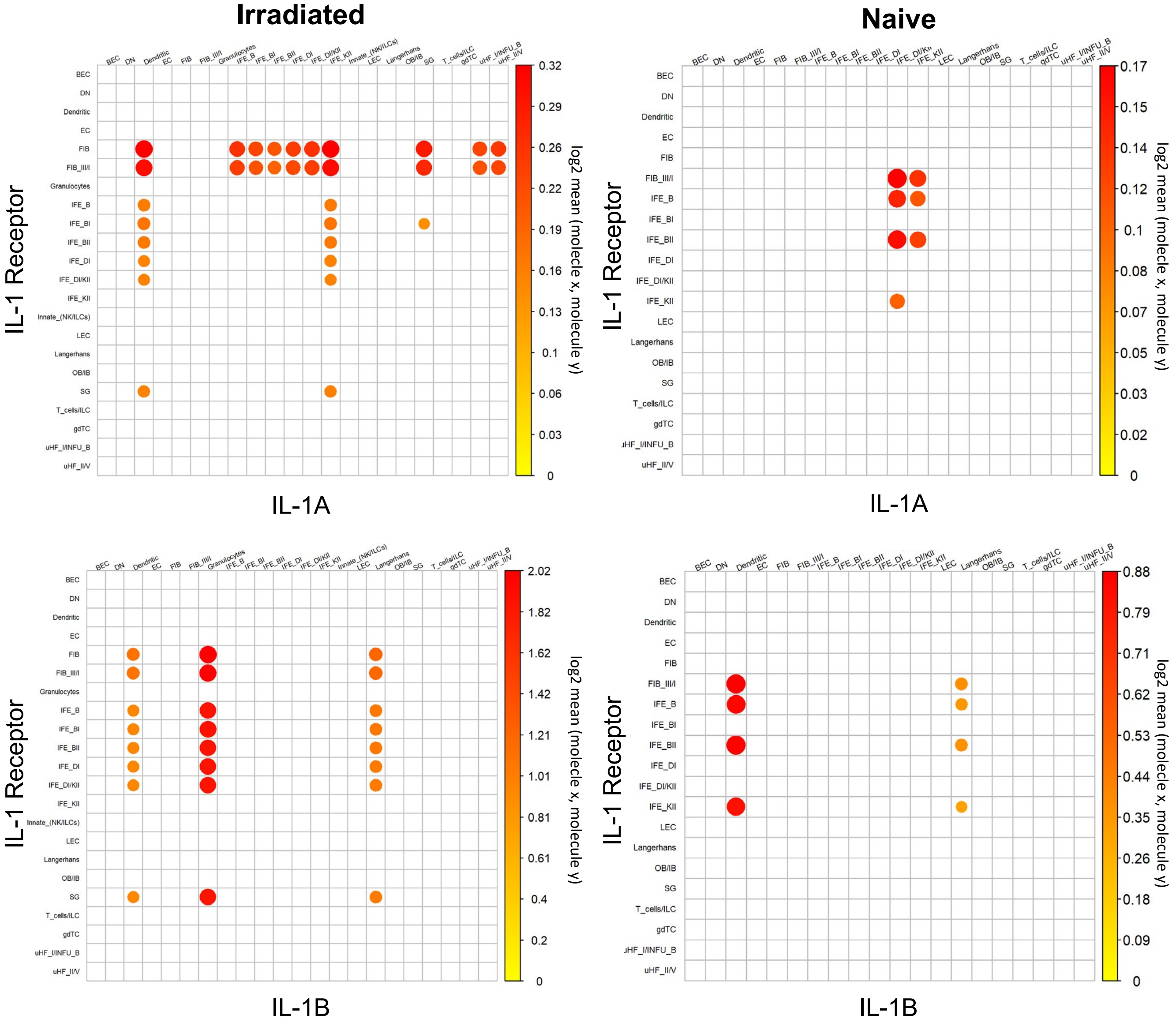

Appendix Figure S10 (Related to Fig 4). Irradiation increases IL-1 mediated ligand-receptor interaction potential between skin cell clusters.

Heatmaps depicting CellPhoneDB generated relative potential of IL-1A (Top) and IL-1B (Bottom) ligand-receptor interactions as indicated in naïve and irradiated (15Gy) skin cells according to cluster type. Color scale represents mean expression level of ligand and receptor (Mol1/2): log2 mean (molecule y, molecule x). *P* < 0.05.

Appendix Figure S11

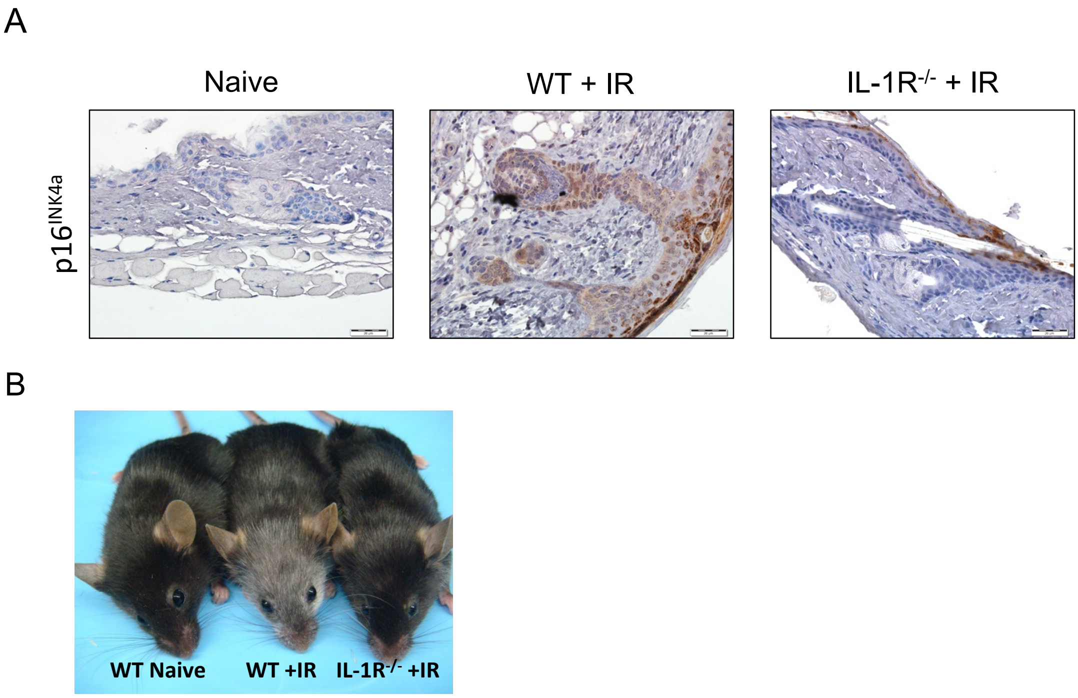

Appendix Figure S11 (Related to Fig 4). IL-1R deficiency reduces irradiation-induced p16^{INK4a} upregulation and hair depigmentation in mice.

A. Photographic image showing p16^{INK4a} immunostaining in skin paraffin embedded thin sections from WT naïve and irradiated (15 Gy) wild type (WT) and IL-6$^{-/-}$ mice 21 days post-IR. Scale bar, 20µm.

B. Photographic image showing hair depigmentation in representative naïve WT and irradiated (15Gy) WT and IL-1R$^{-/-}$ mice at 8 weeks post-IR. (Two independent experiments.)

Appendix Figure S12

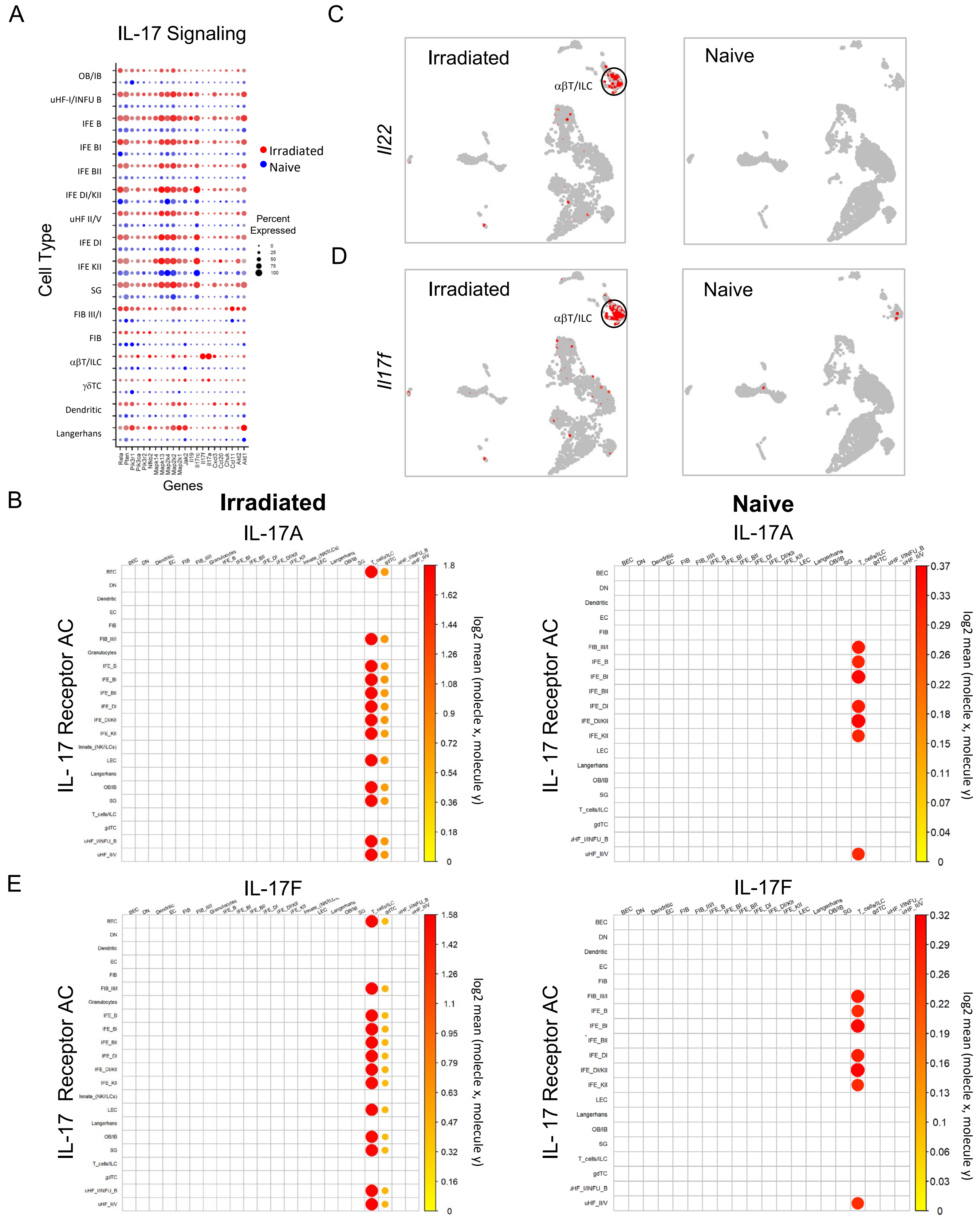

Appendix Figure S12 (Related to Fig EV4). Irradiation increases IL-22 (*Il22*) mRNA expression and IL-17 signaling potential between skin cell clusters.

A. Dot plot depicting expression of IPA-defined genes associated with IL-17 signaling in naïve (blue) and irradiated (red) scRNA-seq according to cell cluster.
B. Heatmaps depicting CellPhoneDB generated relative potential of IL-17A ligand-receptor interactions in irradiated and naïve skin cells according to cluster type. Color scale represents mean expression level of ligand and receptor (Mol1/2): log2 mean (molecule y, molecule x). $P < 0.05$.
C. UMAP plots depicting relative expression and localization of IL-22 (*Il22*) mRNA in irradiated and naïve skin derived $\alpha\beta$T cell clusters.
D. UMAP plots depicting relative expression and localization of IL-17F (*Il17f*) mRNA in irradiated and naïve skin T cell clusters.
E. Heatmaps depicting CellPhoneDB generated relative potential of IL-17F ligand-receptor interactions, as in (B).

Appendix Figure S13

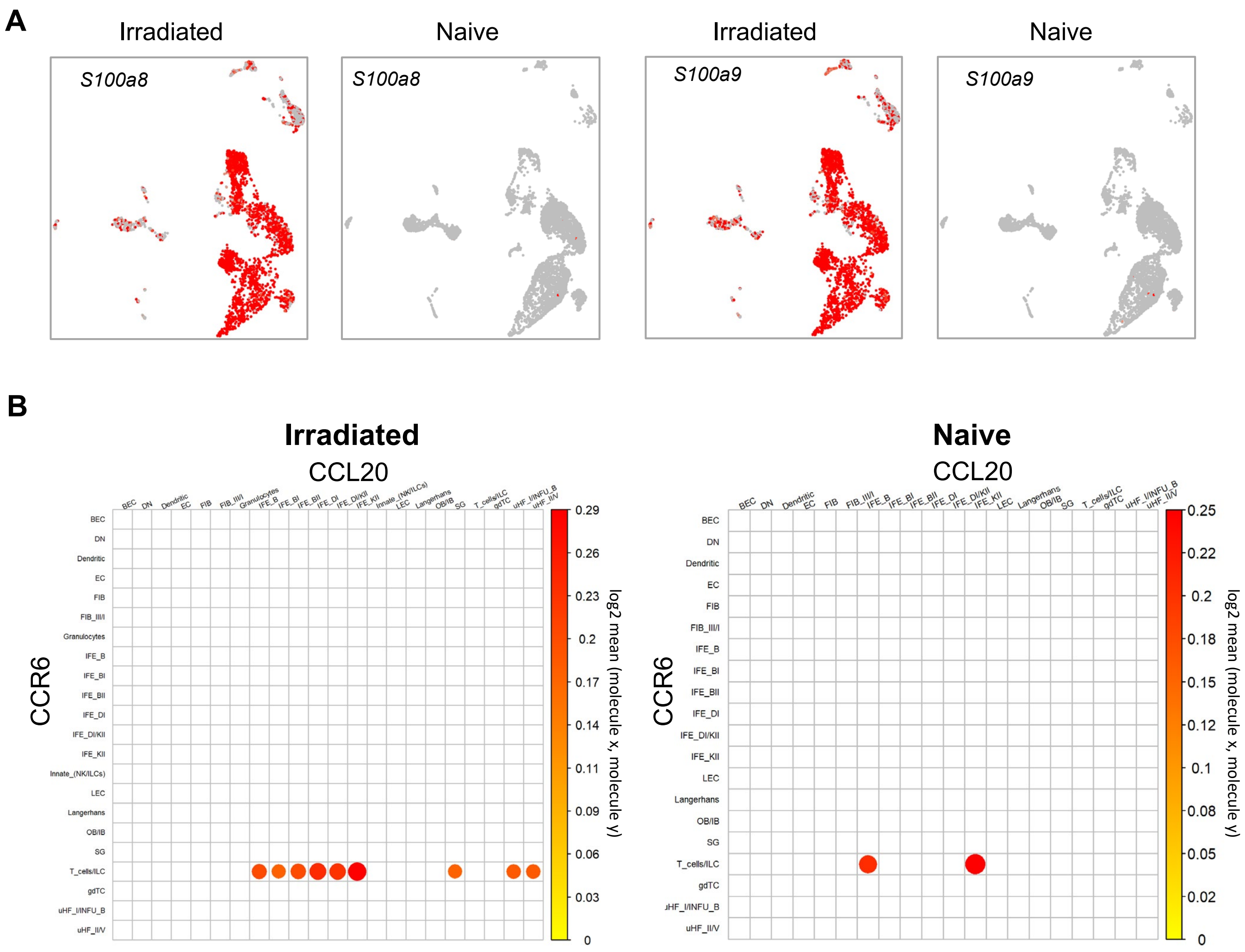

Appendix Figure S13 (Related to Fig 5). Irradiation-induced alarmin expression and CCL20-CCR6 interaction potential concentrate within epidermal skin cell clusters.

A. UMAP plots depict expression and localization of alarmins, *S100a8* and *S100a9,* mRNAs in irradiated (15Gy) versus naïve skin derived cell clusters.

B. Heatmaps depicting relative potential CCL20 and CCR6 interactions in naïve and irradiated (15Gy) skin cells generated by CellPhoneDB analysis. Color scale represents mean expression level of ligand and receptor (Mol1/2): log2 mean (molecule y, molecule x). $P < 0.05$.

Appendix Figure S14

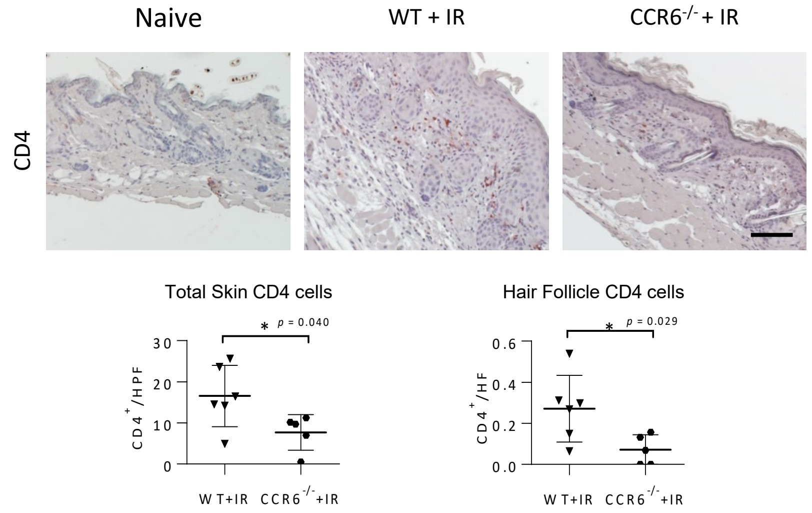

Appendix Figure S14 (Related to Figure 5). Ccr6 deficiency reduces irradiation-induced CD4+ T cell infiltration to the dermis and hair follicles in mice.

Infiltration of CD4+ T helper cells is shown by immunostaining (red) of skin thin sections from naïve WT mice and irradiated (15 Gy) WT and CCR6-/- mice 14 days post-IR. Scale bar, 50 µm. Quantification (below) of infiltrating CD4+ cells in the dermis and hair follicles (HF) in irradiated WT and CCR6-/- mice 14 days post-IR, (n = 5-6). Data are means ± SD. *P < 0.05 by two-tailed Student's *t* test.

Appendix Figure S15

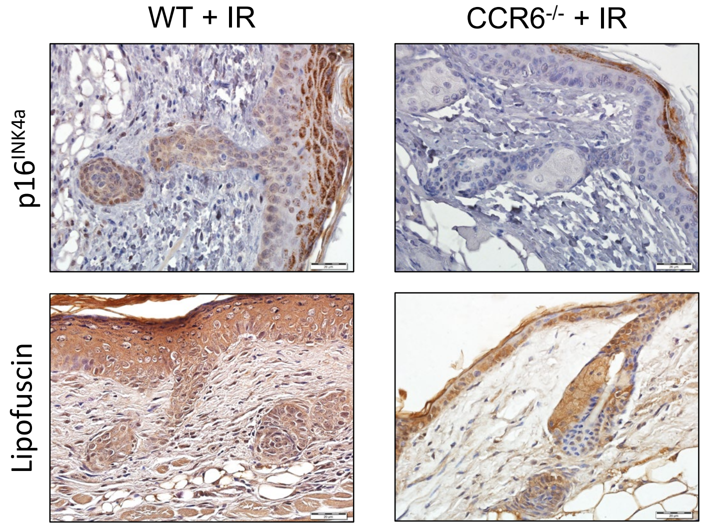

Appendix Figure S15 (Related to Figure 5). Ccr6 deficiency reduces irradiated-induced cellular senescence-associated markers in mouse skin.

Photographic images of skin thin sections showing immunostaining (brown stain) for p16INK4a and lipofuscin (SenTraGor) in irradiated (15 Gy) wild type (WT) and Ccr6-/- mice, 21 days post-IR . Scale bar, 20µm.

Appendix Figure S16

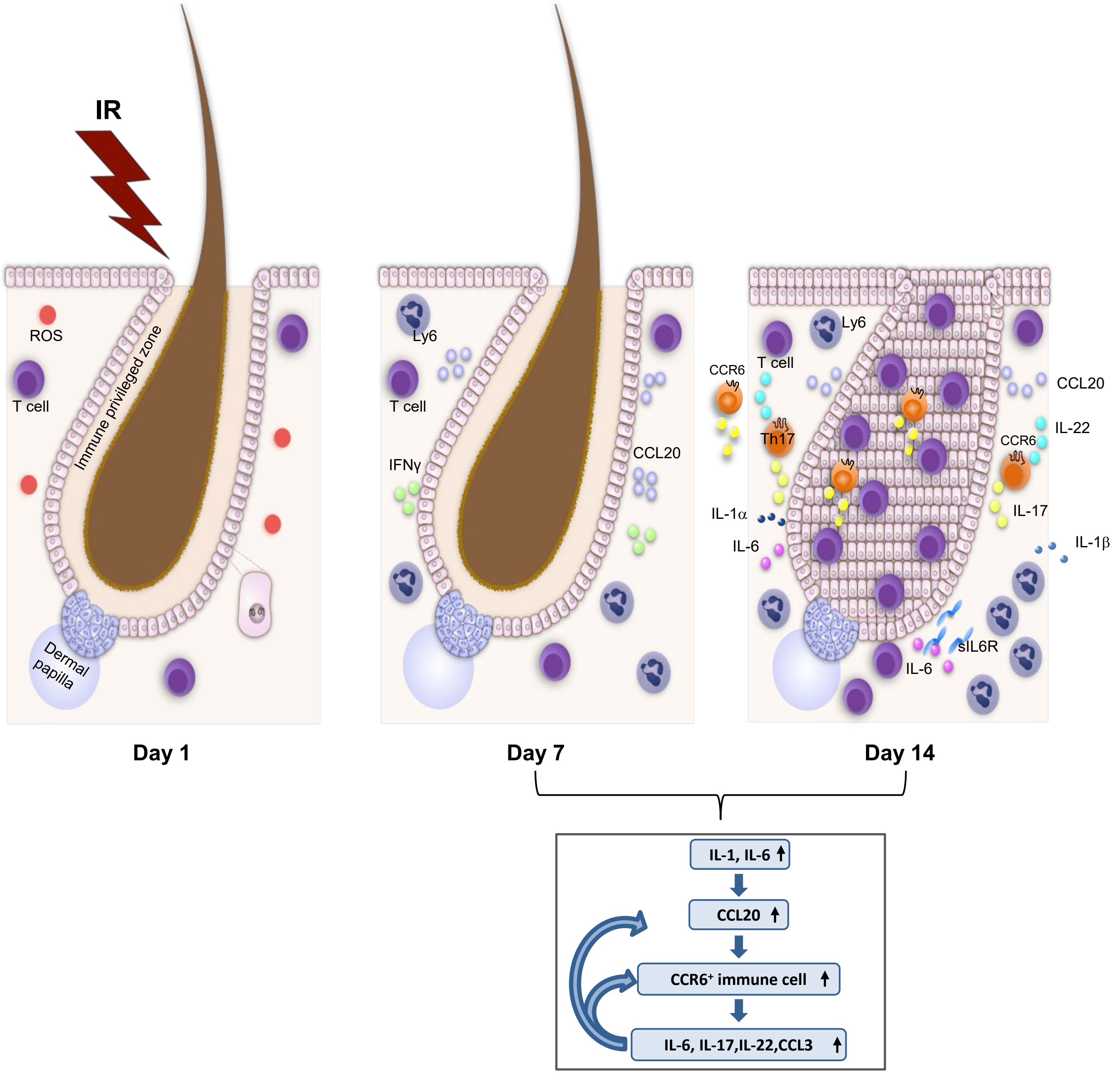

Appendix Figure S16. A schematic model of senescence-associated IL-6/IL-1/CCR6 axis in irradiation-induced dermatitis and hair loss.

IL-1, IL-6 and CCL20 cooperate to induce infiltration, differentiation and activation of CCR6^{+} effector cells, such as Th17 cells, to irradiated skin and hair follicles. Progressive IL-6/Stat3-dependent cytokine and chemokine signaling, also involving IL-17 and IL-22, and CCL3 upregulation, generates an IL-6/IL-1/CCR6 feedback loop. This in turn promotes maintenance of senescence, keratinocyte hyperplasia, and further immune cell recruitment that drives the collapse of the hair follicle immune privilege status, leading to hair follicle degeneration.
