# Supplementary material for: Single‐cell transcriptomics reveals a senescence‐associated IL‐6/CCR6 axis driving radiodermatitis
Source: EMBO Mol Med. 2022 Jul 4;14(8):e15653. doi: 10.15252/emmm.202115653 (PMC9358397; doi:10.15252/emmm.202115653)
Supplement: Supplementary file 2 — Expanded View Figures PDF [file EMMM-14-e15653-s001.pdf]

Expanded View Figures

Figure EV1. (Related to Fig 1). Relative mutually exclusive senescence and cell cycle scoring in naïve and irradiated epidermal cells.

A Relative expression and co-localization of Senescence Score (red) and Cell Cycle Score (*Mki67*, *Gmnn*, *Ccna1*; green) in integrated skin cells by blended UMAPs. Blowup: Regions of IEF-2 and uHF-I/INFU-B showing exclusive mitotic activity (outlined in blue).

B Dot plot depicting the relative expression of selected IPA-defined senescence-related markers and cell cycle markers (framed in red) (*x*-axis) in naïve (blue dots) and irradiated (red dots) scRNA-seq skin cell clusters.

C UMAP plots depicting relative expression and localization of urokinase-type plasminogen activator receptor (*Plaur*) mRNA in irradiated and naïve skin-derived clusters.

D Immunostaining and histochemical staining (brown) of urokinase-type plasminogen activator receptor (uPAR), $p16^{INK4a}$, and lipofuscin (SenTraGor®) in paraffin-embedded skin thin sections from ventral neck and upper chest region of naïve and irradiated (15 Gy) wild type mice taken at 21 days post-IR. $p16^{INK4a}$ immunostaining appears as nuclear and/or cytoplasmic staining (Arrows) in IFE keratinocytes, in cells comprising discrete hair follicle layers, and in distinct cells in the dermis. Scale bars, 20 μm.

© 2022 The Authors

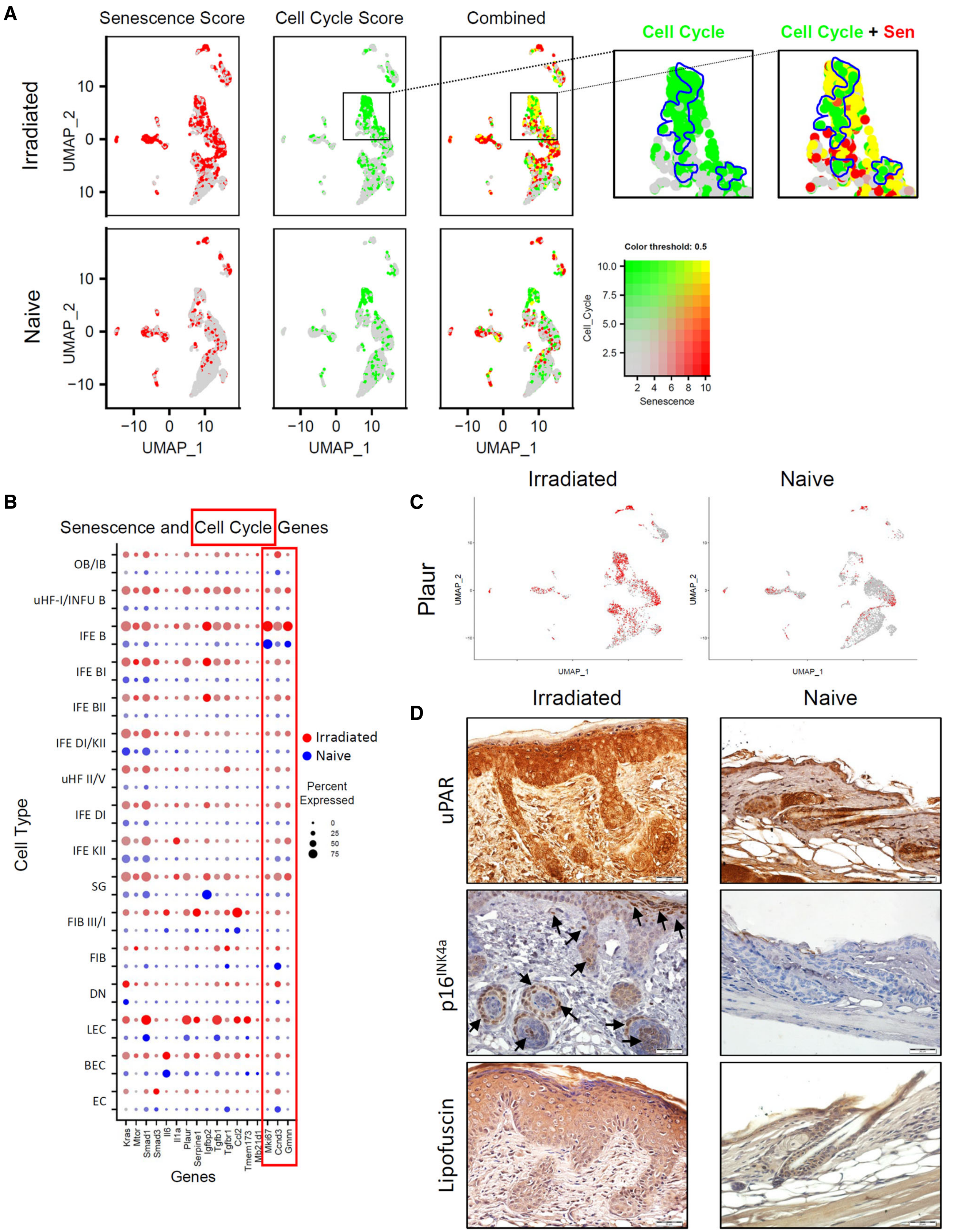

Figure EV1.

 © 2022 The Authors

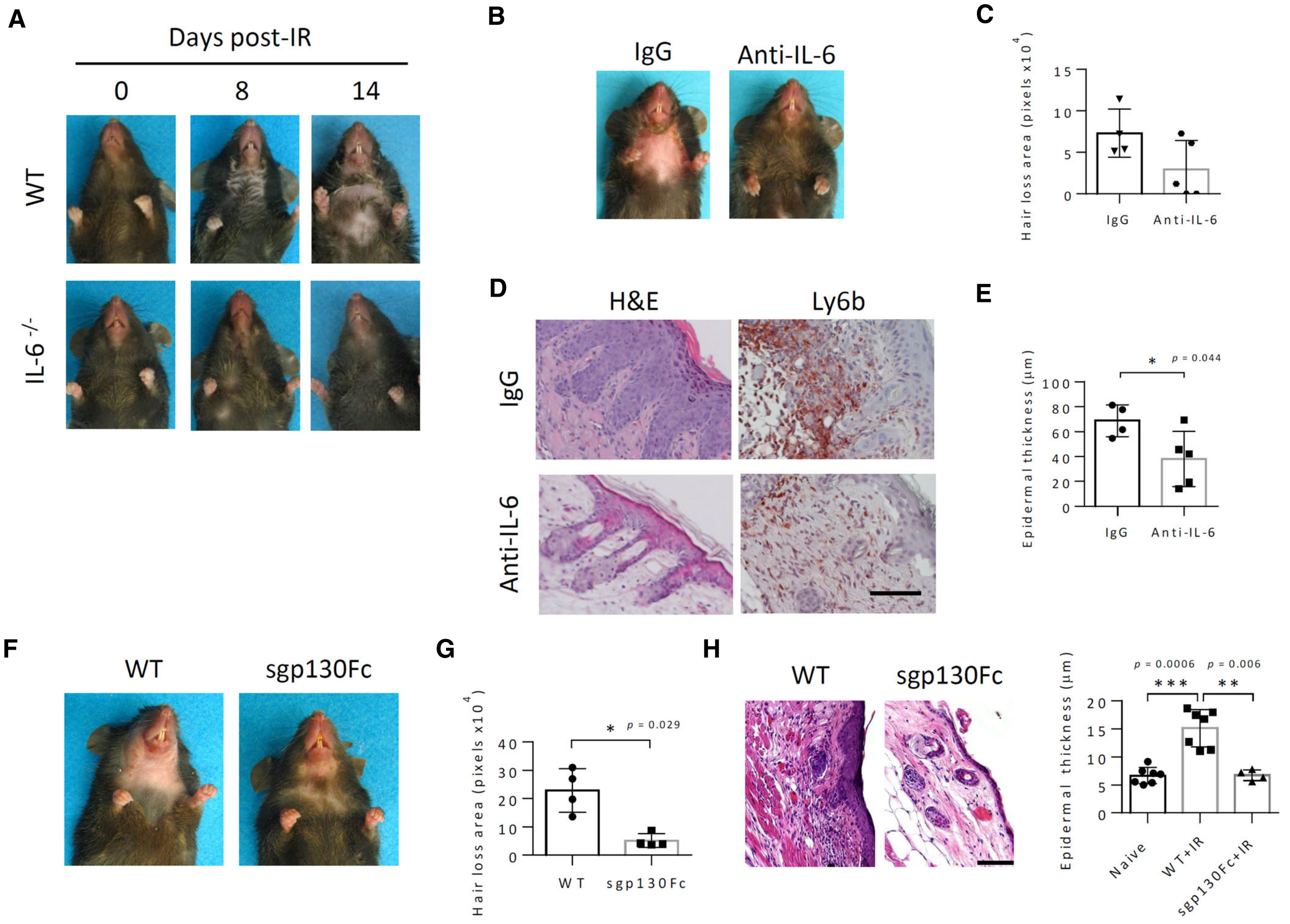

Figure EV2. (Related to Fig 2). IL-6 signaling blockade reduces hair loss and dermatitis in irradiated mice.

- A Photographic images depicting the kinetics of radiodermatitis and hair loss in ventral views of representative wild-type mice irradiated (15 Gy) to the head and neck. Times (in days) post-IR are indicated. Serous exudates appear under the chin at 8 days post-IR with desquamation and alopecia beginning at about 14 days post-IR.
- B Photographic images showing ventral alopecia in wild-type mice treated with neutralizing IL-6 mAb (Anti-IL-6) or control mAb (IgG; 200 µg, i.p) prior to and 8 days following irradiation (14 Gy) to the head and neck. Two independent experiments.
- C Quantification of the area of hair loss of mice in (B) at 14 days post-IR, (n = 4–5). Data are individual mice. Two independent experiments.
- D Histochemical (H&E) and immunostaining of skin thin sections from mice in (B) showing morphology and neutrophil (Ly6b) infiltration (red staining). Scale bar, 50 µm.
- E Quantification of epidermal thickness in specimens from (D), (n = 4–5).
- F Photographic images showing ventral alopecia in wild-type mice (WT) and sgp130Fc transgenic mice irradiated (14 Gy) to the head and neck 21 days post-IR. Five independent experiments.
- G Quantification of the area of hair loss of mice in (F), (n = 4).
- H Histochemical (H&E) staining of skin thin sections from mice in (F) and quantification of the epidermal thickness (right), (n = 4–7). Scale bar, 100 µm.

Data information: Data are mean ± SD. *$P < 0.05$, **$P < 0.01$, ***$P < 0.001$ by two-tailed Student's t-test (E), or Mann–Whitney test (G, H).
Source data are available online for this figure.

Figure EV3. (Related to Fig 2). Substantial overlap of IL-6 signaling and cellular senescence in irradiated mouse skin.

- A Relative expression and co-localization of IL-6 Signaling (Red; see also Appendix Fig S7B) and Senescence Pathway (Green; see also Fig EV1B) scoring in integrated skin cells by blended UMAPs.
- B Photographic images showing immunostaining of p16^{INK4a} (arrows) and lipofuscin (brown stain) in irradiated (15 Gy) IL-6$^{-/-}$ mice skin thin sections compared to irradiated wild type (WT) mice 21 days post-IR WT. Scale bar, 20 µm.
- C Photographic images showing hair depigmentation in representative naïve WT and irradiated (15 Gy) WT and IL-6$^{-/-}$ mice at 8 weeks post-IR. (Four independent experiments.)

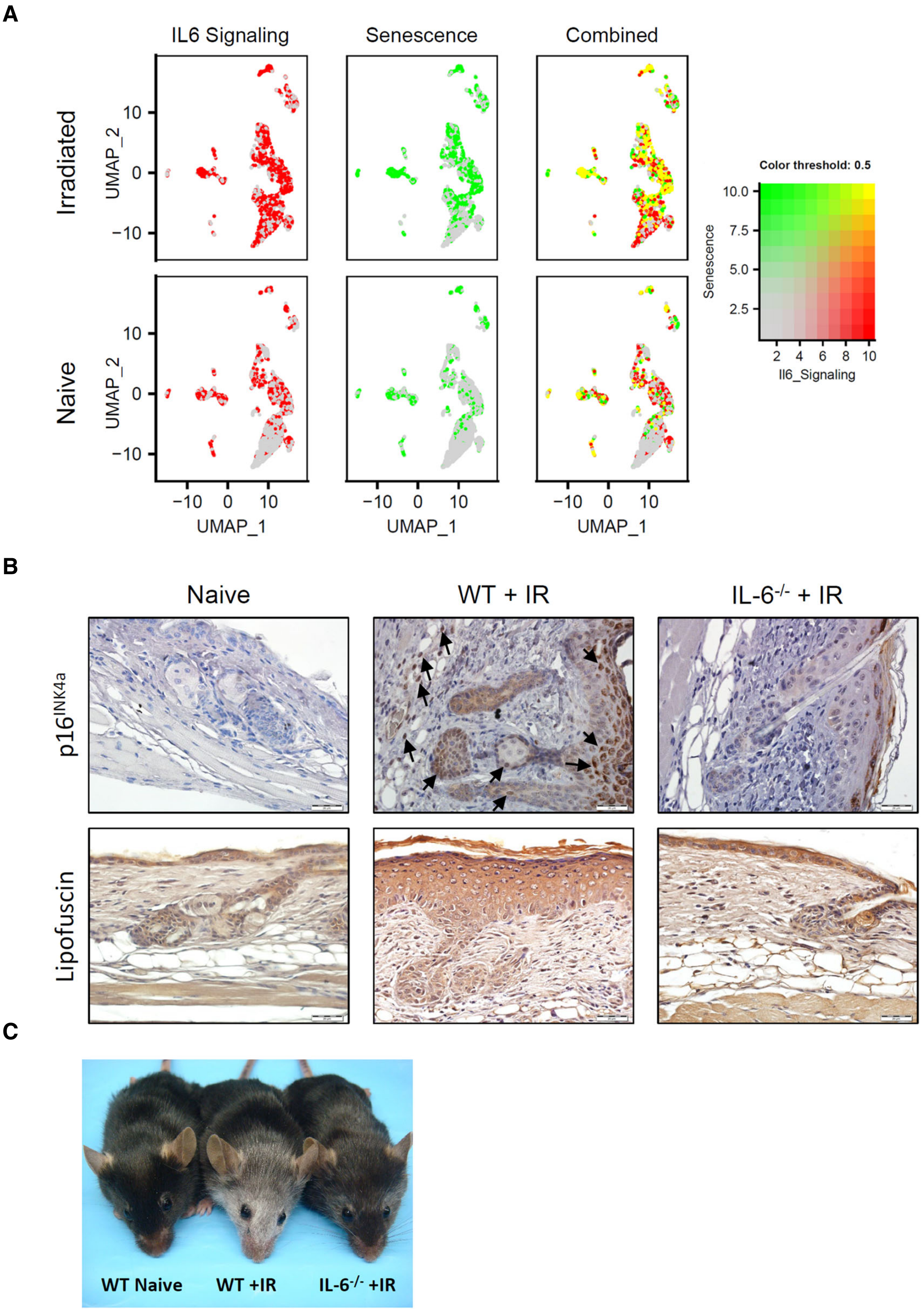

Figure EV3.

© 2022 The Authors

Figure EV4. IL-17A deficiency moderately alleviates IRIAD in mice.

A Relative expression and co-localization of *Il17a* mRNA and IL-17 Signaling in integrated skin cells by blended UMAPs.
B Quantification by real-time qPCR analysis of *Il17a* and *Il22* mRNAs in whole skin of naïve and irradiated (15 Gy) WT and IL-$6^{-/-}$ mice at indicated times post-IR, (n = 4–7).
C Photographic images showing hair loss and radiodermatitis at 14 days post-IR (15 Gy) in representative WT and IL-$17^{-/-}$ mice and quantification (below) of the area of ventral hair loss, (n = 6–8).
D Histochemical staining (H&E), immunostaining (red), and quantification (right) of acanthosis and infiltration of neutrophils (Ly6b^{+}) and T cell (CD3^{+}; red arrows) to the skin and to the hair follicle (HF; dashed lines) in irradiated WT and mice in (C), (n = 6–7). Scale bars, 50 μm (black) and 25 μm (red).
E Quantification of normal and degenerate hair follicles shown in H&E sections (left) and of T cell (CD3) infiltration into the hair follicle (HF; right) shown in (D), (n = 6–7).
F Quantification by real-time qPCR analysis of *Il6* and *Ccl3* mRNAs in the skin of WT and IL-$6^{-/-}$ mice before (naïve) and 14 days post-IR (15 Gy, n = 4–7).

Data information: Data represent mean ± SD. *$P < 0.05$, **$P < 0.01$ by two-tailed Mann–Whitney test; two independent experiments (See also Appendix Fig S12).

© 2022 The Authors

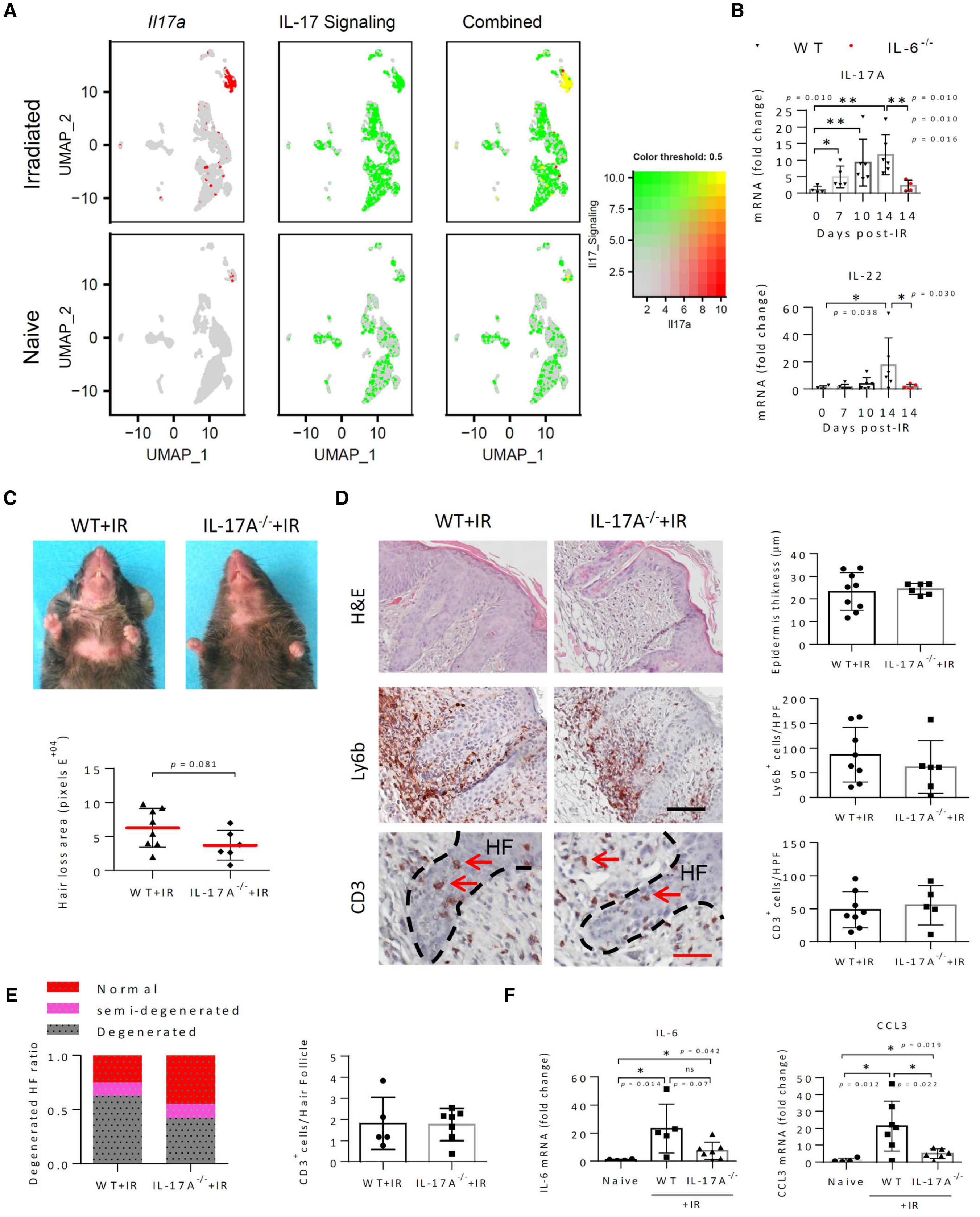

Figure EV4.

 © 2022 The Authors
