# Supplementary material for: Single‐cell transcriptomics reveals a senescence‐associated IL‐6/CCR6 axis driving radiodermatitis
Source: EMBO Mol Med. 2022 Jul 4;14(8):e15653. doi: 10.15252/emmm.202115653 (PMC9358397; doi:10.15252/emmm.202115653)
Supplement: Supplementary file 10 [file EMMM-14-e15653-s003.pdf]

EMBO Molecular Medicine

Single-cell transcriptomics reveals a senescence-associated IL-6/CCR6 axis driving radiodermatitis

Mor Paldor, Orr Levkovitch-Siany, Dana Eidelshtein, Claes Enk, Revital Adar, Yitzhak Marmary, Sharona Elgavish, Yuval Nevo, Hadar Benyamini, Inbar Plaschkes, Shiri Klein, Alexander Maly, Stefan Rose-John, Amnon Peled, Eithan Galun, and Jonathan Axelrod
DOI: 10.15252/emmm.202115653

Corresponding author(s): Jonathan Axelrod (axelrod@hadassah.org.il) , Eithan Galun (EithanG@hadassah.org.il)

Review Timeline:

Submission Date:	29th Dec 21
Editorial Decision:	28th Jan 22
Revision Received:	9th May 22
Editorial Decision:	24th May 22
Revision Received:	31st May 22
Accepted:	1st Jun 22

Editor: Lise Roth

Transaction Report:

(Note: With the exception of the correction of typographical or spelling errors that could be a source of ambiguity, letters and reports are not edited. Depending on transfer agreements, referee reports obtained elsewhere may or may not be included in this compilation. Referee reports are anonymous unless the Referee chooses to sign their reports.)

28th Jan 2022

Dear Dr. Axelrod,

Thank you for the submission of your manuscript to EMBO Molecular Medicine. We have now received feedback from the three reviewers who agreed to evaluate your manuscript. As you will see from the reports below, the referees acknowledge the interest of the study and are overall supporting publication of your work pending appropriate revisions.

Addressing the reviewers' concerns in full will be necessary for further considering the manuscript in our journal, and acceptance of the manuscript will entail a second round of review. EMBO Molecular Medicine encourages a single round of revision only and therefore, acceptance or rejection of the manuscript will depend on the completeness of your responses included in the next, final version of the manuscript. For this reason, and to save you from any frustrations in the end, I would strongly advise against returning an incomplete revision.

Revised manuscripts should be submitted within three months of a request for revision; they will otherwise be treated as new submissions, except under exceptional circumstances in which a short extension is obtained from the editor.

When submitting your revised manuscript, please carefully review the instructions that follow below. We perform an initial quality control of all revised manuscripts before re-review; failure to include requested items will delay the evaluation of your revision.

We require:

1) A .docx formatted version of the manuscript text (including legends for main figures, EV figures and tables). Please make sure that the changes are highlighted to be clearly visible.

2) Individual production quality figure files as .eps, .tif, .jpg (one file per figure). For guidance, download the 'Figure Guide PDF' (https://www.embopress.org/page/journal/17574684/authorguide#figureformat).

3) A .docx formatted letter INCLUDING the reviewers' reports and your detailed point-by-point responses to their comments. As part of the EMBO Press transparent editorial process, the point-by-point response is part of the Review Process File (RPF), which will be published alongside your paper.

4) A complete author checklist, which you can download from our author guidelines (https://www.embopress.org/page/journal/17574684/authorguide#submissionofrevisions). Please insert information in the checklist that is also reflected in the manuscript. The completed author checklist will also be part of the RPF.

5) It is mandatory to include a 'Data Availability' section after the Materials and Methods. Before submitting your revision, primary datasets produced in this study need to be deposited in an appropriate public database, and the accession numbers and database listed under 'Data Availability'. Please remember to provide a reviewer password if the datasets are not yet public (see https://www.embopress.org/page/journal/17574684/authorguide#dataavailability).
In case you have no data that requires deposition in a public database, please state so in this section. Note that the Data Availability Section is restricted to new primary data that are part of this study.

6) For data quantification: please specify the name of the statistical test used to generate error bars and P values, the number (n) of independent experiments (specify technical or biological replicates) underlying each data point and the test used to calculate p-values in each figure legend. The figure legends should contain a basic description of n, P and the test applied. Graphs must include a description of the bars and the error bars (s.d., s.e.m.). Exact p values should be provided.

7) We would also encourage you to include the source data for figure panels that show essential data. Numerical data should be provided as individual .xls or .csv files (including a tab describing the data). For blots or microscopy, uncropped images should be submitted (using a zip archive if multiple images need to be supplied for one panel).

8) Our journal encourages inclusion of *data citations in the reference list* to directly cite datasets that were re-used and obtained from public databases. Data citations in the article text are distinct from normal bibliographical citations and should directly link to the database records from which the data can be accessed. In the main text, data citations are formatted as follows: "Data ref: Smith et al, 2001" or "Data ref: NCBI Sequence Read Archive PRJNA342805, 2017". In the Reference list, data citations must be labeled with "[DATASET]". A data reference must provide the database name, accession number/identifiers and a resolvable link to the landing page from which the data can be accessed at the end of the reference. .

9) We replaced Supplementary Information with Expanded View (EV) Figures and Tables that are collapsible/expandable online. A maximum of 5 EV Figures can be typeset. EV Figures should be cited as 'Figure EV1, Figure EV2" etc... in the text and their respective legends should be included in the main text after the legends of regular figures.
- For the figures that you do NOT wish to display as Expanded View figures, they should be bundled together with their legends in a single PDF file called *Appendix*, which should start with a short Table of Content. Appendix figures should be referred to in the main text as: "Appendix Figure S1, Appendix Figure S2" etc.
- Additional Tables/Datasets should be labeled and referred to as Table EV1, Dataset EV1, etc. Legends have to be provided in a separate tab in case of .xls files. Alternatively, the legend can be supplied as a separate text file (README) and zipped together with the Table/Dataset file.

10) Conflict of interest: We updated our journal's competing interests policy in January 2022 and request authors to consider both actual and perceived competing interests. Please review the policy https://www.embopress.org/competing-interests and update your competing interests if necessary.

11) Every published paper now includes a 'Synopsis' to further enhance discoverability. Synopses are displayed on the journal webpage and are freely accessible to all readers. They include a short stand first (maximum of 300 characters, including space) as well as 2-5 one-sentences bullet points that summarizes the paper. Please write the bullet points to summarize the key NEW findings. They should be designed to be complementary to the abstract - i.e. not repeat the same text. We encourage inclusion of key acronyms and quantitative information (maximum of 30 words / bullet point). Please use the passive voice. Please attach these in a separate file or send them by email, we will incorporate them accordingly.
Please also suggest a striking image or visual abstract to illustrate your article as a PNG file 550 px wide x 300-600 px high.

12) As part of the EMBO Publications transparent editorial process initiative (see our Editorial at http://embomolmed.embopress.org/content/2/9/329), EMBO Molecular Medicine will publish online a Review Process File (RPF) to accompany accepted manuscripts.
In the event of acceptance, this file will be published in conjunction with your paper and will include the anonymous referee reports, your point-by-point response and all pertinent correspondence relating to the manuscript. Let us know whether you agree with the publication of the RPF and as here, if you want to remove or not any figures from it prior to publication.
Please note that the Authors checklist will be published at the end of the RPF.

EMBO Molecular Medicine has a "scooping protection" policy, whereby similar findings that are published by others during review or revision are not a criterion for rejection. Should you decide to submit a revised version, I do ask that you get in touch after three months if you have not completed it, to update us on the status.

I look forward to receiving your revised manuscript.

With kind regards,

Lise Roth

Lise Roth, PhD
Editor
EMBO Molecular Medicine

***** Reviewer's comments *****

Referee #1 (Comments on Novelty/Model System for Author):

Timely study involving state-of-art methodology. Appropriate choice of in vivo model in light of difficulties associated with carrying out similar studies in human. Data are clearly presented and discussed.

Referee #1 (Remarks for Author):

Although common, the mechanisms underlying irradiation-induced skin damage during cancer treatment are not known in detail. Hopefully, thanks to novel irradiation methods (proton beam therapy), these severe complications may become a thing of the past. Nevertheless, the present study examined the effect of high-dose irradiation on head and neck skin in mice by a combination of scRNAseq analyses and histology and the use of gene-deficient mice. The authors found a strong correlation of

tissue damage and irradiation-induce IL-6 production. Additional key cytokines reported include IL-1, IL-17 and the CCL20/CCR6 cell migration pathway. The study is carefully carried out and well presented. The following points/comments should be addressed:

1. The RNA expression data clearly highlight the involvement of IL-1, IL-6, IL-17 and CCL20/CCR6 in IRIAD. Yet, the sequence of events is not clearly discussed, which may be important for the selection of appropriate therapeutic targets. Which are the upstream and which are the downstream pathways initiated by skin tissue irradiation?

2. Figure 3 - You state that AKT and ERK1/2 phosphorylation was not affected by deletion of IL-6/IL-6R pathway. Yet, Akt1 is listed as signature gene in the "IL-6 signalling score". Please clarify.

3. IL-17A - I wonder whether the IL-17A data could be moved to the extended data section? Except for the HF phenotype, IL-17A deletion did not do much in this model. It appears that the IL-17A involvement occurs downstream of IL-6?

4. MHCI - The IHC data of MHCI expression are not very clear. What is clear is that irradiation induces profound MHCI expression, and its reduction in IL-6-/- mice is obvious. It would be nice to see evidence for lack of HMCI up-regulation in HF.

5. Figure 6 - Panel G shows a dramatic effect of CCR6 deficiency on IL-6 production. Since IL-6 is primarily expressed in epidermal cells upon skin irradiation, I would like to know more about how CCR6 is linked with IL-6 production in epidermal cells.

6. CXCL14 - CXCL14 is strongly expressed in basal keratinocytes. Just out of curiosity, does IRIAD correlate with changes in CXCL14 expression?

Page 9 (last paragraph) - Explain the expression "intimating"

Referee #2 (Remarks for Author):

Paldor et al reports that a senescence-associated IL-6/IL-1/CCR6-CCL20 inflammatory axis drives alopecia and radiodermatitis induced by radiotherapy. Data from single cell sequencing analysis revealed the upregulation of a cytokine-network associated with senescence, skin inflammation and infiltration of CCR6+ immune cells. By taking advantage of genetically modified mouse models, the authors provide strong evidence for the role of IL6, IL1R and CCR6 in mediating irradiation-induced alopecia and dermatitis (IRIAD). The obtained results support the emerging picture about the pivotal role of CD3+ T cells to the development of autoimmune types of alopecia (i.e. alopecia areata) as well as irradiation-induced dermatitis. As with other types of alopecia, the authors hypothesized that topical inhibitors of JAK as well as suppression of T cells by cyclosporine may benefit IRIAD-patients therapeutically. Their results suggest that inhibition of JAK/STAT-pathway may benefit IRIAD-patients.

A weakness of the study that is based on mouse models. Despite similarities, the immune system and the hair follicle of the mouse significantly differs from that of human. Taken together, the authors provide strong evidence for "IL-6/IL-1/CCR6-CCL20 inflammatory axis", however, how much this is caused by senescence vs tissue, apoptosis, necrosis trauma caused by the irradiation remains unknown. Finally the role of IL-6/IL-1/CCR6-CCL20-pathway is evident in essentially all kind of autoimmune skin inflammation, thus, it does not seem to be specific for IRAD.

Comments:
1. One of the noteworthy aspects of this study is the amount of similarity that can be seen between the inflammatory profile in IRIAD patients and that seen in autoimmune/chronic inflammatory skin diseases, such as psoriasis. Indeed, the role of IL-6/IL-1/CCR6-CCL20-pathway is evident in essentially all kind of autoimmune skin inflammation and thus, does not seem to be specific for IRIAD. What is the authors' comment on this notion? How much of this inflammatory reaction is an unspecific response to trauma and tissue damage and how much is related specifically to irradiation-induced senescence as claimed in the title?

2. The connection of the observed inflammatory pathways to senescence is on a rather weak ground because many of the claims are based on transcriptome signatures associated with senescence. However, senescent cells cannot be identified based only on transcriptome signatures because many elements of the pathways investigated in this model of IRIAD are at least as much related to chronic inflammation than to senescence. Many of the markers that are used in gene enrichment databases for SASP (including Ingenuity) are commonly found in many types of inflammatory diseases. For example, IL-6 is also elevated in a broad range of autoimmune and chronic inflammatory skin diseases and it is not a specific marker of senescent cells. The analysis of urokinase-type plasminogen activator receptor-expression suggests the involvement of senescent cells, but also not an evidence in itself that these cells initiate the cascade of events in IRIAD. I suggest tuning down the message on senescent cells, alternatively providing additional evidence for the initiation of the IL-6/IL-1/CCR6-CCL20 inflammatory axis in alopecia by senescent cells. Additional correlative evidence for this could be obtained by the analysis of additional, more specific markers of senescence (e.g. analysis of SA-β-gal, p16INK4a protein and γH2AX foci), correlation of urokinase-expression with CCL20 and IL-6 and IL-6-scores, or removal of senescent cells by inducible genetic models or senolytics. In the absence of this I suggest a

slight change in the title.

3. The claim about the effect of IL-17A-knockout on IRIAD in the abstract ("Obstruction of cytokine signalling by genetic ablation in IL-6-/-, IL-1R-/-, IL-17-/- ..., strongly ameliorated IRIAD" should be tuned down. Data on Figure 5 show no significant difference either in hair-loss area or epidermal thickness, Ly6+ cells or CD3+ cells

4. Decreased frequency of regulatory T cells is often observed in the active disease state in in autoimmune diseases. I wonder whether the authors could see any change in the frequency of Tregs their scRNAseq data.

5. Please check whether the intended reference(s) is used in this sentence in the discussion "Moreover, oncogene-induced cellular senescence by conditional ectopic H-RasG12V transgene expression in epidermal keratinocytes, involving SASP upregulation, including IL-6 and IL-1b, also reportedly produces marked keratinocyte hyperplasia and acanthosis, together with age-related inflammation (Gallucci et al., 2000)". The referenced article is not about ectopic H-RasG12V transgene expression.

Minor comments:
1. The methods part should be more informative and transparent. For example for cell type-identification it is written that "Cell type of cluster (0,1,2,3,4,5,8,9,10,11,14) were identified using the non-cutoff dependent Gene Set Enrichment Analysis (GSEA) tool (Subramanian, Tamayo et al., 2005) utilizing previously published gene set signatures markers for dermal and epidermal skin cell types.." - the tools/software and the actual signatures used for each cell type should be clear. It is not reasonable to except the reader and the reviewer see through references "(7,10,12,13,15,16,17,18,19,20,21)" to find out which signature was used for "cluster (0,1,2,3,4,5,8,9,10,11,14)" and whether the cell-type identification was correctly performed. It is nowadays common practice to make the scripts used for the bioinformatic analysis available on GitHub for the review process and upon acceptance of the manuscript, to the public.
2. Similarly, for the sake of transparency and reproducibility, datasets should be made available publicly upon the acceptance of the manuscript.

Referee #3 (Comments on Novelty/Model System for Author):

this is a very concise study using scRNASeq in skin cells of naive mice and during an inflammatory condition, which provides an overview about the distribution of skin cell populations and mediator production after an insult. Cellular signaling pathways detected there are verified and validated using knock out models and biochemical investigations. It might be interesting to get more information about the different immune cell populations in skin, but this might be hampered by the sequencing techniques.

Referee #3 (Remarks for Author):

The study of Paldor et al. gives on the one hand a very comprehensive overview of single cell transcriptomics in skin and provides on the other hand a conclusive data set about inflammatory signaling in single skin cell populations after an irradiation-induced insult. The manuscript is very well elaborated and written, the experiments the experiments are very well worked out and conclusive. I have only a few questions.
The authors conclude that IL-1a is produced by keratinocytes and IL-1b by immune cells. This is not new and should be cited somewhere.
In this regard, they find Langerhans cells in the irradiated skin, producing IL-1b. Langerhans cells usually migrate from the skin to the draining lymph nodes after irradiation or other activation. Is there a drop in Langerhans cell numbers seen compared to the non-irradiated controls? This is hard to see from Figure 1B. The same holds true for dermal DC and gamma delta cells. Is there an reduction seen? In that way there should also be an influx of macrophages, monocytes or mo DC after irradiation. Can these cells be distinguished by their expression profile from the DC or Langerhans cells? Where are macrophages "hiding" in the cluster seen in Figure
The authors als refer to a cluster as alpha beta T cells/ILC? Can these two populations be further distinguished. Mediator production should be quite similar, but surface molecules should be somewhat different.

Referee #1 (Remarks for Author - in blue; Authors' response in black):

1. The RNA expression data clearly highlight the involvement of IL-1, IL-6, IL-17 and CCL20/CCR6 in IRIAD. Yet, the sequence of events is not clearly discussed, which may be important for the selection of appropriate therapeutic targets. Which are the upstream and which are the downstream pathways initiated by skin tissue irradiation?

This is an interesting question. Given that upregulation IL-17A, CCL20 and CCR6 mRNA levels appear to precede that of IL-6 by several days, and since IL-1 signaling via NFκB is often considered to be upstream of IL-6, it is tempting to speculate that these factors precede that of IL-6. However, this conclusion may be incorrect, since baseline IL-6 mRNA levels in dermal fibroblasts and in BEC are quite substantial, and upon disruption of interstitial barriers may initiate paracrine IL-6 signaling at earlier times. Thus, based on the present observations, the assignment of a sequence of events may be premature. Nevertheless, the data as they presently stand have strong therapeutic relevance in that they support two important conclusions. First, since the cytokine/chemokine signaling driving radiodermatitis appears to involve a positive feedback loop, timely blockade of any related pathway is likely to significantly ameliorate the pathological outcome. Secondly, since the CCR6 chemokine-directed immune influx is largely driven by CCL20 expression found exclusively within epidermal keratinocytes layers and hair follicles, it is likely that penetration of a therapeutic agent throughout the epidermal skin layers will be sufficient in order to obtain a therapeutic benefit. This latter point is substantiated by the strong effect shown by topical application of the JAK inhibitors, ruxolitinib, and tofacitinib (Fig 3B,C).

2. Figure 3 - You state that AKT and ERK1/2 phosphorylation was not affected by deletion of IL-6/IL-6R pathway. Yet, Akt1 is listed as signature gene in the "IL-6 signaling score". Please clarify.

We agree with the Reviewer on this apparent inconsistency and now believe that the apparent absence of a positive p-AKT signal in the Western blot may point to a technical problem of antibody. For this reason, we have opted to exclude this data from the panel. On the other hand, the Western blot data concerning ERK1/2 phosphorylation does appear to be consistent with the overall picture of IL-6 signaling, with the possible exception that it appears to be elevated a bit earlier than that of p-STAT3 and is reduced by IL-6 and IL-1 deficiencies somewhat less than p-STAT3. Accordingly, we have modified the text to read as follows:

"*Commensurate with IL-6 mRNA upregulation, Western blot analysis indicated that levels of phosphorylated signal transducer and activator of transcription 3 (STAT3), a key mediator of IL-6 signaling (Heinrich, et al., 2003), were prominently elevated beginning at about 10 days post-IR, and was strongly diminished in irradiated IL6$^{-/-}$ mice (Fig 3A). A similar increase in ERK1/2 phosphorylation appeared by day 7.*" [pg 12, para 2]

3. IL-17A - I wonder whether the IL-17A data could be moved to the extended data section? Except for the HF phenotype, IL-17A deletion did not do much in this model. It appears that the IL-17A involvement occurs downstream of IL-6?

We agree with the Reviewer and have moved Figure 5 containing the IL-17A data to a new extended data section, Extended Data Figure EV4.

Regarding the order of events, on face value, the data suggest that IL-17A mRNA upregulation (beginning day 7) might precede that of IL-6 by a short while (IL-6 mRNA upregulation is evident only following day 10). However, as noted above, this does not take into consideration the considerable baseline IL-6 levels in the dermis and the increased IL-6 serum levels appearing within hours following irradiation [see Marmary *et. al.* Cancer Res 76: 1170-80] that may also contribute to this response. Th17 cell activation and IL-17 production are known to be IL-6-dependent, which would also argue that IL-17 upregulation is downstream of IL-6. Also, the induction of SASP factors, including IL-6, is not known to be IL-17 dependent. Nevertheless, the present data also clearly indicates a strong interdependence between the two factors, since the absence of either one strongly diminishes that of the other. Moreover, as discussed in the text [pg 18, para 2], the dependence of both IL-6 and IL-17A upregulation on the CCR6/CCL20 interaction, wherein CCL20 appears to be expressed exclusively in epidermal keratinocytes, implies that the proximity of the IL-17 producing $CCR6^+$ T cells to the IL-6/CCL20 expressing keratinocytes is a key factor in the process.

4. MHCI - The IHC data of MHCI expression are not very clear. What is clear is that irradiation induces profound MHCI expression, and its reduction in IL-6-/- mice is obvious. It would be nice to see evidence for lack of HMCI up-regulation in HF.

The graph to the right in Figure 6, panel F (now Figure 5F) shows the quantification of hair follicles (HF) stained positive for MHCI in irradiated skin of WT *versus* CCR6-/- mice. We have now added a title to the graph, which we hope will make this point clearer to the reader.

5. Figure 6 - Panel G shows a dramatic effect of CCR6 deficiency on IL-6 production. Since IL-6 is primarily expressed in epidermal cells upon skin irradiation, I would like to know more about how CCR6 is linked with IL-6 production in epidermal cells.

This is an interesting question. The literature does not support a direct link between CCR6 and IL-6 production in epidermal cells. However, some reports suggest an indirect link in keratinocytes mediated via IL-17 and IL-22 expressed by T cells. (For instance, see Li *et.al.* J. Inv. Derm. 141, 2021; and Furue *et. al.* Int. J. Mol. Sci. 21, 2020.) IL-17 from γδT cells also reportedly induces IL-6 expression in dermal fibroblasts (Ha *et.al.* PNAS, 2014). Similarly, Tanida *et.al.* (Cytokine, 2009) report that in synovial explants from Rheumatoid arthritis, IL-17 induces IL-6 production that is enhanced significantly by CCL20. Thus, CCR6 may be indirectly linked with IL-6 upregulation via IL-17. In our model, we too find that IL-17A deficiency also strongly diminished IL-6 upregulation, lending support to this notion. However, it is not entirely clear whether this satisfactorily accounts for large reduction in IL-6 production seen in CCR6 *knockout* mice. We currently speculate that the connection between the two factors may be related to the levels of cellular senescence, whose maintenance is IL-6-dependent. Thus, we presently hypothesize that irradiation-induced CCL20 in keratinocytes draws IL-17 and IL-22 expressing $CCR6^+$ T cells to the epidermis and hair follicles (following the loss of their immune privilege status), which in turn enhances the senescence-associated IL-6 in an expanding

positive feedback loop. This expansion perhaps involves a mechanism of paracrine senescence with the participation of additional SASP factors (*e.g.* IL-1, uPAR, PAI-1, *etc.*). Future experiments will be directed to validating this model.

6. CXCL14 - CXCL14 is strongly expressed in basal keratinocytes. Just out of curiosity, does IRIAD correlate with changes in CXCL14 expression?

We, too, were curious about this question. According to our scRNA-seq analysis, Cxcl14 is indeed strongly expressed in naïve skin, particularly in IFE basal keratinocytes (Clusters 0,2,3) and also in outer/inner bulge cells of the hair follicle (Cluster 11). Curiously, irradiation treatment strongly **reduced Cxcl14 expression** in general and in the IFE cells in particular (see UMAP images shown below). For example, in the majority of IFE-BII cells (Cluster-0), Cxcl14 levels are reduced by about 2 logs (p_val_adj = 1.23E-128), although high expression can still be seen in a small number of these cells. Cxcl14 expression also appears to be maintained in about half of the irradiated outer/inner bulge cells (Cluster 11), similar to the naïve cells of this cluster. Only in the small cluster of DN SLN cells (Cluster-13) does Cxcl14 expression appear significantly upregulated following irradiation.

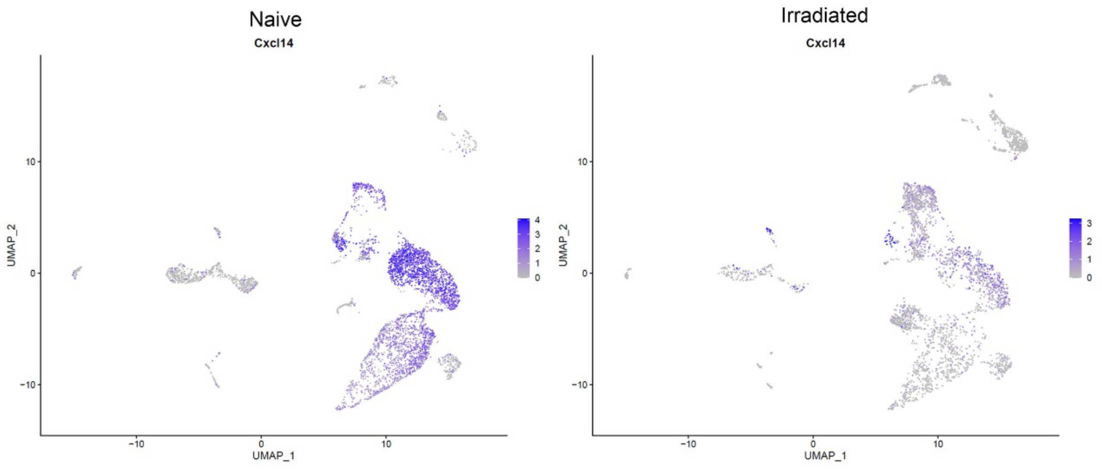

Page 9 (last paragraph) - Explain the expression "intimating"

The word "intimating" is used herein in the sense of a hint [as in intimation: a hint; an obscure or indirect suggestion] in an attempt to explain that the apparent contradiction of the high senescence score together with the cell cycle score is trying to tell us something about these unique cells. That is, the presence of highly expressed cell cycle markers (Mki67) in cells together with a strong senescence score may represent a signature of a unique physiological state. One possible interpretation of this apparent contradiction is that cells in Cluster-2, which normally display high levels of cell division, are forced by irradiation-induced DNA damage to postpone, but not abandon this path. Perhaps they enter into early phases of cell cycle arrest or a potentially reversible phase of senescence, until such time that the cell succeeds to repair their DNA damage – hence a potentially

reversible state – or not, in which case they become more entrenched in their senescence. In order to avoid confusion, we have now replaced the word 'intimating' with 'suggesting'.

Referee #2 (Remarks for Author):

Comments:

1. One of the noteworthy aspects of this study is the amount of similarity that can be seen between the inflammatory profile in IRIAD patients and that seen in autoimmune/chronic inflammatory skin diseases, such as psoriasis. Indeed, the role of IL-6/IL-1/CCR6-CCL20-pathway is evident in essentially all kind of autoimmune skin inflammation and thus, does not seem to be specific for IRIAD. What is the authors' comment on this notion? How much of this inflammatory reaction is an unspecific response to trauma and tissue damage and how much is related specifically to irradiation-induced senescence as claimed in the title?

Yes, we, too, have noted the similarities of IL-6/IL-1/CCR6-CCL20-pathways revealed here within IRIAD and those found in psoriasis and other inflammatory skin diseases. The data and the text allude to the closeness of the relationship between IRIAD and psoriasis multiple times. Thus, the similarities are noted several times in the Results, beginning with the bioinformatics data showing similarities found in multiple cell types with gene expression patterns of "IL-17A in Psoriasis" (Table EV2) to the upregulation of "IL-17-induced psoriasis-related chemokines and antimicrobial peptides, including, S100A8, and S100A9 (Furue, et al., 2020) that localized predominantly within epidermal keratinocyte clusters" (pg 16, para 3). The similarities were also mentioned several times in the Discussion. In order to be more explicit regarding the closeness of IRIAD and psoriasis, including their apparent association with cellular senescence and with keratinocyte hyperplasia, we have now added the following wording to the Discussion.

"In this and other respects, IRIAD shows a high degree of similarity with psoriasis, including the Th17 cytokine-related inflammatory response, as well as the simultaneous presence of keratinocyte hyperplasia together with the presence of cellular senescence in keratinocytes associated with high IGFBP2 expression and p16INK4a upregulation (Mercurio, et al., 2020)." (pg 23, para 2)

However, in further answer to the reviewer's question, within the present data, it is not possible to estimate how much of the inflammatory response is due to senescence or to other causes. Histological analysis also indicated that evidence of skin trauma (parakeratosis), possibly resulting from scratching, was evident at two weeks post-IR (albeit to a lesser extent than senescence) appearing in perhaps 1 of 3 irradiated mice, suggesting that skin trauma may also have contributed somewhat to the inflammatory response. For this and other reasons, we have chosen to use the term 'senescence-associated' instead of a stronger term. Future experiments will be directed at understanding the question of how much the inflammatory response of IRIAD is related specifically to irradiation-induced senescence.

2. The connection of the observed inflammatory pathways to senescence is on a rather weak ground because many of the claims are based on transcriptome signatures associated with senescence. However, senescent cells cannot be identified based only on transcriptome signatures because many elements of the pathways investigated in this model of IRIAD are at least as much related to chronic inflammation than to senescence. Many of the markers that are used in gene enrichment databases for SASP (including Ingenuity) are commonly found in many types of inflammatory diseases. For example, IL-6 is also elevated in a broad range of autoimmune and chronic inflammatory skin diseases and it is not a specific marker of senescent cells. The analysis of urokinase-type plasminogen activator receptor-expression suggests the involvement of senescent cells, but also not an evidence in itself that these cells initiate the cascade of events in IRIAD. I suggest tuning down the message on senescent cells, alternatively providing additional evidence for the initiation of the IL-6/IL-1/CCR6-CCL20 inflammatory axis in alopecia by senescent cells. Additional correlative evidence for this could be obtained by the analysis of additional, more specific markers of senescence (e.g. analysis of SA-β-gal, p16INK4a protein and γH2AX foci), correlation of urokinase expression with CCL20 and IL-6 and IL-6-scores, or removal of senescent cells by inducible genetic models or senolytics. In the absence of this I suggest a slight change in the title.

The authors thank the reviewer for this thought. We agree with the reviewer that many of the markers used in gene enrichment databases for SASP and found to be relevant here are also common to many types of inflammatory diseases. And, perhaps this apparent linkage is an integral point of the findings. Indeed, we find it difficult to ignore the clear association of the rather acute psoriasis-like inflammatory response with the presence of irradiation-induced senescence. While the latter was primarily established by gene enrichment data and supported by immunostaining data of two senescence-related markers (uPAR and lipofuscin), we understand the importance of providing additional evidence to strengthen this association. Therefore, we have now added immunostaining evidence showing strong upregulation of p16INK4a in irradiated WT mice skin in comparison to naïve controls. This analysis shows strong p16INK4a staining in multiple cell types, including a strong presence in differentiated IFE keratinocytes, and a substantial presence in basal keratinocytes, within distinct layers of degenerating hair follicles, and in discrete cells of the dermis (see new Fig EV1D). The p16INK4a immunostaining appears to be largely nuclear, but also cytoplasmic in many cases, in agreement with the literature. This picture of p16INK4a upregulation is in close agreement with that of uPAR immunostaining, lipofuscin staining, and the IPA gene expression analysis of senescence (shown in new Table EV1). Moreover, we found p16INK4a expression to be strongly reduced in irradiated IL-6$^{-/-}$, IL-1R$^{-/-}$, and Ccr6$^{-/-}$ mice in comparison to irradiated WT mice (see new Fig EV1, EV3, Appendix S11A, and Appendix Fig S15). We also now bring evidence showing that loss of IL-1R leads to a strong similar reduction in irradiation-induced hair color depigmentation (see new Appendix Fig S11B), similar to that observed in irradiated IL-6$^{-/-}$ mice shown in Fig EV3C). Altogether, we believe that these observations collectively bring a strong case for the close association of IR-induced senescence with IRIAD.

3. The claim about the effect of IL-17A-knockout on IRIAD in the abstract "Obstruction of cytokine signalling by genetic ablation in IL-6-/-, IL-1R-/-, IL-17-/- ..., strongly ameliorated IRIAD" should be tuned down. Data on Figure 5 show no significant difference either in hair-loss area or epidermal thickness, Ly6+ cells or CD3+ cells

We agree with the reviewer on and have removed the specific reference to IL-17$^{-/-}$ mice from the Abstract.

4. Decreased frequency of regulatory T cells is often observed in the active disease state in autoimmune diseases. I wonder whether the authors could see any change in the frequency of Tregs their scRNAseq data.

As depicted in the UMAP figure below, FoxP3^{+} Tregs identified by scRNA-seq bioinformatics within the population of αβT cells (Cluster-7) represent a relatively small but stable number of cells that was similar for both the naïve (4) and irradiated (5) skin samples. However, since there is a large increase in the overall T cell population following irradiation, apparently due to influx and/or proliferation of cells, the proportion of Tregs decreases substantially relative to the overall T cell population, going from about 10 percent (4/46) to about 1 percent (5/365) of the overall αβT cell population. Following the reviewer's interest in this question, we have now added these observations to the manuscript (pg. 7, para1; pg. 8, para 1; and Appendix Fig S4).

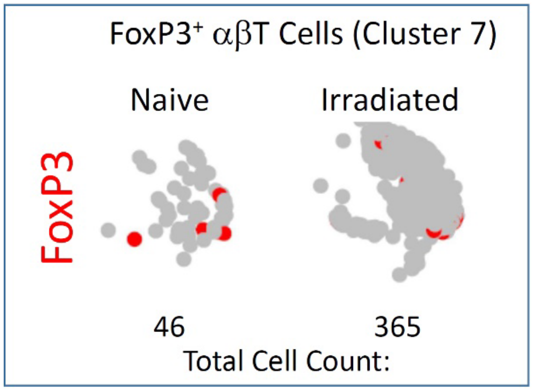

5. Please check whether the intended reference(s) is used in this sentence in the discussion "Moreover, oncogene-induced cellular senescence by conditional ectopic H-RasG12V transgene expression in epidermal keratinocytes, involving SASP upregulation, including IL-6 and IL-1b, also reportedly produces marked keratinocyte hyperplasia and acanthosis, together with age-related inflammation (Gallucci et al., 2000)". The referenced article is not about ectopic H-RasG12V transgene expression.

The authors thank the reviewer for pointing out this error, which appears to have resulted from an erroneous "copy/paste". The intended reference is indeed (Golomb, et al., 2015). The text in the Discussion has been corrected accordingly (pg 21, para 1).

Minor comments:

1. The methods part should be more informative and transparent. For example for cell type-identification it is written that "Cell type of cluster (0,1,2,3,4,5,8,9,10,11,14) were identified using the non-cutoff dependent Gene Set Enrichment Analysis (GSEA) tool (Subramanian, Tamayo et al., 2005)

utilizing previously published gene set signatures markers for dermal and epidermal skin cell types.." - the tools/software and the actual signatures used for each cell type should be clear. It is not reasonable to except the reader and the reviewer see through references (7,10,12,13,15,16,17,18,19,20,21)" to find out which signature was used for "cluster (0,1,2,3,4,5,8,9,10,11,14)" and whether the cell-type identification was correctly performed. It is nowadays common practice to make the scripts used for the bioinformatic analysis available on GitHub for the review process and upon acceptance of the manuscript, to the public.

We apologize to the reviewer for this inconvenience. As specified in the 'Results', GSEA analysis for determination of cell cluster identity was applied using only two reference gene signature data sets. Gene expression data sets from Guerrero-Juarez et al., 2019 were utilized for identification of dermal clusters and data sets from Joost et al., 2016 were applied for epidermal cell type identification. All of the relevant gene set signatures can be found within these two publications and their accompanying supplemental information. The specific clusters for which this applies are also summarized in the GSEA plots shown in Appendix Figure S2. Cells from the subateous gland (Cluster 6) were separately identified according to their unique expression of validated marker genes (*Cers4*/*Cidea*/*Soat1*/Zfp655) (see Figure 1) as previously shown by Joost *et. al, 2016.* The identity of all other clusters were determined using the ImmGen data base comparisons, as specified in the 'Results' and 'Materials and Methods' sections of the text, and also summarized in Appendix Figure S3.

2. Similarly, for the sake of transparency and reproducibility, datasets should be made available publicly upon the acceptance of the manuscript.

The scRNA-seq datasets have now been deposited at GEO: accession number **GSE201447** (https://www.ncbi.nlm.nih.gov/geo/query/acc.cgi?acc=GSE201447). These data can be accessed using the following secure token which has been created to allow review while it remains in private status (pending publication): gpydggmmxhalnml. For the convenience of the reader, naïve and irradiated cluster gene expression datasets derived from the scRNA-seq analysis and utilized in the GSEA analysis will be made available as supplementary information.

Referee #3 (Remarks for Author):

1. The authors conclude that IL-1a is produced by keratinocytes and IL-1b by immune cells. This is not new and should be cited somewhere.

The authors are grateful to the reviewer for pointing out this oversight. Appropriate references citing appropriate previous observations [Luger, et al., 1981; Takashima & Bergstresser, 1996; and Ryan, 2012] have been added to the text (pg 14, para 2).

2. In this regard, they find Langerhans cells in the irradiated skin, producing IL-1b. Langerhans cells usually migrate from the skin to the draining lymph nodes after irradiation or other activation. Is there a drop in Langerhans cell numbers seen compared to the non-irradiated controls? This is hard to see from Figure 1B. The same holds true for dermal DC and gamma delta cells. Is there an reduction seen?

Levels of Langerhans cells (Cluster-18) as a percentage of the total isolated viable cells isolated from whole skin two weeks post-irradiation (55 cells, representing ~2% of the total isolated viable cells) were largely unchanged relative to levels found in non-irradiated control skin (30 cells representing ~1% of the total cells). In contrast, levels of DCs (Cluster-12) in irradiated skin two weeks post-IR (136 cells, ~4% of the total cells) increased about 4-fold in comparison to naive control skin (55 cells, representing ~1% of the total cells). This information can be found in Appendix Supplemental Table I (previously Table EV1).

3. In that way there should also be an influx of macrophages, monocytes or mo DC after irradiation. Can these cells be distinguished by their expression profile from the DC or Langerhans cells? Where are macrophages "hiding" in the cluster seen in Figure?

Comparisons of gene expression profiles of the scRNA-seq data against the ImmGen database identified cluster 12 as being mostly dendritic cell-like, but sharing some similarities with monocyte-derived cells, while cluster 18 represents Langerhans cells (LC), which Guilliams *et. al.* (Nat. Rev. Imm., 14:2014) have proposed to be classified as macrophages of embryonic origin. Expression of individual marker genes within cluster 12 (see UMAP images below) indeed shows that virtually all cells within the 'naïve' cluster express one or more markers associated with both DC and monocyte-derived cells (MC), expressing *Cd74, Hd-Aa*, or *Fcer1g*, that is maintained in the irradiated cells, as well. About half of the naïve Cl-12 cells appear to be cDC2 cells, expressing *Itgam* (Cd11b) and *Sirpa*, while others express MC markers (*Adgre1*, *Itax* (Cd11c), and *C1qc*), with some overlap between the two profiles (see manuscript references: Guilliams, et al., 2014, Saba, et al., 2022). Expression of these markers does not appear to be entirely mutually exclusive, and Cl-12 appears largely negative for *Ly6c*1 and Ly6c2 (data not shown), suggesting that some naive MCs may represent monocyte-derived DCs. Naïve Cl-12 cells were also largely negative for pDC marker genes (not shown), and only 1 cell in the naïve cluster was identified as cDC1 (*Cd207*). Following irradiation, the composition of cluster-12 appears to shift showing higher numbers of cells expressing *Adgre1*, *C1qc*, *Mafb*, and *Ly6c2* and co-expressing *Itgam* and *Sirpa*, perhaps suggesting an influx of monocyte-derived macrophages and dendritic cells in addition to resident cDC2 cells. Following the interest in this question shown by the reviewer, we have now included this information in the manuscript text (see pg 7, para 1; pg 8, para 1, and new Appendix Fig S4).

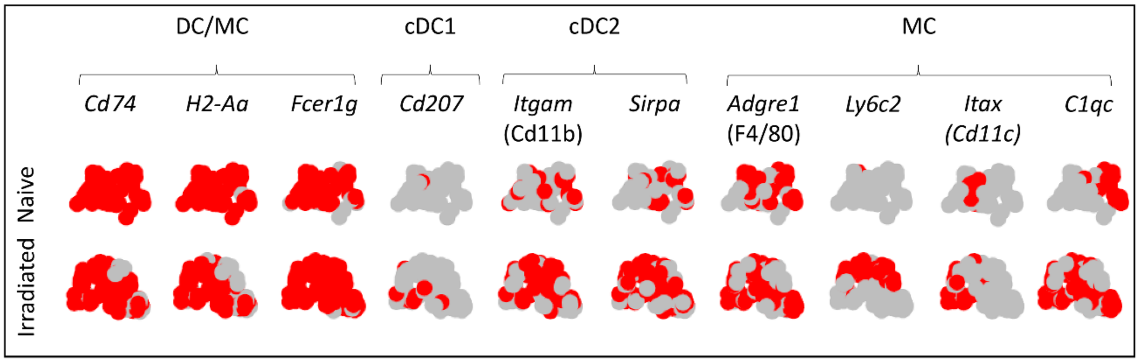

4. The authors also refer to a cluster as alpha beta T cells/ILC? Can these two populations be further distinguished? Mediator production should be quite similar, but surface molecules should be somewhat different.

This is an interesting question. Gene expression analysis for markers in "naïve" Cluster-7 shows that most cells in the cluster express one or more of the *Cd3* alleles (*Cd3d*, *Cd3e*, *Cd3g*), *Cd28*, and/or *Trac*, indicating these cells to most likely be of adaptive T cells lineage. A small number of the cells within the 'naïve' cell cluster appear to be negative for these markers but do appear to express genes reportedly to be more highly expressed in ILCs, (*Klrk1*, *Runx*, or *Fuca1*) [reviewed in Olivia *et.al.*, Cell 165 (2016)]. The $Cd3^{-}$ $Cd28^{-}$ $Trac^{-}$ $Klrk1^{+}$ profile of these cells, which comprise about 10 percent (5/46) of the cells in Cluster-7 (see UMAPs below, circled cells with arrows) suggests that these may represent the ILCs (perhaps ILC1, ILC3, or NK cells) referred to in the ImmGen database comparisons (now also shown in Appendix Fig S4). Interestingly, almost all of the cells in 'irradiated' Cluster-7 express one or more of the T cell marker genes. These cells also appear to express IL-17A, IL-17F, or both (see Fig. EV4 and Appendix Figure S12), and are thus likely to represent Th17 cells. As noted above, about 1% of the cells are $FoxP3^{+}$ Tregs (see response to Reviewer No. 2, question No. 4).

Following the interest shown by the reviewer in this question, we have now modified the manuscript text to include this information as well as changes observed in Cl-7 following irradiation (see pg 7, para 1&2; pg 8, para 1, and new Appendix Fig S4).

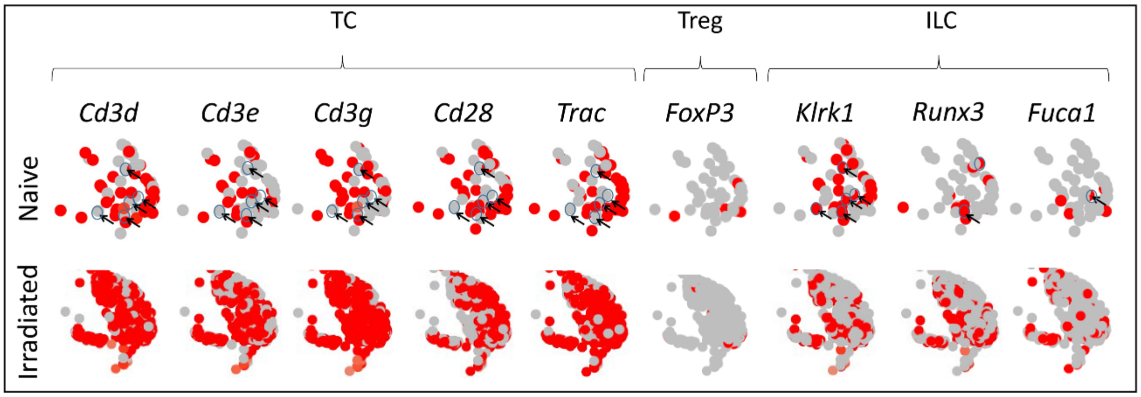

24th May 2022

Dear Dr. Axelrod,

Thank you for the submission of your manuscript to EMBO Molecular Medicine. We have now received the enclosed reports from the three referees who re-reviewed your manuscript. As you will see, they are now supportive of publication, and I am therefore pleased to inform you that we will be able to accept your manuscript once the following editorial points will be addressed:

1/ Main manuscript text:
- Please accept the changes and only keep in track changes mode any new modification.
- The title of your article is a bit long, would you consider shortening it?
- Please note that we can accommodate a maximum of 5 keywords (8 keywords currently on page 1).
- In the Material and Methods, please indicate the antibody dilutions that were used.
- Thank you for depositing your data. Please note that a link seems to be missing ("processed count data are available from https:...")

2/ Figures and Appendix:
- Please indicate in the legends or in the figures (including appendix figures) the exact p= values, not a range. Some people found that to keep the figures clear, providing a supplemental table with all exact p-values was preferable. You are welcome to do this if you want to.
- We would also encourage you to include the source data for figure panels that show essential data. Numerical data should be provided as individual .xls or .csv files (including a tab describing the data). For blots or microscopy, uncropped images should be submitted (using a zip archive if multiple images need to be supplied for one panel). Additional information on source data and instruction on how to label the files are available at
.

3/ As part of the EMBO Publications transparent editorial process initiative (see our Editorial at http://embomolmed.embopress.org/content/2/9/329), EMBO Molecular Medicine will publish online a Review Process File (RPF) to accompany accepted manuscripts.
This file will be published in conjunction with your paper and will include the anonymous referee reports, your point-by-point response and all pertinent correspondence relating to the manuscript. Let us know whether you agree with the publication of the RPF and as here, if you want to remove or not any figures from it prior to publication.
Please note that the Authors checklist will be published at the end of the RPF.

I look forward to receiving your revised manuscript.

Yours sincerely,

Lise Roth

Lise Roth, PhD
Senior Editor
EMBO Molecular Medicine

The system will prompt you to fill in your funding and payment information. This will allow Wiley to send you a quote for the article processing charge (APC) in case of acceptance. This quote takes into account any reduction or fee waivers that you may be eligible for. Authors do not need to pay any fees before their manuscript is accepted and transferred to our publisher.

***** Reviewer's comments *****

Referee #1 (Remarks for Author):

Thank you for responding to my questions which you have answered appropriately.

Referee #2 (Remarks for Author):

The authors have addressed my comments and the manuscript has significantly improved.

Referee #3 (Remarks for Author):

I do not have further questions

The authors performed the requested changes.

1st Jun 2022

Dear Jonathan,

Thank you for submitting the revised files. I am very pleased to inform you that your manuscript is now accepted for publication and will be sent to our publisher to be included in the next available issue of EMBO Molecular Medicine.

Please read below for additional IMPORTANT information regarding your article, its publication and the production process.

Congratulations on your interesting work!

With kind regards,

Lise

Lise Roth, Ph.D
Senior Editor
EMBO Molecular Medicine

Follow us on Twitter @EmboMolMed
Sign up for eTOCs at embopress.org/alertsfeeds

*** *** *** IMPORTANT INFORMATION *** *** ***

SPEED OF PUBLICATION
The journal aims for rapid publication of papers, using using the advance online publication "Early View" to expedite the process: A properly copy-edited and formatted version will be published as "Early View" after the proofs have been corrected. Please help the Editors and publisher avoid delays by providing e-mail address(es), telephone and fax numbers at which author(s) can be contacted.

Should you be planning a Press Release on your article, please get in contact with embomolmed@wiley.com as early as possible, in order to coordinate publication and release dates.

LICENSE AND PAYMENT:

All articles published in EMBO Molecular Medicine are fully open access: immediately and freely available to read, download and share.

EMBO Molecular Medicine charges an article processing charge (APC) to cover the publication costs. You, as the corresponding author for this manuscript, should have already received a quote with the article processing fee separately. Please let us know in case this quote has not been received.

Once your article is at Wiley for editorial production you will receive an email from Wiley's Author Services system, which will ask you to log in and will present you with the publication license form for completion. Within the same system the publication fee can be paid by credit card, an invoice, pro forma invoice or purchase order can be requested.

Payment of the publication charge and the signed Open Access Agreement form must be received before the article can be published online.

PROOFS

You will receive the proofs by e-mail approximately 2 weeks after all relevant files have been sent o our Production Office. Please return them within 48 hours and if there should be any problems, please contact the production office at embopressproduction@wiley.com.

Please inform us if there is likely to be any difficulty in reaching you at the above address at that time. Failure to meet our

deadlines may result in a delay of publication.

All further communications concerning your paper proofs should quote reference number EMM-2021-15653-V3 and be directed to the production office at embopressproduction@wiley.com.

EMBO Press Author Checklist

Corresponding Author Name: Jonathan H. Axelrod	
Journal Submitted to: EMBO Molecular Medicine	
Manuscript Number: EMM-2021-15653	

USEFUL LINKS FOR COMPLETING THIS FORM
The EMBO Journal - Author Guidelines
EMBO Reports - Author Guidelines
Molecular Systems Biology - Author Guidelines
EMBO Molecular Medicine - Author Guidelines

Reporting Checklist for Life Science Articles (updated January 2022)
This checklist is adapted from Materials Design Analysis Reporting (MDAR) Checklist for Authors. MDAR establishes a minimum set of requirements in transparent reporting in the life sciences (see Statement of Task: 10.31222/osf.io/9sm4x). Please follow the journal's guidelines in preparing your manuscript.
Please note that a copy of this checklist will be published alongside your article.

Abridged guidelines for figures
1. Data
The data shown in figures should satisfy the following conditions:
- the data were obtained and processed according to the field's best practice and are presented to reflect the results of the experiments in an accurate and unbiased manner.
- ideally, figure panels should include only measurements that are directly comparable to each other and obtained with the same assay.
- plots include clearly labeled error bars for independent experiments and sample sizes. Unless justified, error bars should not be shown for technical replicates.
- if n<5, the individual data points from each experiment should be plotted. Any statistical test employed should be justified.
- Source Data should be included to report the data underlying figures according to the guidelines set out in the authorship guidelines on Data Presentation.

2. Captions
Each figure caption should contain the following information, for each panel where they are relevant:
- a specification of the experimental system investigated (eg cell line, species name).
- the assay(s) and method(s) used to carry out the reported observations and measurements.
- an explicit mention of the biological and chemical entity(ies) that are being measured.
- an explicit mention of the biological and chemical entity(ies) that are altered/varied/perturbed in a controlled manner.
- the exact sample size (n) for each experimental group/condition, given as a number, not a range;
- a description of the sample collection allowing the reader to understand whether the samples represent technical or biological replicates (including how many animals, litters, cultures, etc.).
- a statement of how many times the experiment shown was independently replicated in the laboratory.
- definitions of statistical methods and measures:
  - common tests, such as t-test (please specify whether paired vs. unpaired), simple χ2 tests, Wilcoxon and Mann-Whitney tests, can be unambiguously identified by name only, but more complex techniques should be described in the methods section;
  - are tests one-sided or two-sided?
  - are there adjustments for multiple comparisons?
  - exact statistical test results, e.g., P values = x but not P values < x;
  - definition of 'center values' as median or average;
  - definition of error bars as s.d. or s.e.m.

Please complete ALL of the questions below.
Select "Not Applicable" only when the requested information is not relevant for your study.

Materials

Newly Created Materials	Information included in the manuscript?	In which section is the information available? (Reagents and Tools Table, Materials and Methods, Figures, Data Availability Section)
New materials and reagents need to be available; do any restrictions apply?	Not Applicable	

Antibodies	Information included in the manuscript?	In which section is the information available? (Reagents and Tools Table, Materials and Methods, Figures, Data Availability Section)
For **antibodies** provide the following information:<br>- Commercial antibodies: RRID (if possible) or supplier name, catalogue number and or/clone number<br>- Non-commercial: RRID or citation	Yes	Materials and Methods

DNA and RNA sequences	Information included in the manuscript?	In which section is the information available? (Reagents and Tools Table, Materials and Methods, Figures, Data Availability Section)
**Short novel DNA or RNA including primers, probes**: provide the sequences.	Yes	Materials and Methods

Cell materials	Information included in the manuscript?	In which section is the information available? (Reagents and Tools Table, Materials and Methods, Figures, Data Availability Section)
**Cell lines:** Provide species information, strain. Provide accession number in repository **OR** supplier name, catalog number, clone number, and/**OR** RRID.	Not Applicable	
**Primary cultures:** Provide species, strain, sex of origin, genetic modification status.	Not Applicable	
Report if the cell lines were recently **authenticated** (e.g., by STR profiling) and tested for mycoplasma contamination.	Not Applicable	

Experimental animals	Information included in the manuscript?	In which section is the information available? (Reagents and Tools Table, Materials and Methods, Figures, Data Availability Section)
**Laboratory animals or Model organisms:** Provide species, strain, sex, age, genetic modification status. Provide accession number in repository **OR** supplier name, catalog number, clone number, **OR** RRID.	Yes	Materials and Methods
**Animal observed in or captured from the field:** Provide species, sex, and age where possible.	Not Applicable	
Please detail **housing and husbandry conditions**.	Yes	Materials and Methods

Plants and microbes	Information included in the manuscript?	In which section is the information available? (Reagents and Tools Table, Materials and Methods, Figures, Data Availability Section)
**Plants:** provide species and strain, ecotype and cultivar where relevant, unique accession number if available, and source (including location for collected wild specimens).	Not Applicable	
**Microbes:** provide species and strain, unique accession number if available, and source.	Not Applicable	

Human research participants	Information included in the manuscript?	In which section is the information available? (Reagents and Tools Table, Materials and Methods, Figures, Data Availability Section)
If collected and within the bounds of privacy constraints report on age, sex and gender or ethnicity for all study participants.	Not Applicable	

Core facilities	Information included in the manuscript?	In which section is the information available? (Reagents and Tools Table, Materials and Methods, Figures, Data Availability Section)
If your work benefited from core facilities, was their service mentioned in the acknowledgments section?	Yes	Acknowledgments

Design

Study protocol	Information included in the manuscript?	In which section is the information available? (Reagents and Tools Table, Materials and Methods, Figures, Data Availability Section)
If study protocol has been **pre-registered, provide DOI in the manuscript**. For clinical trials, provide the trial registration number **OR** cite DOI.	Not Applicable	
Report the **clinical trial registration number** (at ClinicalTrials.gov or equivalent), where applicable.	Not Applicable	

Laboratory protocol	Information included in the manuscript?	In which section is the information available? (Reagents and Tools Table, Materials and Methods, Figures, Data Availability Section)
Provide DOI OR other citation details if **external detailed step-by-step protocols** are available.	Not Applicable	

Experimental study design and statistics	Information included in the manuscript?	In which section is the information available? (Reagents and Tools Table, Materials and Methods, Figures, Data Availability Section)
Include a statement about **sample size** estimate even if no statistical methods were used.	Yes	Figures
Were any steps taken to minimize the effects of subjective bias when allocating animals/samples to treatment (e.g. **randomization procedure**)? If yes, have they been described?	Not Applicable	
Include a statement about **blinding** even if no blinding was done.	Yes	Materials and Methods
Describe **inclusion/exclusion criteria** if samples or animals were excluded from the analysis. Were the criteria pre-established?<br><br>If sample or data points were omitted from analysis, report if this was due to attrition or intentional exclusion and provide justification.	Yes	Materials and Methods / Results
For every figure, are **statistical tests** justified as appropriate? Do the data meet the assumptions of the tests (e.g., normal distribution)? Describe any methods used to assess it. Is there an estimate of variation within each group of data? Is the variance similar between the groups that are being statistically compared?	Yes	Materials and Methods

Sample definition and in-laboratory replication	Information included in the manuscript?	In which section is the information available? (Reagents and Tools Table, Materials and Methods, Figures, Data Availability Section)
In the figure legends: state number of times the experiment was **replicated** in laboratory.	Yes	Figures
In the figure legends: define whether data describe **technical or biological replicates**.	Yes	Figures

Ethics

Ethics	Information included in the manuscript?	In which section is the information available? (Reagents and Tools Table, Materials and Methods, Figures, Data Availability Section)
Studies involving **human participants**: State details of **authority granting ethics approval** (IRB or equivalent committee(s), provide reference number for approval.	Not Applicable	
Studies involving **human participants**: Include a statement confirming that **informed consent** was obtained from all subjects and that the experiments conformed to the principles set out in the WMA Declaration of Helsinki and the Department of Health and Human Services Belmont Report.	Not Applicable	
Studies involving **human participants:** For publication of **patient photos**, include a statement confirming that consent to publish was obtained.	Not Applicable	
Studies involving experimental **animals**: State details of **authority granting ethics approval** (IRB or equivalent committee(s), provide reference number for approval. Include a statement of compliance with ethical regulations.	Yes	Materials and Methods
Studies involving **specimen and field samples:** State if relevant **permits** obtained, provide details of authority approving study; if none were required, explain why.	Not Applicable	

Dual Use Research of Concern (DURC)	Information included in the manuscript?	In which section is the information available? (Reagents and Tools Table, Materials and Methods, Figures, Data Availability Section)
Could your study fall under dual use research restrictions? Please check biosecurity documents and list of **select agents and toxins** (CDC): https://www.selectagents.gov/sat/list.htm	Not Applicable	
If you used a select agent, is the security level of the lab appropriate and reported in the manuscript?	Not Applicable	
If a study is subject to dual use research of concern regulations, is the name of the **authority granting approval and reference number** for the regulatory approval provided in the manuscript?	Not Applicable	

Reporting

The MDAR framework recommends adoption of discipline-specific guidelines, established and endorsed through community initiatives. Journals have their own policy about requiring specific guidelines and recommendations to complement MDAR.

Adherence to community standards	Information included in the manuscript?	In which section is the information available? (Reagents and Tools Table, Materials and Methods, Figures, Data Availability Section)
State if relevant guidelines or checklists (e.g., **ICMJE, MIBBI, ARRIVE, PRISMA**) have been followed or provided.	Not Applicable	
For **tumor marker prognostic studies,** we recommend that you follow the **REMARK** reporting guidelines (see link list at top right). See author guidelines, under 'Reporting Guidelines'. Please confirm you have followed these guidelines.	Not Applicable	
For **phase II and III randomized controlled trials**, please refer to the **CONSORT** flow diagram (see link list at top right) and submit the CONSORT checklist (see link list at top right) with your submission. See author guidelines, under 'Reporting Guidelines'. Please confirm you have submitted this list.	Not Applicable	

Data Availability

Data availability	Information included in the manuscript?	In which section is the information available? (Reagents and Tools Table, Materials and Methods, Figures, Data Availability Section)
Have **primary datasets** been deposited according to the journal's guidelines (see 'Data Deposition' section) and the respective accession numbers provided in the Data Availability Section?	Yes	Materials and Methods
Were **human clinical and genomic datasets** deposited in a public access-controlled repository in accordance to ethical obligations to the patients and to the applicable consent agreement?	Not Applicable	
Are **computational models** that are central and integral to a study available without restrictions in a machine-readable form? Were the relevant accession numbers or links provided?	Not Applicable	
If publicly available data were reused, provide the respective **data citations in the reference list**.	Not Applicable	
